# High-Spin State of a Ferrocene Electron Donor Revealed by Optical and X-ray Transient Absorption Spectroscopy

**DOI:** 10.1021/jacs.4c05646

**Published:** 2024-07-25

**Authors:** John H. Burke, Dae Young Bae, Rachel F. Wallick, Conner P. Dykstra, Thomas C. Rossi, Laura E. Smith, Clare A. Leahy, Richard D. Schaller, Liviu M. Mirica, Josh Vura-Weis, Renske M. van der Veen

**Affiliations:** † Department of Chemistry, University of Illinois at Urbana−Champaign, Urbana, Illinois 61801, United States; ‡ Department of Atomic-Scale Dynamics in Light-Energy Conversion, Helmholtz-Zentrum Berlin für Materialien und Energie, Berlin 14109, Germany; § Department of Chemistry, Northwestern University, Evanston, Illinois 60208, United States; ∥ Center for Nanoscale Materials, Argonne National Laboratory, Lemont, Illinois 60439, United States; ⊥ Institute of Optics and Atomic Physics, Technical University of Berlin, 10623 Berlin, Germany

## Abstract

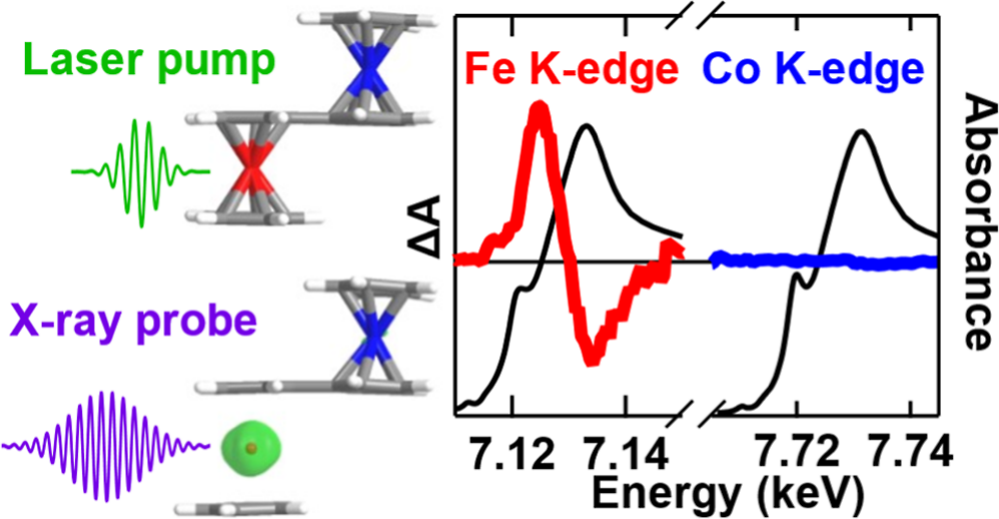

Ferrocene is one of the most common electron donors, and mapping its ligand-field excited states is critical to designing donor–acceptor (D–A) molecules with long-lived charge transfer states. Although ^3^(d–d) states are commonly invoked in the photophysics of ferrocene complexes, mention of the high-spin ^5^(d–d) state is scarce. Here, we provide clear evidence of ^5^(d–d) formation in a bimetallic D–A molecule, ferrocenyl cobaltocenium hexafluorophosphate ([FcCc]PF_6_). Femtosecond optical transient absorption (OTA) spectroscopy reveals two distinct electronic excited states with 30 and 500 ps lifetimes. Using a combination of ultraviolet, visible, near-infrared, and short-wave infrared probe pulses, we capture the spectral features of these states over an ultrabroadband range spanning 320 to 2200 nm. Time-dependent density functional theory (DFT) calculations of the lowest triplet and quintet states, both primarily Fe(II) (d–d) in character, qualitatively agree with the experimental OTA spectra, allowing us to assign the 30 ps state as the ^3^(d–d) state and the 500 ps state as the high-spin ^5^(d–d) state. To confirm the ferrocene-centered high-spin character of the 500 ps state, we performed X-ray transient absorption (XTA) spectroscopy at the Fe and Co K edges. The Fe K-edge XTA spectrum at 150 ps shows a red shift of the absorption edge that is consistent with an Fe(II) high-spin state, as supported by ab initio calculations. The transient signal detected at the Co K-edge is 50× weaker, confirming the ferrocene-centered character of the excited state. Fitting of the transient extended X-ray absorption fine structure region yields an Fe–C bond length increase of 0.25 ± 0.1 Å in the excited state, as expected for the high-spin state based on DFT. Altogether, these results demonstrate that the high-spin state of ferrocene should be considered when designing donor–acceptor assemblies for photocatalysis and photovoltaics.

## Introduction

In photocatalysis and photovoltaics, donor–acceptor (D–A) molecules enable solar energy conversion by absorbing photons and forming charge-transfer (CT) excited states. CT states facilitate extraction of charge and energy, and their lifetime plays a critical role in the photocatalytic/photovoltaic efficiency. Ferrocene (Fe(C_5_H_5_)_2_) is one of the most common electron donors in D–A molecules, and it has been coupled to a diverse array of organic^1−15^ and organometallic^16−19^ electron acceptors. This organometallic building block finds such popularity due to its exceptional stability and well-characterized single-electron chemistry.^20^ However, the local excited states of ferrocene are still not fully characterized. In D–A molecules, local excited states of the donor or acceptor, such as triplet excited states^21^ in organic molecules and ligand-field excited states^22^ in transition metal complexes, can present undesired nonradiative decay pathways. Nonradiative decay, in which the photon energy is released to the surroundings as heat, is a major energy-loss channel in solar energy conversion. Design of D–A molecules with long-lived CT states requires careful consideration of the energetics of local excited states to steer the dynamics away from such nonradiative pathways. To this end, characterizing the ligand-field excited states of ferrocene is critical for its successful implementation as a donor in D–A molecules.

The ^1^(d–d) and ^3^(d–d) ligand-field excited states of ferrocene have received significant attention. In the UV–vis absorption spectrum of ferrocene, the Laporte-forbidden transition to the ^1^(d–d) state is observed at 2.7 eV, and the spin-forbidden transition to the ^3^(d–d) state is observed at 2.1 eV.^23,24^ Early flash photolysis experiments highlighted the importance of the ^3^(d–d) state by demonstrating that ferrocene quenches the triplet excited states of molecules with energies in the range of 1.9–3.1 eV.^25−27^ With modern ultrafast lasers, surprisingly few optical transient absorption (OTA) spectroscopy experiments of ferrocene have been published. However, a nanosecond-resolved OTA study of ferrocene showed evidence of long-lived transient species that were assigned to ^1^(d–d) states and ^3^(d–d) states.^28^ On the femtosecond time scale, ferrocene is mostly studied as a donor in D–A molecules. In their studies of an organometallic D–A molecule with two ethynylferrocene donors coupled to a titanocene acceptor, Livshits et al.^17^ pointed out that the metal-to-metal charge transfer (MMCT) state is strongly mixed with the ^1^(d–d) state of the ferrocene donor. Femtosecond-resolved OTA studies of that molecule and its CuBr complex, where the Cu(I) ion binds between the two alkynes, revealed excited states of ∼10 ps and ∼60 ns lifetimes that were assigned as the mixed ^1^MMCT/^1^(d–d) state and ^3^(d–d) state, respectively.^17^ Several other examples of the ^3^(d–d) state are found in OTA studies of D–A molecules with ferrocene donors and organic acceptors^2,8,29^ Taken together, these studies provide a detailed mapping of the ^1^(d–d) and ^3^(d–d) ligand-field excited states of ferrocene. However, completely characterizing the local excited states of ferrocene requires consideration of the high-spin ^5^(d–d) state.

The high-spin ^5^(d–d) state of ferrocene is far less studied than the ^1^(d–d) and ^3^(d–d) states. Recently, formation of a ferrocenium dioxygen complex adsorbed in a metal–organic framework was proposed to occur through a reactive high-spin ferrocene intermediate that was formed by conformational changes in the pores and magnetic interactions with the Co(II) host.^30^ However, most other mentions of this state are largely confined to the electronic structure theory literature.^31−36^ Interestingly, density functional theory (DFT) typically predicts the high-spin ^5^(d–d) state to be within a few kcal/mol of the intermediate-spin ^3^(d–d) state,^34,36^ suggesting that it is energetically accessible in many photoexcited D–A molecules. Despite this possibility, we could only find one direct experimental observation of the high-spin state. Einaga et al.^37,38^ performed a series of Mössbauer spectroscopic studies of low-temperature (130 or 20 K) films doped with Fe(C_5_H_5_)_2_·CCl_4_ CT complexes. Under steady-state photoirradiation, the complex forms a CT state of the form (Fe(C_5_H_5_)_2_^+^Cl^–^)·CCl_3_, as evidenced by the rise of an Fe(III) signal. Decay of the Fe(III) signal was accompanied by the growth of a high-spin Fe(II) signal, suggesting charge recombination to high-spin ferrocene, which was metastable at low temperature. Considering the above experimental observations and theoretical results, we postulated that the high-spin state of ferrocene could also be relevant to the ultrafast photophysics of D–A molecules in solution at room temperature. Here, we demonstrate formation of this state in an organometallic D–A system, ferrocenyl cobaltocenium hexafluorophosphate, [FcCc]PF_6_.

[FcCc]PF_6_ is a heterobimetallocene with a ferrocene donor and cobaltocenium acceptor. This complex was first synthesized by Schwarzhans and co-workers^39,40^ and later studied by Warratz et al.^41^ with UV–vis absorption spectroscopy, time dependent (TD-)DFT, and Mössbauer spectroscopy. This molecule has a low-spin Fe(II)Co(III) ground state and an intense MMCT absorption band at 550 nm that forms the Fe(III)Co(II) ^1^MMCT state. This mixed-valence complex is Robin-Day Class I (localized)^42^ due to the large redox asymmetry between the ferrocene donor and cobaltocenium acceptor. In this work, we use ultrafast optical and hard X-ray transient absorption spectroscopy to characterize the excited-state dynamics that follow MMCT excitation. OTA spectroscopy points to the formation of a ^3^(d–d) state following charge recombination and the subsequent population of a high-spin ^5^(d–d) state. Hard X-ray transient absorption (XTA) spectroscopy confirms the high-spin assignment with spin-state and elemental specificity. At the Fe K-edge, the XTA spectrum shows signatures of an edge shift and bond-length expansion, in line with high-spin state formation. At the Co K edge, the XTA signal is 50× weaker, showing that the ^5^(d–d) state is localized to the ferrocene donor. Observation of the ferrocene-centered high-spin state shows that this state is relevant to the photophysics of D–A molecules and should be considered when designing molecules with long-lived CT states for photocatalysis and photovoltaics.

## Experimental and Computational Methods

### Synthetic Procedures

All air- and moisture-sensitive operations, including basic Schlenk and glovebox techniques, were performed using oven-dried glassware under a nitrogen atmosphere if not indicated otherwise. All reagents for which the syntheses are not given were purchased from Sigma-Aldrich, Acros, STREM, or Pressure Chemical and were used as received without further purification. Solvents were purified prior to use by passing through a column of activated alumina using an MBRAUN SPS. The synthesis of ferrocenyl cobaltocenium hexafluorophosphate followed a slightly modified procedure from the literature.^40,41^

### Lithioferrocene

Ferrocenyl bromide (6.03 g, 22.8 mmol, 1.0 equiv) was added to a 500 mL round-bottom flask, followed by the addition of 150 mL of diethyl ether at −78 °C. An equivalent amount of *n*-butyllithium (14.2 mL of 1.6 M in hexanes, 22.8 mmol, 1.0 equiv) was then gradually added dropwise over 10 min to the flask. The reaction mixture was gently warmed to 0 °C using an ice water bath with continuous stirring. An orange precipitate was observed at −13 °C. After reaching 0 °C, the solution was stirred for an additional 20 min. The resulting orange precipitate was subsequently isolated by vacuum filtration and dried, yielding orange lithioferrocene (3.83 g, 88%).

### Ferrocenyl Cobaltocenium Hexafluorophosphate

Cobaltocenium hexafluorophosphate (6.33 g, 19.0 mmol, 0.95 equiv relative to FcLi) is added to a 1 L round-bottom flask, followed by the addition of 200 mL of THF. The mixture was then cooled to −78 °C. A solution of lithioferrocene (3.83 g, 20.0 mmol, 1.0 equiv) in THF (200 mL) was prepared and added dropwise to the reaction flask in the dark. The reaction mixture was stirred for 14 h in the dark, with the flask covered in aluminum foil, and gradually allowed to warm to room temperature. A red-colored homogeneous solution was obtained, and volatiles were dried in vacuo in the dark. (Note: This is a light-sensitive reaction.) The resulting dried solid was dissolved in 150 mL of dichloromethane and filtered through a pad of Celite. To the filtrate, triphenylmethyl hexafluorophosphate (7.36 g, 19.0 mmol, 0.95 equiv) was added. The solution rapidly changed to a deep blue color within minutes of stirring at room temperature, and the mixture was stirred for an additional 30 min. (Note: the deep blue colored solution is no longer light-sensitive.) The reaction flask was then removed from the glovebox, and volatiles were dried in vacuo. The resulting solid was washed sequentially with diethyl ether (10 mL) and then with distilled cold water (3 mL) nine times, until the washing solution is colorless. The washed solid was dried in vacuo to afford the dark blue product (9.76 g, 96%). The spectral data were in agreement with the literature values.^41^^1^H NMR (CD_3_CN, 500 MHz): δ (ppm): 5.84 (br, 2H), 5.65 (br, 2H), 5.37 (br, 5H), 4.74 (br, 2H), 4.56 (br, 2H), 4.08 (br, 5H).

### Computational Methods

DFT calculations were performed in Gaussian 16.^43^ All DFT calculations presented in the main text were performed using the B3PW91^44,45^ functional and 6-311+G(d)^46−49^ basis sets. Implicit solvation in acetonitrile was included through the polarizable continuum model (PCM).^50^ Supplemental calculations in the Supporting Information utilized the B3LYP^44,51^ functional and 6-311+G(d) or LANL2DZ^52,53^ basis sets. Structures were optimized to stationary points which were determined to be local minima by performing frequency calculations and verifying the absence of imaginary frequencies. Single point and time-dependent DFT calculations were performed at the same level of theory as the geometry optimizations. Thermal corrections^54^ to free energies assumed an ideal gas at 298.15 K and 1 atm. Time-dependent (TD-)DFT calculations found the first 150 excited states. Molecular geometries were visualized with GaussView 6. Canonical and natural transition orbitals were visualized with isovalues of 0.02 using Chemissian. Broadened TD-DFT spectra in extinction coefficient units of M^–1^ cm^–1^ were calculated with Gaussian lineshapes of σ = 0.3 eV according to Equation S1.^55^

### OTA Spectroscopy

The ultraviolet–visible (UV–vis) OTA measurements were performed on a home-built setup in the Materials Research Laboratory Central Research Facilities at the University of Illinois. Briefly, an 800 nm laser pulse was generated in a Ti:sapphire regenerative amplifier (1 kHz, 120 fs, Spitfire, Spectra-Physics). A small portion of the pulse was split from the beam, sent through a retroreflector mounted on a motorized delay stage and focused into a CaF_2_ crystal to generate a broadband probe in the UV–vis range from 320 to 650 nm. The delay stage can be moved in steps of ∼40 fs up to a ∼ 5 ns delay time. The remainder of the pulse was sent into an optical parametric amplifier (TOPAS Twins/NirUVis, Light Conversion) to generate the pump pulse. The pump pulse was sent through a chopper operating at 500 Hz to cut out every other pulse. The broadband probe beam was collimated and focused to a spot size of ∼10 μm fwhm in the sample plane using off-axis parabolic mirrors, and the pump beam was focused using a spherical mirror to be 377 μm in the sample plane with a perpendicular relative pump–probe polarization. The transmitted probe beam is coupled into a multimode optical fiber, which is coupled into an Ultrafast Systems spectrometer which disperses and focuses the beam onto a CMOS detector. Every individual pump-on (*I*_p_) and pump-off (*I*_o_) probe spectrum is collected, allowing for shot-to-shot detection. The transient signal is then calculated as Δ*A*(λ,*t*) = log10(*I*_p_/*I*_o_). Individual scans used 0.1 s of averaging at each time point and 7 to 10 individual scans were averaged together to increase signal-to-noise. The sample consisted of 0.2 mM [FcCc]PF_6_ in a 2 mm cell.

Additional OTA experiments with visible (vis), near-infrared (NIR), and short-wave infrared (SWIR) were performed at the Center for Nanoscale Materials at Argonne National Laboratory. A commercial titanium:sapphire regeneratively amplified laser (Spectra Physics Spitfire) was employed to produce 35 fs pulses at 800 nm at a 2 kHz repetition rate. A portion of the output was directed to an optical parametric amplifier and sum frequency generation stage (Light Conversion) to produce pump pulses at 515 nm, which were depolarized to reduce anisotropic effects. These pulses were reduced in repetition rate to 1 kHz via a mechanical chopper, controlled for fluence with a variable neutral density wheel, and focused into the sample with a 594 μm 1/e^2^ diameter spot size at a fluence of 1.7 mJ/cm^2^ (vis & NIR probe experiments) or 3.3 mJ/cm^2^ (SWIR experiment). Probe pulses were produced from a portion of the 800 nm laser output either in the visible wavelength range by focusing into a 2 mm thick sapphire crystal, in the NIR range by focusing into an 8 mm thick sapphire crystal, or in the SWIR by using the output of an optical parametric amplifier. Probe pulses were focused into the sample to produce a ∼80 μm diameter spot and were detected on a single-shot basis to compare pump on versus pump off conditions. Time delays were produced by delaying the probe beam using a mechanical delay line and retroreflector. Individual scans used 2–3 s of averaging at each time point and 4 to 8 individual scans were averaged together to increase signal-to-noise. The sample consisted of 1 mM [FcCc]PF_6_ in acetonitrile in a cell of 1 mm path length. Global fitting of OTA data was performed in Igor Pro.

### X-ray Transient Absorption Spectroscopy

Hard X-ray transient absorption spectroscopy was performed at sector 11-ID-D of the Advanced Photon Source. The setup has been described in detail elsewhere.^56^ Briefly, X-rays in the keV range are generated from an undulator in the electron storage ring operating in 24-bunch mode. The X-rays are monochromatized with a double Si(111) crystal monochromator, and focused with a toroidal mirror to a spot size of 500 μm (H) × 200 μm (V) (1/e^2^) at the sample position. The pump pulse comprises the 515 nm output of an optical parametric amplifier pumped by a femtosecond Ti:sapphire amplifier (Coherent Legend Elite Duo seeded by Coherent Micra-5) operating at a repetition rate of 3 kHz. The pump laser was focused to a 1/*e*^2^ diameter of 864 μm (H) × 519 μm (V) at the sample position, at a fluence of 20 mJ/cm^2^. The sample environment is a 700 μm cylindrical liquid jet of 5 mM ferrocenyl cobaltocenium hexafluorophosphate in acetonitrile. The 80 mL sample is continuously circulated from a sample reservoir with a peristaltic pump through a 700 μm stainless steel nozzle at a flow rate of 100 mL/min. Solvent-saturated N_2_ gas was bubbled through the solution and the sample was changed approximately every 12 h. The X-ray transient absorption signal was collected in total fluorescence yield using two APD detectors at 90 deg from the X-ray probe beam. Soller slits and Z-1 filters were utilized to minimize the contribution from elastic scatter. A reference APD before the sample, which collected elastic scatter from air, was used for normalization of the APD signals. The period of the 6.53 MHz X-ray pulses was 152 ns, and the period of the 3 kHz laser pulses was 0.333 ms. The laser pulse was synchronized to an X-ray pulse and the time delay relative to the latter was controlled with two electronic delay generators (Colby Instruments and Highland). This synchronized X-ray pulse was used as the pumped signal and the remaining X-ray pulses that interrogated the sample between laser pulses were used as the unpumped signal. Spatial and temporal overlap of the X-ray and laser pulses were found using a reference sample of 3 mM [Fe(bpy)_3_]BF_4_ in acetonitrile. The spectra in the main text have been shifted by +0.6 eV at the Fe edge and −0.8 eV at the Co edge as determined by calibration with Fe and Co metal foil references (Exafs Materials).

Processing of the static extended X-ray absorption fine structure (EXAFS) spectrum was performed in ATHENA. EXAFS fitting was performed in ARTEMIS. For more details, see Supporting Information Section 4.4.

TD-DFT calculations of the Fe 1s pre-edge of [FcCc]^+^ were performed in ORCA. The calculations restricted the initial orbital to the Fe 1s orbital (which is the second orbital, above the Co 1s). The calculations employed the B3LYP functional with def2-TZVP^57^ basis set and def2/J^58^ auxiliary basis set. Geometry optimizations were performed before the TD-DFT calculations. TD-DFT sticks were broadened with Gaussian lineshapes of 2 eV width.

Real-space Green’s function theory was used to simulate the K-edge absorption spectra. The calculations were carried out in the FEFF10 software package.^59,60^ These calculations employ a muffin-tin potential based on the atomic coordinates fed into the program. As input geometries, we used the DFT geometry-optimized coordinates of the S_0_, T_1_, or Q_1_ state of [FcCc]^+^ at the B3PW91/6-311+G(d) PCM (acetonitrile) level. Spin degrees of freedom are not included in the calculations. The overall positive charge of the molecule was accounted for with the ION card, initially distributing the +1 charge evenly across all constituent atoms. The core-hole was treated by the final state rule. A cluster radius of 9 Å, centered around the absorbing atom, was selected for all self-consistent field (SCF) and scattering path calculations to encompass every atom in the molecule. For calculation of X-ray absorption near-edge structure (XANES) spectra, we employed the full multiple scattering method. The EXAFS spectra, on the other hand, were calculated by the path expansion method. Both XANES and EXAFS calculations included a maximum of 30 SCF cycles to achieve self-consistent potentials. Difference spectra were generated by subtracting the spectrum of the S_0_ geometry from the spectrum of the T_1_ or Q_1_ geometry, thus simulating the experimental pump-on minus pump-off (excited state minus ground state) transient spectra. For the EXAFS spectra, the ABSOLUTE card was used to avoid artifacts caused by normalization. To achieve absolute energy agreement with experiment, the spectra were shifted by −10 eV. The intensities of the calculated spectra were also scaled (by the same amount when on the same vertical scale) to match the experimental intensities.

## Results and Discussion

The complex [FcCc]PF_6_ was synthesized using an optimized synthetic procedure, as described in the Supporting Information (SI) Section S1. The electronic structure of [FcCc]^+^ was calculated with DFT at the B3PW91/6-311+G(d) PCM(acetonitrile) level of theory, which was shown by Wagenknecht and co-workers^17,61^ to reliably capture ferrocene-to-titanocene MMCT. Additional calculations with B3LYP/6-311+G(d) PCM(acetonitrile) and B3LYP/LANL2DZ are presented in Supporting Information Section 2.

**Figure 1:**
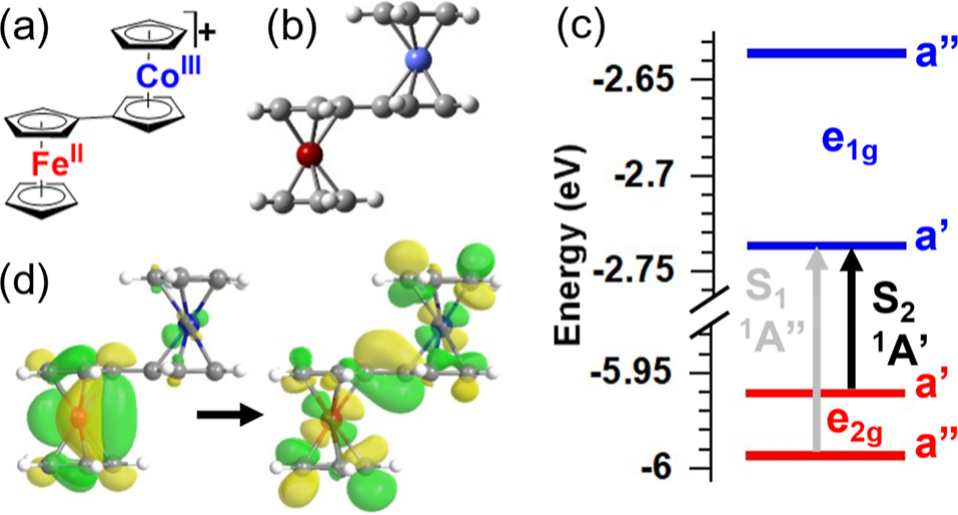
Electronic and geometric structure of [FcCc]^+^. a) Lewis structure and (b) DFT-optimized geometry of the S_0_ state of [FcCc]^+^. (c) Frontier molecular orbital diagram of [FcCc]^+^ showing Fe-centered (red) HOMOs and Co-centered (blue) LUMOs and the highest orbital contributions of the two lowest TD-DFT transitions. (d) Dominant natural transition orbitals of the S_0_ → S_2_ TD-DFT transition.

The geometric and electronic structure of [FcCc]^+^ (Figure 1) is defined by the interaction between the ferrocene donor and cobaltocenium acceptor. The two low-spin d^6^ metallocenes are connected by a single bond, which lowers their point-group symmetries from D_5d_ to C_s_. The highest-occupied molecular orbitals (HOMOs) are the ferrocene e_2g_ orbitals and the lowest-unoccupied molecular orbitals (LUMOs) are the cobaltocenium e_1g_ orbitals (Figure 1c). Coupling between the metallocenes splits these degenerate orbitals into a′ and a″ orbitals. The strong π-conjugation through the bridging fulvalene ligand mixes the unoccupied e_1g_ a′ d_*xz*_ orbitals of Co and Fe, stabilizing the Co a′ LUMO. This orbital is the acceptor orbital for the two lowest TD-DFT transitions, S_0_ → S_1_, and S_0_ → S_2_. These are MMCT transitions that originate from the Fe e_2g_ HOMOs and are either ^1^A′ or ^1^A″ in symmetry. The ^1^A’ S_2_ state is optically bright (*f* = 0.026) with its transition dipole pointing along the Fe–Co axis. The ^1^A″ S_1_ state, on the other hand, is optically dark (*f* = 0.0002) because its transition dipole is necessarily polarized orthogonal to the symmetry plane and Fe–Co axis. The dominant natural transition orbitals (NTOs) for the bright S_0_ → S_2_ MMCT transition are shown in Figure 1d. Mixing between the ^1^MMCT state and ^1^(d–d) state, facilitated by the aforementioned π conjugation of Fe and Co d_*xz*_ orbitals, is evident from the electron density on ferrocene in the final-state/particle NTO.

**Figure 2:**
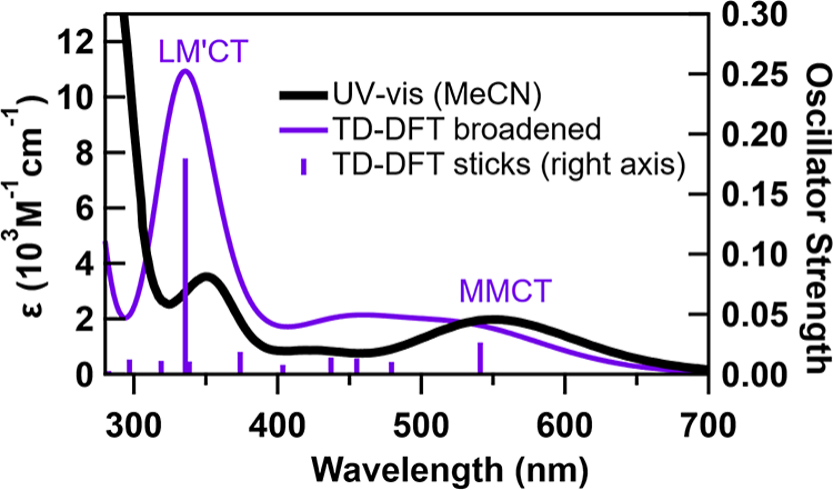
UV–vis spectrum (left axis) of [FcCc]PF_6_ in acetonitrile (MeCN) overlaid with TD-DFT stick spectrum (right axis) and broadened spectrum (left axis) of [FcCc]^+^. Bands are labeled with their assignments made from visual inspection of natural transition orbitals. MMCT = metal-to-metal charge transfer, LM′CT = ligand-to-other-metal charge transfer.

The UV–vis absorption spectrum of [FcCc]PF_6_ in acetonitrile (MeCN) is shown in Figure 2. The calculated TD-DFT spectrum of [FcCc]^+^ is overlaid for comparison (See Supporting Information Section 2 for details). The MMCT band is clearly observed at 550 nm. The other prominent band in the UV–vis spectrum is the ligand-to-other-metal charge transfer (LM′CT) band at 350 nm, which transfers an electron from the ferrocene ligand to cobaltocenium metal. The intensity of this transition is overestimated by TD-DFT. For more details about these and other TD-DFT transitions, see Table S1. In our transient absorption experiments, we pump the MMCT band and monitor the changes in the absorption spectrum that occur during relaxation.

**Figure 3:**
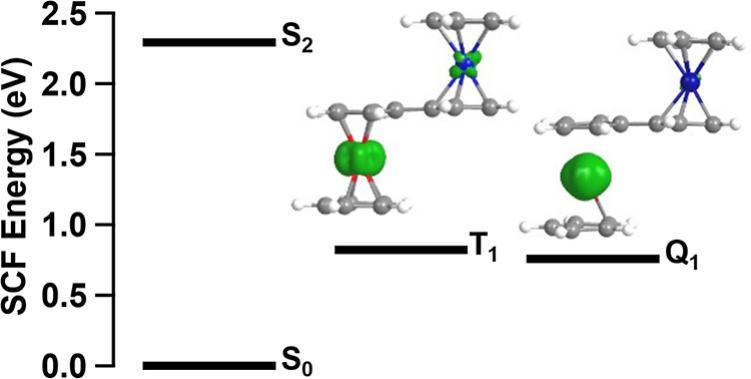
Relative SCF energies of geometry-optimized S_0_, S_2_, T_1_, and Q_1_ states calculated by (TD-)DFT. Spin-density plots of the lowest T_1_ and Q_1_ states are shown (green = alpha spin, yellow = beta spin, 0.02 isovalue).

To gain a sense of the energetic landscape of [FcCc]^+^, we performed an excited-state geometry optimization of the S_2_ MMCT state and ground-state geometry optimizations of the lowest triplet (T_1_) and quintet (Q_1_) states. Figure 3 shows the energies of these states relative to the S_0_ ground state. The T_1_ and Q_1_ states are close in energy, with the Q_1_ state being slightly lower in energy (Figure S4). Single-point energy calculations at geometries interpolated between the T_1_ and Q_1_ minima reveal a barrier of 2.2 kcal/mol between the potential energy surfaces (Figure S5). Spin density plots (Figure 3) of the T_1_ and Q_1_ states show primarily Fe(II) (d–d) character with some ^3^MMCT admixture in the T_1_ state. Both the T_1_ and Q_1_ states, and several of their TD-DFT excited states, lie below the S_2_ state, revealing a manifold of ferrocene-centered ^3^(d–d) and ^5^(d–d) states accessible during MMCT relaxation (Figure S6). As we will show in the next section, these ferrocene-centered, ligand-field excited states play an important role in the photophysics of [FcCc]^+^.

### OTA Spectroscopy

To investigate the relaxation mechanism of [FcCc]PF_6_ after MMCT excitation, we performed a barrage of OTA spectroscopy experiments with distinct probe pulses covering the UV–vis, vis, NIR and SWIR spectral regions. The experiments were performed in MeCN solution with a pump wavelength of 532 nm (UV–vis probe) or 515 nm (vis, NIR, and SWIR probes) to excite the MMCT transition.

**Figure 4:**
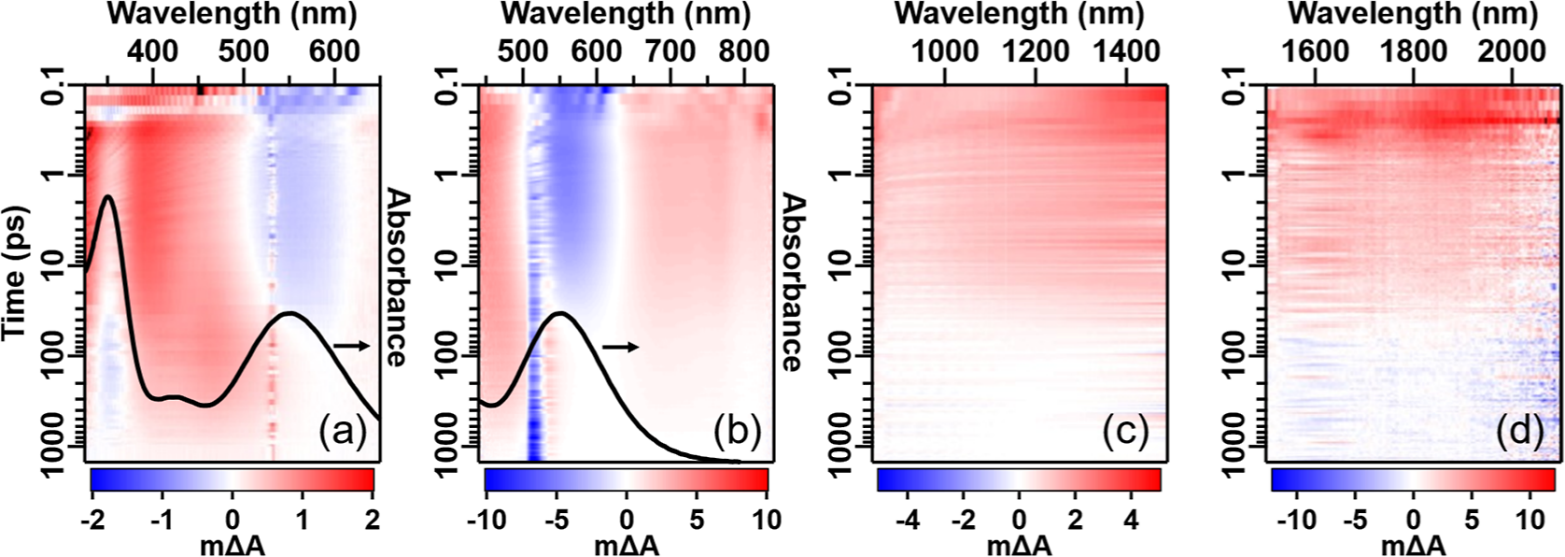
2D maps of OTA data with (a) UV–vis, (b) vis, (c) NIR, and (d) SWIR probes. Pixels that saturate the color scale are colored black. The UV–vis (a) and vis (b) 2D maps show anomalous signal due to pump scatter at 532 and 515 nm, respectively, and due to coherent artifacts during pump–probe overlap at time delays of <0.3 and <0.2 ps, respectively. The static, ground-state absorption spectrum is shown as a black line plotted on the right axes of (a,b).

The OTA data are presented in Figure 4 as 2D maps that show the transient absorption signal at each probe wavelength and time delay. The OTA signal is a difference in absorbance corresponding to “pump-on” minus “pump-off”. The signal is presented on a color scale where positive excited-state absorption (ESA) signals are red and negative ground-state bleach (GSB) signals are blue. In OTA, ESA signals occur at wavelengths where the excited state absorbs more strongly than the ground state, and GSB signals occur at wavelengths where the ground state absorbs more strongly than the excited state.

The transient spectra in Figure 4 show large changes as a function of time. At early times (<10 ps), there is a GSB at the 550 nm MMCT band (Figure 4a,b). In addition, a broad ESA is observed in the NIR (Figure 4c) and SWIR (Figure 4d) regions. In the UV–vis (Figure 4a) region, there is an ESA from 320 to 500 nm, with a maximum at 400 nm. This ESA outcompetes the LM′CT bleach, which appears as a dip in the ESA at 350 nm. At later times (>100 ps), the spectra are drastically different. For one, the ESA in the NIR and SWIR regions has completely decayed. Furthermore, the ESA in the UV–vis region has red-shifted to a maximum at 470 nm, which as a result exposes the negative LM′CT GSB signal at 350 nm and cancels out the MMCT GSB at 550 nm. The large qualitative changes in transient spectra suggest that we are probing at least two distinct electronic states. To extract the lifetimes of these states, we performed kinetic fitting.

**Figure 5:**
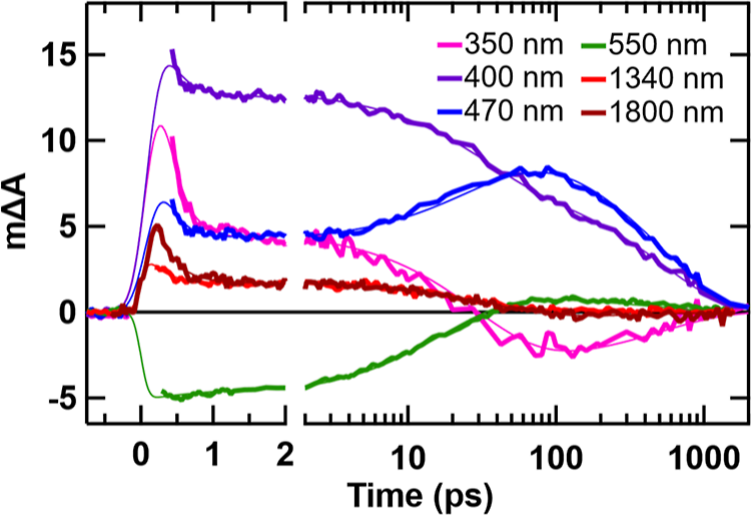
Kinetic traces (thick lines) and global fits (thin lines) at select probe wavelengths from the UV–vis (350, 400, 470 nm), vis (550 nm), NIR (1340 nm), and SWIR (1800 nm) OTA experiments of [FcCc]PF_6_ in MeCN. Each experiment was separately fit to a 3-component sequential model. The UV–vis data and SWIR data have been scaled factors of 10 and 0.6, respectively, to be on the same vertical axis as the vis and NIR data; see Supporting Information for details.

Kinetic traces and global fitting results of the OTA data are shown at select probe wavelengths in Figure 5. To fit the data, we performed target analysis assuming a sequential kinetic model with 3 components. Each OTA experiment was separately fit to the same model (time constants were not linked between experiments). For more details, see Supporting Information Section 3.2. The time constants obtained from the global fits are tabulated in Table 1. Averaging the time constants from each fit, we obtain τ_1_ = 0.24 ± 0.07 ps, τ_2_ = 27.8 ± 8.0 ps, and τ_3_ = 483 ± 59 ps.

**Table 1:** Time Constants Obtained from Global Fits of OTA Data to a 3-Component Sequential Kinetic Model

Probe	UV–vis^b^	vis^b^	NIR^b^	SWIR^b^	Average^d^
IRF/ps^a^	0.2 (held)	0.2^c^	0.2^c^	0.28 ± 0.02	
τ_1_/ps	0.15 ± 0.01	0.33 ± 0.01	0.25 ± 0.01	0.23 ± 0.01	0.24 ± 0.07
τ_2_/ps	31.5 ± 0.9	17.6 ± 0.1	26.0 ± 0.5	36.3 ± 1.8	27.9 ± 8.0
τ_3_/ps	525 ± 10	442 ± 3	412^c^	500^c^	483 ± 59

a fwhm of the Gaussian instrument response function (IRF).

b Best fit plus or minus the standard deviation calculated from the residuals of the fit.

c Held constant.

d Average plus or minus standard deviation of the parameters from each fit. Parameters that were held constant were not included in the average.

**Figure 6:**
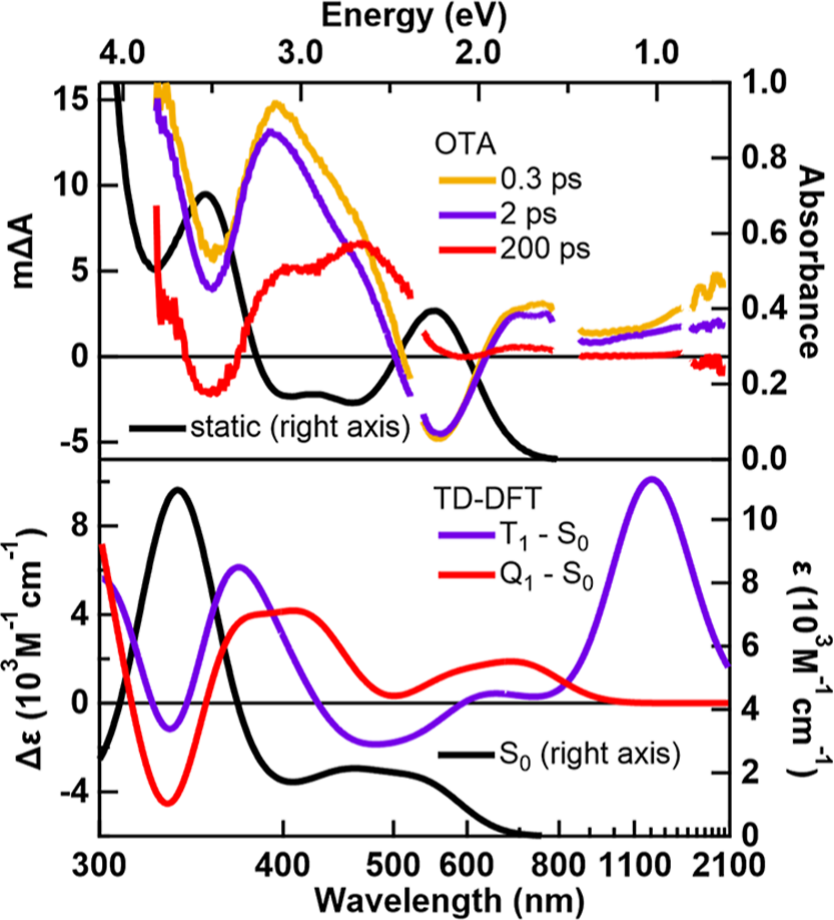
Comparison of OTA and TD-DFT spectra. Top panel: experimental static (right axis) and transient (left axis) spectra at 0.3, 2 and 200 ps. The UV–vis and SWIR OTA data have been scaled to be on the same vertical scale as the vis and NIR OTA data; see Supporting Information for details. Bottom panel: broadened TD-DFT spectrum of the S_0_ state (right axis) and difference spectra of the T_1_ and Q_1_ states with the S_0_ state (left axis).

Figure 6 (top panel) shows spectral slices stitched together from each OTA experiment. The signal amplitudes were appropriately scaled to place the data from different experiments on the same vertical scale (see Supporting Information Section 3.3 for more details). The time delays of the spectral slices were chosen to be representative of the three species modeled in the global fits. For example, at 2 ps, essentially all the excited-state population is in the intermediate state with lifetime τ_2_ = ∼30 ps. Meanwhile at 200 ps, the long-lived state with lifetime τ_3_ = ∼500 ps is the only excited state with appreciable population. The short-lived state with lifetime τ_1_ = ∼0.2 ps, on the other hand, has a lifetime comparable to the instrument response function (IRF), and therefore only dominates during pump–probe overlap. Unfortunately, the UV–vis and vis OTA signals are obscured by coherent artifacts during pump–probe overlap, so the earliest spectrum we resolve is at a time delay of 0.3 ps. At this time delay, most of the population has already decayed to the intermediate state, but nevertheless, the spectral changes associated with τ_1_ can be seen by comparing the 0.3 and 2 ps traces. In the following, we focus on assigning the intermediate state with lifetime τ_2_ = ∼30 ps and the long-lived state with lifetime τ_3_ = ∼500 ps.

To assign the species observed with OTA, we simulated the spectra with TD-DFT (Figure 6 bottom panel). Within linear-response TD-DFT, we are limited to simulating the ground states of a given spin multiplicity, such as the S_0_, T_1_, and Q_1_ states. As a result, the initially excited ^1^MMCT state, S_2_, is inaccessible with this method, and simulating it would require quadratic-response TD-DFT.^62^ Nevertheless, difference spectra of the (d–d) states were calculated by subtracting the spectrum of the S_0_ ground state from the spectrum of the T_1_ or Q_1_ state. The raw T_1_ and Q_1_ TD-DFT spectra before taking the difference are shown in Figures S7 and S8. We find that the TD-DFT spectra qualitatively reproduce the experimental spectra across the broad spectral range probed. A qualitative agreement is seen between the 2 ps transient and the T_1_-S_0_ difference spectrum, and between the 200 ps transient and the Q_1_-S_0_ difference spectrum. Specifically, the T_1_-S_0_ spectrum features an intense ESA caused by the T_1_ → T_5_ Fe-e_1g_-to-Co-e_1g_ MMCT transition at 1185.7 nm (Table S3) which reproduces the NIR/SWIR ESA in the 2 ps transient. Although the experimental NIR/SWIR intensity is less than predicted by TD-DFT, the discrepancy may be caused by TD-DFT overpredicting the oscillator strength of the T_1_ → T_5_ transition. Another example of TD-DFT overpredicting the oscillator strength is given by the S_0_ → S_18_ LM′CT transition observed at 350 nm in the static UV–vis spectrum (Figure 2). This transition, like the T_1_ → T_5_ transition, is a CT transition of A′ symmetry polarized along the Fe–Co axis, and the calculated extinction coefficient is 3x larger than observed in the experimental UV–vis spectrum. Therefore, the TD-DFT of the T_1_ state is consistent with the NIR/SWIR spectrum at 2 ps. The experimental transient at 200 ps, on the other hand, shows no OTA signal in the NIR/SWIR, and is thus not consistent with the T_1_ state. Instead, the long-lived transient signal is better reproduced by the Q_1_-S_0_ spectrum, which does not show an ESA in this region.

In the UV–vis region, the experimental transient spectra show a GSB of the 550 nm MMCT band at 2 ps, and a GSB of the 350 nm LM′CT band at 200 ps. In the 2 ps transient, the LM′CT bleach is outcompeted by an ESA, resulting in a dip in the ESA at 350 nm and a maximum at 400 nm. The T_1_-S_0_ spectrum reproduces these features, with the T_1_ → T_27_ transition at 355.8 nm (Table S3) competing with the S_0_ → S_18_ LM′CT bleach at 335.7 nm. In the B3PW91 T_1_-S_0_ difference spectrum, unlike experiment, the S_0_ → S_18_ LM′CT transition narrowly outcompetes the T_1_ → T_27_, resulting in a negative GSB signal. However, calculations with the B3LYP functional (Figure S9) accurately reproduced the positive sign of the signal observed at the 350 nm LM′CT band in the experimental 2 ps transient. Compared with the 2 ps transient, the 200 ps transient shows a drop in intensity of the 400 nm ESA, revealing the LM′CT GSB, and appearance of a red-shifted ESA at 470 nm, which cancels out the 550 nm MMCT GSB. The Q_1_-S_0_ spectrum reproduces all these features, with the Q_1_ → Q_28_ transition at 357.3 nm (Table S4) being weaker (*f* = 0.010) than the T_1_ → T_27_ transition (*f* = 0.156) at 355.8 nm, resulting in a more negative S_0_ → S_18_ LM′CT GSB feature, and the Q_1_ → Q_18_ transition 421.2 nm appearing as a red-shifted ESA that helps cancel out the MMCT GSB.

Based on the qualitative agreement between TD-DFT and OTA, we assign the τ_2_ = ∼30 ps state to the T_1_ state and the τ_3_ = ∼500 ps state to the Q_1_ state. These states are both predominantly Fe(II) (d–d) states, meaning the Fe(III)Co(II) MMCT state has already undergone charge recombination by the time they form. Therefore, τ_1_ = ∼0.2 ps must correspond to intersystem crossing (ISC) and back electron transfer (BET) or ultrafast cooling of the hot ^3^(d–d) state. In the UV–vis and vis regions, the 0.3 and 2 ps transients look similar but differ in the position, width, and intensity of some bands. This would suggest that these spectra are of the same electronic state and τ_1_ corresponds to cooling of the hot ^3^(d–d) state. Indeed, BET following MMCT is known to occur on a ∼100 fs time scale^63,64^ and populate nonequilibrium distributions of vibrational^65,66^ and solvent^67^ coordinates. However, τ_1_ is comparable to the IRF and coherent artifacts obscure the transient signal during pump–probe overlap, so it is unclear how much the spectra differ at time delays <0.3 ps. On the other hand, the NIR and SWIR experiments do not suffer from large coherent artifacts, and we are able to probe at time delays shorter than 0.3 ps (Figure S34). In the NIR/SWIR region, we detect distinct spectral changes on the femtosecond time scale. As shown in Figure 6, between 0.3 and 2 ps, the NIR/SWIR ESA undergoes a significant drop in intensity, blue shift, and change in spectral shape. The distinct spectra at early times in the NIR/SWIR may be evidence for the MMCT state, but the featureless ESAs and lack of a TD-DFT model of the MMCT state for comparison limit the information content of the spectra. We therefore tentatively assign τ_1_ to cooling of the hot ^3^(d–d) state, but note that solvent relaxation in the MMCT state, BET, and solvent relaxation in the product state can occur on competing time scales^68^ and may not be separable with exponential kinetics.

**Figure 7:**
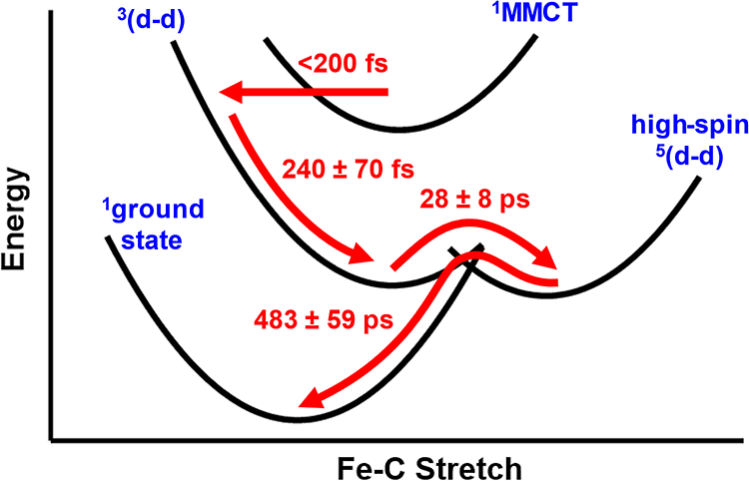
Proposed mechanism of MMCT relaxation in [FcCc]PF_6_. Time constants are averages of those obtained by target analysis of OTA data.

Based on OTA and TD-DFT, we put forth the following mechanism of MMCT relaxation in [FcCc]PF_6_ (Figure 7): in less than ∼200 fs, the ^1^MMCT state undergoes ISC and BET to a hot ^3^(d–d) state which undergoes cooling on a ∼0.2 ps time scale. The ^3^(d–d) state, in turn, undergoes another ISC on a ∼30 ps time scale to form the high-spin ^5^(d–d) state. The high-spin ^5^(d–d) state then undergoes ISC/ground-state recovery on a ∼500 ps time scale.

Despite the ubiquity of ferrocene donors in D–A molecules, there are few examples of the ferrocene-centered high-spin state in the literature. Therefore, to put this assignment on firmer ground, we turn to a technique that is both element specific and spin-state sensitive to verify that the energy deposited by the pump pulse is localized to the ferrocene donor in its high-spin excited state.

### X-ray Transient Absorption Spectroscopy

To confirm the ferrocene-centered high-spin character of the long-lived excited state, we performed hard X-ray transient absorption (XTA) spectroscopy experiments at beamline 11ID-D of the Advanced Photon Source.^56^ A liquid jet sample of [FcCc]PF_6_ in MeCN was excited with ∼120 fs, 515 nm laser pump pulses and probed with ∼80 ps pulses of hard X-ray synchrotron radiation. The ∼80 ps time resolution of the XTA experiment is not sufficient to resolve the lifetimes of the τ_1_ = ∼0.2 ps and τ_2_ = ∼30 ps states identified by OTA. Still, to ensure that all of the τ_2_ = ∼30 ps state has decayed, we chose to record spectral traces at a time delay of 150 ps, at which nearly all the population is calculated to be in the τ_3_ = ∼500 ps state. K-edge X-ray absorption spectra can be divided into three regions: the pre-edge region, the XANES region, and the EXAFS region. The former is dominated by weak, quadrupole-allowed 1s → 3d transitions that yield information on electronic structure and 3d orbital occupation. The latter two are dominated by dipole-allowed 1s → np transitions that contain structural information encoded by the scattering of the excited photoelectron off the atoms in the coordination sphere(s). The electronic and structural sensitivity of K-edge XTA is ideal for probing the changes in d-orbital occupation and bond-length expansion that accompanies Fe(II) photoinduced spin crossover.^69,70^ We measured XTA at both the Fe and Co K edges to leverage the elemental specificity of the technique to determine whether energy and charge are localized to the ferrocene donor or cobaltocenium acceptor.

**Figure 8:**
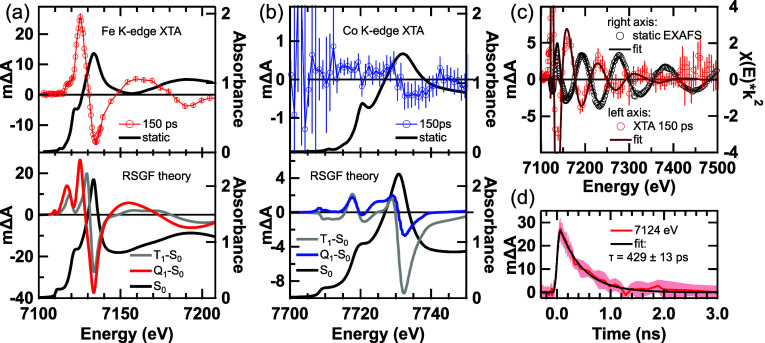
X-ray transient absorption spectroscopy of [FcCc]PF_6_ at the (a) Fe K-edge and (b) Co K-edge XANES regions. Transient/difference spectra are plotted on the left axes and static spectra are plotted on the right axes. The error bars correspond to a ±99.9% confidence interval. Top panels of (a,b) show experimental data and bottom panel shows RSGF theory calculations. The theoretical spectra have been horizontally shifted by −9.4 eV to align with experiment and vertically scaled by a factor of 0.03 corresponding to a 3% excitation fraction. (c) Fe K-edge EXAFS. Right axis: static, background-subtracted EXAFS data (black dots) and fit (black line) with a *k*-weight of 2. Left axis: XTA signal (red dots, error bars are ±99.9% confidence interval) and model difference EXAFS fit (dark red line). (d) kinetic trace measured at the maximum of the Fe-edge XANES ESA, fit to a single exponential decay convoluted with a Gaussian instrument response function. Error bars are ± σ (standard deviation). Experimental conditions: 5 mM [FcCc]PF_6_ in MeCN, 700 μm cylindrical liquid jet, 515 nm, 3 kHz, 20 mJ/cm^2^ pump.

The Fe and Co K-edge XANES spectra are shown in Figure 8a,b, respectively. The static Fe and Co spectra of [FcCc]PF_6_ look very similar due to ferrocene and cobaltocenium both being low-spin, d^6^ metallocenes. Both feature a single, weak, quadrupole-allowed 1s → 3d pre-edge transition to the vacant e_1g_ orbitals. In the XANES region, there is a shoulder on the rising absorption edge, an intense “white-line” peak, and oscillations above the absorption edge. Excitation with the 515 nm laser leads to large transient changes in the Fe K-edge XANES region. A strong bleach of the white line at 7134 eV is accompanied by an ESA at 7125 eV, producing a derivative-like feature indicative of a red shift of the absorption edge in the excited state. Such edge shifts are typical of increases in metal spin state.^69,70^ On the rising edge of the ESA, we observe a shoulder feature at 7117 eV. Above the edge, a pronounced oscillatory feature is observed. At the pre-edge, an ESA signal is observed, suggesting an increase in 3d hole count or increased 3d–4p mixing (due to changes in symmetry) in the excited state. The transient signal at the Co K edge, on the other hand, is about 50× weaker for the same laser excitation fluence. The small Co-edge signal magnitude precluded resolving transient features in the pre-edge or EXAFS regions, but with extended averaging we resolved a weak Co K-edge XANES signal within a 99.9% confidence interval, indicating a bleach of the white line and red-shifted ESA. The large changes observed at the Fe K-edge and rather small changes at the Co K-edge support a ligand-field excited state centered at the Fe atom. To gain more insight into the identity of the state, we performed theoretical calculations to model the spectra.

K-edge XANES is dominated by multiple scattering events due to the long wavelength of the photoelectron near the ionization threshold, *E*_0_. To simulate the spectra, we performed full-multiple scattering calculations using real-space Green’s function (RSGF) theory within the muffin-tin approximation, as implemented in FEFF10^59,60^ (see Supporting Information Section S5 for details). As input geometries, we utilized the DFT geometry-optimized structures of the S_0_, T_1_, and Q_1_ states of [FcCc]^+^, whose average Fe–C bond lengths vary between 2.05, 2.15, and 2.34 Å, respectively. The increase in bond length leads to a red shift of the absorption edge, resulting in derivative-like features in the difference spectra constructed by subtracting the S_0_ ground-state spectrum from the Q_1_ or T_1_ spectrum, as seen in Figure 8. The theoretical difference spectra in Figure 8 have been scaled according to a 3% excitation fraction, which is the optimal excitation fraction found in our EXAFS analysis (vide infra) and puts the theoretical Fe K-edge difference spectra at a similar magnitude to the experimental Fe K-edge XTA.

Overall, the shape of the simulated transient spectrum for the Q_1_ state matches the experimental spectrum better than for the T_1_. Quantitatively, the spacing between the bleach and red-shifted ESA in the experimental transient is 9.6 eV. The peaks of the Q_1_-S_0_ derivative-like feature have a spacing of 8.2 eV, which better reproduces the experimental transient than the 4.5 eV spacing observed in the T_1_-S_0_ spectrum. At the Co center, on the other hand, the DFT geometry-optimized Co–C bond lengths are largely insensitive to the spin multiplicity due to the Fe-centered character of the T_1_ and Q_1_ states. In the S_0_ and Q_1_ states, the average Co–C bond lengths are both 2.04 Å, whereas a modest increase to 2.06 Å in the T_1_ state is attributed to ^3^(d–d)/^3^MMCT mixing, as suggested by the spin density on the Co center in the T_1_ state (Figure 3). The negligible change in Co–C bond length in the Q_1_ state leads to a small transient signal at the Co K-edge. In contrast, the slight bond-length increase in the T_1_ state leads to a much larger transient signal than we observe experimentally. Overall, the small XTA signal magnitude at the Co K-edge rules out any states that are Co-centered or have strong Fe–Co interactions, and the magnitude of red shift at the Fe K edge is more consistent with that calculated for the high-spin state.

To verify that we are probing the same transient species in our OTA and XTA experiments, we measured a kinetic trace at the ESA maximum of the Fe K-edge XANES (Figure 8d). The data were fit to a single exponential decay convoluted with an ∼80 ps IRF (see Supporting Information Section S4.2), yielding a time constant of 429 ± 13 ps in close agreement with τ_3_ from the OTA experiment. Our OTA and XTA measurements thus both provide evidence for a long-lived state with a ∼500 ps lifetime, and our TD-DFT and RSGF theory calculations both point to this state being the high-spin state of the ferrocene donor. To further test this assignment, we modeled the Fe pre-edge region with TD-DFT calculations (Supporting Information Section S4.3). The pre-edge is a sensitive probe of electronic structure and symmetry. However, the calculated pre-edge spectra of the T_1_ and Q_1_ states are similar, and both reasonably reproduce the experimental pre-edge ESA. Therefore, the pre-edge XTA signal is consistent with a ligand-field excited state but is ambiguous as to whether it arises from the ^3^(d–d) state or ^5^(d–d) state. For that reason, we turn to a geometric structural probe of the excited state, the EXAFS region, to distinguish between these states.

The EXAFS region of K-edge X-ray absorption spectra encodes quantitative structural information on the coordination sphere(s) of the absorbing atom. Above the absorption edge/ionization threshold, photoelectrons are excited with an energy-dependent wavevector *k* and wavelength λ = 2π/*k* and scatter off nearby atoms. Wave interference of the emitted and backscattered photoelectrons at the position of the absorbing atom leads to interference fringes in the absorption spectrum whose frequencies are determined by the scattering path lengths (and thus bond lengths) of the material. The static and transient EXAFS spectra in energy space of [FcCc]PF_6_ at 150 ps are shown in Figure 8c. The static spectrum has been background-subtracted and multiplied by *k*^2^ (*k*-weight of 2) to accentuate the oscillations at high photon energies. To extract quantitative structural information, we fit the static spectrum to the EXAFS equation (Equation S5) with a structural model based on the DFT geometry-optimized structure of the S_0_ state, including 18 single- and multiple-scattering pathways involving the C atoms of the ligands.^71^ The structural model is symmetric, with the first Fe–C single scattering pathway having a degeneracy of 10. To avoid overfitting the data, we included a single structural parameter that simply scales the distance of each scattering path. For more details, see Supporting Information Section S4.4. This procedure yielded an Fe–C bond length of 2.039 ± 0.007 Å, close to the value of 2.05 Å from DFT calculations.

To determine the structural changes in the excited state, we followed the method of Gawelda et al.^72^ of fitting the transient spectrum to a model difference spectrum in energy space. Our structural model for the excited state starts from the fitted ground-state structure and symmetrically expands the Fe–C bond lengths while keeping all C–C bond lengths constant. We calculated the excited-state spectra for various values of Fe–C bond length increase, Δ*R*, and excited-state shift in ionization threshold, Δ*E*_0_. Then, excited-state minus ground-state difference spectra are calculated in energy space for various excitation fractions, *f*, and compared to the experimental XTA spectrum. The goodness of fit is quantified by calculating the reduced chi-squared (χ_r_^2^), which we define in Equation S6. We calculated χ_r_^2^ surfaces in the parameter space to determine which set of parameters minimizes χ_r_^2^ (see Supporting Information Section 4.5 for details). We found the minimum at *f* = 3% and Δ*E*_0_ = −0.8 eV with an Fe–C bond-length increase of Δ*R* = 0.25 ± 0.1 Å (Figure S59). The error in Δ*R* represents a confidence region of 68% (±1σ) estimated as the change in Δ*R* required to increase χ_r_^2^ by 1 from its minimal value.^73^ This increase corresponds to an Fe–C bond length of 2.29 ± 0.1 Å in the excited state. Figure S61 compares the fitted values of Δ*R* to the predictions from DFT. In [FcCc]^+^, the increase in average Fe–C bond length from the S_0_ to Q_1_ state is Δ*R* = 0.29 Å, which falls within the ±1σ error of our fitted value of Δ*R* = 0.25 ± 0.1 Å. For comparison, the Fe–N bond-length expansion in the high-spin state of [Fe(bpy)_3_]^2+^ is 0.203 ± 0.008 Å.^72^ The T_1_ state of [FcCc]^+^, on the other hand, has a smaller Fe–C bond length expansion of Δ*R* = 0.10 Å. As shown by Figure S59, fits with Δ*R* = 0.10 Å consistently give significantly higher values of χ_r_^2^. Therefore, the transient Fe K-edge EXAFS data show that the long-lived excited state of [FcCc]PF_6_ cannot be described as a ^3^(d–d) state and instead shows a bond-length expansion consistent with the ferrocene-centered high-spin state.

### Comparison to Other Fe(II) Complexes

The unique ultrafast behavior of [FcCc]PF_6_ warrants further discussion. Commonly studied mixed valence compounds such as [(NC)_5_Fe^II^CNRu^III^(NH_3_)_5_]^+67^ and Fe(II)Co(III) Prussian Blue analogues (PBAs)^74−76^ are based on the ferrocyanide moiety, which has a large ligand-field splitting of 10Dq = 3.9 eV^77,78^ that destabilizes the Fe(II) (d–d) states, leading to vastly different MMCT relaxation dynamics. In comparison, ferrocene has a relatively small ligand field splitting of Δ_2_ = 2.73 eV^79^ eV, which presents a manifold of Fe(II) (d–d) states that are energetically accessible in [FcCc]PF_6_. Similarly, Fe(II) spin crossover complexes such as [Fe^II^(H_2_B(pz)_2_)_2_phen] (phen = 1,10-phenanthroline, H_2_B(pz)_2_ = bispyrazolylborate), which has a ligand field strength of 10Dq = 2.26 eV,^80^ possess Fe(II) (d–d) manifolds that are known to quench metal-to-ligand CT (MCLT) excited states. The^1,3^ MLCT → ^3^(d–d)→ ^5^(d–d) mechanism of photoinduced spin crossover is nearly identical to MMCT relaxation in [FcCc]PF_6_, but the time scales involved are drastically different. Decay of the intermediate ^3^(d–d) state in Fe(II) spin crossover complexes is ballistic,^81^ occurring in only 39 fs for [Fe(phen)_3_]^2+^.^82^ In contrast, the intermediate ^3^(d–d) state in [FcCc]PF_6_ is 3 orders of magnitude longer, indicating that this state thermalizes before ISC to the ^5^(d–d) state. This suggests large changes in the potential energy landscape of Fe(II) ions in O_h_ versus D_5d_ ligand fields. Indeed, we calculated a ∼ 2.2 kcal/mol barrier between the T_1_ and Q_1_ states of [FcCc]^+^ from DFT, which is in line with estimates based on the ∼30 ps time constant from OTA using transition state theory (Supporting Information Section S6). This finding could inspire novel ligand-design strategies to tune the ligand-field manifold of earth-abundant Fe(II) photosensitizers.^83,84^ In OTA studies of similar ferrocene–acceptor complexes with MMCT or MLCT transitions, transients with ∼30 ps lifetimes have been previously observed but assigned as singlet states.^15,17^ In bis(ethynylferrocenyl)titanocene, complexation of CuBr by the alkynyl bridge led to a long-lived 60 ns excited state, which was assigned as a ^3^(d–d) state.^17^ Ultrafast charge recombination to ferrocene-centered excited states should be studied in more detail to resolve discrepancies in the literature. Given the important roles of spin-vibronic mechanisms^85^ in ultrafast ISC and vibrational tunneling^65,86,87^ in ultrafast BET, future studies should use femtosecond X-ray techniques^81,88,89^ and electronic-vibrational spectroscopies^90,91^ to examine the subpicosecond dynamics of ferrocene-containing D–A molecules. To this end, the unique ISC/BET dynamics of [FcCc]PF_6_ and its high stability under laser and X-ray beams make this molecule an ideal system for examining the interplay of electronic, nuclear, and spin degrees of freedom.

## Conclusions

In this contribution, we characterized the excited-state dynamics of a bimetallic D–A molecule, [FcCc]PF_6_. This Fe(II)Co(III) mixed-valence complex has low-lying, ferrocene-centered, (d–d) excited states that lead to quenching of the MMCT state. With a combination of ultrafast OTA from the UV to SWIR, and XTA at the Fe and Co K edges, we discovered that, following MMCT excitation, this complex undergoes ultrafast ISC and BET to an Fe(II) ^3^(d–d) state, which then evolves to an Fe(II) ^5^(d–d) state, which is the high-spin state of the ferrocene donor (Figure 7).

Our results add to the known experimental demonstrations of the high-spin state, which so far has mostly been studied threoretically.^31−36^ To the best of our knowledge, the only other experimental reports of high-spin ferrocene come from Mössbauer spectroscopy experiments on Fe(C_5_H_5_)_2_·CCl_4_ CT complexes embedded in low-temperature films under continuous photoirradiation^37,38^ and as a reactive intermediate adsorbed in the pores of a magnetic metal–organic framework.^30^ Our results show that this state is also accessible on the ultrafast time scale in D–A molecules in solution at room temperature. This has major implications for time-resolved spectroscopic studies of ferrocene-containing D–A molecules. In past spectroscopic studies, the ^3^(d–d) state of ferrocene donors has been observed,^2,8,29^ but mention of the high-spin ^5^(d–d) is missing from the discussions. In many Fe(II) chromophores, the ^5^(d–d) state rapidly quenches photoinduced MLCT states, leading to excited-state lifetimes that are too short for productive photochemistry. Our identification of an accessible high-spin state in [FcCc]PF_6_ suggests that such deactivation pathways may be unexpected loss mechanisms in D–A molecules containing ferrocene.

Detection of the ferrocene high-spin state in D–A molecules requires a suitable spectroscopic probe. In D–A molecules with optically bright dyes such as perylene diimide (PDI)^9^ or dipyrrometheneboron difluoride (BODIPY)^2^ as acceptors, OTA will be mostly insensitive to the electronic structure of the ferrocene donor. Alternatively, DFT calculations suggest that vibrational spectroscopy is sensitive to metallocene spin state.^33,35^ Here, we show that Fe K-edge XTA is an ideal technique for probing the high-spin state of ferrocene donors. The element specificity of the technique can be used to determine whether the excitation energy is localized to the ferrocene or the acceptor unit, and the oxidation-state and spin-state specificity can help distinguish between CT states and ligand-field states. This technique and other complementary core-level spectroscopies will provide useful probes of the high-spin state in other ferrocene-containing compounds and help shed light on the previously neglected state. With further observation and controlled studies, the chemical effects that promote or suppress high-spin state formation in ferrocene electron donors could be identified. Such characterization is important for designing D–A molecules with long-lived CT states suitable for applications in photocatalysis and photovoltaics.

## Supporting Information Available

Log files of all DFT calculations (ZIP)
Synthetic protocol and 1H NMR spectrum of [FcCc]PF_6_, details of DFT calculations including choice of functional and basis set, calculation of optimized singlet/triplet/quintet geometries and simulated TD-DFT spectra, fluence dependences of OTA and XTA signals, detailed spectra and target analysis of OTA data, processing and statistical analysis of XTA data, details of EXAFS fitting, details of real-space Green’s function theory calculations (PDF)

## Abbreviations

[FcCc]PF_6_: ferrocenyl cobaltocenium hexafluorophosphate
D–A: donor–acceptor
CT: charge transfer
MMCT: metal-to-metal charge transfer
LM′CT: ligand-to-other-metal charge transfer
LMCT: ligand-to-metal charge transfer
ISC: intersystem crossing
BET: back electron transfer
TD-DFT: time-dependent density functional theory
OTA: optical transient absorption spectroscopy
UV: ultraviolet
vis: visible
NIR: near-infrared
SWIR: short-wave infrared
XTA: X-ray transient absorption spectroscopy
XANES: X-ray absorption near edge structure
EXAFS: extended X-ray absorption fine structure
RSGF: real-space Green’s function
