# Supplementary material for: High-Spin State of a Ferrocene Electron Donor Revealed by Optical and X-ray Transient Absorption Spectroscopy
Source: J Am Chem Soc. 2024 Jul 25;146(31):21651–63. doi: 10.1021/jacs.4c05646 (PMC11311227; doi:10.1021/jacs.4c05646)
Supplement: Supplementary file 2 — ja4c05646_si_002.pdf [file ja4c05646_si_002.pdf]

Supplementary Information

for

High-Spin State of a Ferrocene Electron Donor Revealed by Optical and X-ray Transient Absorption Spectroscopy

John H. Burke,*[a] Dae Young Bae,[a] Rachel F. Wallick,[a] Conner P. Dykstra,[a] Thomas C. Rossi,[b] Laura E. Smith,[a] Clare A. Leahy,[a] Richard D. Schaller,[c,d] Liviu M. Mirica[a], Josh Vura-Weis*[a], Renske M. van der Veen*[a,b,e]

a) *Department of Chemistry, University of Illinois at Urbana-Champaign, Urbana, Illinois, 61801, USA.*
b) *Department of Atomic-Scale Dynamics in Light-Energy Conversion, Helmholtz-Zentrum Berlin für Materialien und Energie, Berlin, 14109, Germany.*
c) *Department of Chemistry, Northwestern University, Evanston, IL, 60208, USA.*
d) *Center for Nanoscale Materials, Argonne National Laboratory, Lemont, IL, 60439, USA.*
e) *Institute of Optics and Atomic Physics, Technical University of Berlin, 10623, Berlin, Germany*

Contents

1 Synthetic details 7
2 Density Functional Theory 10
2.1 Choice of Model Chemistry 11
3 Optical transient absorption spectroscopy 24
3.1 General Considerations 24
3.2 Target Analysis 28
3.3 Comparison of Signal Amplitudes 48
4 X-ray Transient Absorption Spectroscopy 52
4.1 Data Processing 56
4.2 Kinetic Fitting 65
4.3 Pre-Edge Region 66
4.4 EXAFS Analysis 67
4.4.1 Artemis Log file of static (ground state) EXAFS fit. 72
4.5 Excited-State EXAFS Fitting 77
4.5.2 Parameters used in excited-state EXAFS fitting. 85
5 Real-Space Green's Function Theory 89
5.1 Example FEFF10 input file for calculating XANES spectrum: 90

5.2 Example FEFF10 input file for calculating EXAFS spectrum: 93

6 Estimation of Activation Energy of Triplet-Quintet Intersystem Crossing 98

7 References 100

Figure S1: 1H NMR spectrum of ferrocenylcobaltocenium-hexafluorophosphate at 23 °C (CD3CN). 9

Figure S2: Comparison of TD-DFT spectra of $[FcCc]^+$ with the experimental UV-vis spectrum of $[FcCc]PF_6$ in acetonitrile. TD-DFT oscillator strength sticks (right axis) have been broadened with gaussians lineshapes of $\sigma = 0.3$ eV. 12

Figure S3: UV-vis spectrum (left axis) of $[FcCc]PF_6$ in acetonitrile (MeCN) overlaid with TD-DFT stick spectrum (right axis) of $[FcCc]^+$ calculated at B3PW91/6-311+G(d) PCM(acetonitrile). 13

Figure S4: Relative self-consistent field (SCF) energies and Gibbs free energies (at 298 K and 1 atm) of the lowest singlet (S0), triplet (T1) and quintet (Q1) states of $[FcCc]^+$ at two different levels of theory. Both calculations include PCM(acetonitrile) implicit solvation. The Gibbs free energies of the triplet and quintet states contain contributions from the electronic entropy due to spin degeneracy. 15

Figure S5: Energy barrier between T1 and Q1 states. Single point energy calculations were performed at geometries interpolated between the optimized T1 and Q1 states. Relative SCF energy is plotted here as a function of geometry interpolation point. B3PW91/6-311+G(d) PCM(acetonitrile). 16

Figure S6: TD-DFT vertical transition energies of the S0, T1, and Q1 states, offset by their relative SCF energy. Assignments of states are given based on visual inspection of natural transition orbitals. B3PW91/6-311+G(d) PCM(acetonitrile) 17

Figure S7: Broadened TD-DFT spectra (traces, left axis) and oscillator strengths (sticks, right axis) of the lowest singlet (S0), triplet (T1), and quintet (Q1) states of $[FcCc]^+$ calculated with B3PW91/6-311+G(d) PCM(acetonitrile) and gaussian broadening of linewidth $\sigma = 0.3$ eV. 21

Figure S8: Broadened TD-DFT spectra (traces, left axis) and oscillator strengths (sticks, right axis) of the lowest singlet (S0), triplet (T1), and quintet (Q1) states of $[FcCc]^+$ calculated with B3LYP/6-311+G(d) PCM(acetonitrile) and gaussian broadening of linewidth $\sigma = 0.3$ eV. 22

Figure S9: Comparison of OTA and TD-DFT spectra. Top panel: experimental static (right axis) and transient (left axis) spectra at 0.3 ps, 2 ps and 200 ps. The UV-vis and SWIR OTA data have been scaled to be on the same vertical scale as the vis and NIR OTA data; see text for details. Bottom panel: broadened TD-DFT spectrum of the S0 state (right axis) and difference spectra of the T1 and Q1 states with the S0 state (left axis). B3LYP/6-311+G(d) PCM(acetonitrile), gaussian linewidth of $\sigma = 0.3$ eV. 23

Figure S10: Fluence dependence of NIR OTA signal. 1 mM $[FcCc]PF_6$ in MeCN, 0.1 cm path length, with 515 nm, 1 kHz pump focused to a 594 μm ($1/e^2$) diameter spot size. 25

Figure S11: Fluence dependence of UV-vis OTA signal. 0.25 mM $[FcCc]PF_6$ in MeCN, 0.2 cm path length, with 532 nm, 500 Hz pump focused to a 377 μm ($1/e^2$) diameter spot size. 26

Figure S12: Static UV-vis spectrum before and after UV-vis OTA experiment. 27
Figure S13: Solvent-only scan of acetonitrile with UV-vis probe and 532 nm pump. Coherent artifact signal was fit with a gaussian to determine the full width at half maximum (fwhm) of the instrument response function. 0.2 mM [FcCc]PF_6 in MeCN, 0.2 cm path length, with 532 nm, 500 Hz, 4 mJ/cm^2 pump... 29
Figure S14: Solvent-only scan of acetonitrile, with vis probe and 515 nm pump. Coherent artifact signal was fit with a gaussian to determine the full width at half maximum (fwhm) of the instrument response function. The three data points around time zero that deviate from the gaussian shape of the signal were excluded from the fit. 1 mM [FcCc]PF_6 in MeCN, 0.1 cm path length, with 515 nm, 1 kHz, 1.7 mJ/cm^2 pump... 30
Figure S15: 2D map of UV-vis transient absorption data of 0.2 mM [FcCc]PF_6 in MeCN, 0.2 cm path length, with 532 nm, 500 Hz, 4 mJ/cm^2 pump.. 31
Figure S16: Kinetic traces (thick lines) and global fit (thin lines) at select wavelengths of UV-vis transient absorption data of 0.2 mM [FcCc]PF_6 in MeCN, 0.2 cm path length, with 532 nm, 500 Hz, 4 mJ/cm^2 pump.. 32
Figure S17: Spectral slices of UV-vis transient absorption data of 0.2 mM [FcCc]PF_6 in MeCN, 0.2 cm path length, with 532 nm, 500 Hz, 4 mJ/cm^2 pump.. 33
Figure S18: Species-associated decay spectra (SADS) from target analysis to a 3-component sequential model of UV-vis transient absorption data of 0.2 mM [FcCc]PF_6 in MeCN, 0.2 cm path length, with 532 nm, 500 Hz, 4 mJ/cm^2 pump... 34
Figure S19: 2D map of vis transient absorption data of 1 mM [FcCc]PF_6 in MeCN, 0.1 cm path length, with 515 nm, 1 kHz, 1.7 mJ/cm^2 pump... 35
Figure S20: Kinetic traces (thick lines) and global fit (thin lines) at select wavelengths of vis transient absorption data of 1 mM [FcCc]PF_6 in MeCN, 0.1 cm path length, with 515 nm, 1 kHz, 1.7 mJ/cm^2 pump.. 36
Figure S21: Spectral slices of vis transient absorption data of 1 mM [FcCc]PF_6 in MeCN, 0.1 cm path length, with 515 nm, 1 kHz, 1.7 mJ/cm^2 pump... 37
Figure S22: Species-associated decay spectra (SADS) from target analysis to a 3-component sequential model of vis transient absorption data of 1 mM [FcCc]PF_6 in MeCN, 0.1 cm path length, with 515 nm, 1 kHz, 1.7 mJ/cm^2 pump... 38
Figure S23: 2D map of NIR transient absorption data of 1 mM [FcCc]PF_6 in MeCN, 0.1 cm path length, with 515 nm, 1 kHz, 1.7 mJ/cm^2 pump... 39
Figure S24: Kinetic traces (thick lines) and global fit (thin lines) at select wavelengths of NIR transient absorption data of 1 mM [FcCc]PF_6 in MeCN, 0.1 cm path length, with 515 nm, 1 kHz, 1.7 mJ/cm^2 pump.. 40
Figure S25: Spectral slices of NIR transient absorption data of 1 mM [FcCc]PF_6 in MeCN, 0.1 cm path length, with 515 nm, 1 kHz, 1.7 mJ/cm^2 pump.. 41
Figure S26: Species-associated decay spectra (SADS) from target analysis to a 3-component sequential model of NIR transient absorption data of 1 mM [FcCc]PF_6 in MeCN, 0.1 cm path length, with 515 nm, 1 kHz, 1.7 mJ/cm^2 pump... 42
Figure S27: 2D map of SWIR transient absorption data of 1 mM [FcCc]PF_6 in MeCN, 0.1 cm path length, with 515 nm, 1 kHz, 3.3 mJ/cm^2 pump... 43

Figure S28: Kinetic traces (thick line) and global fit (thin line) at select wavelength of SWIR transient absorption data of 1 mM [FcCc]PF_6 in MeCN, 0.1 cm path length, with 515 nm, 1 kHz, 3.3 mJ/cm^2 pump. 44
Figure S29: Spectral slices of SWIR transient absorption data of 1 mM [FcCc]PF_6 in MeCN, 0.1 cm path length, with 515 nm, 1 kHz, 3.3 mJ/cm^2 pump. 45
Figure S30: Spectral slices of SWIR transient absorption data of 1 mM [FcCc]PF_6 in MeCN, 0.1 cm path length, with 515 nm, 1 kHz, 3.3 mJ/cm^2 pump. Probe spectrum is shown for comparison. The dip in the transient spectra at 1725 nm are likely an artifact caused by instabilities of the probe at the minimum of intensity at the same wavelength. 46
Figure S31: Species-associated decay spectra (SADS) from target analysis to a 3-component sequential model of SWIR transient absorption data of 1 mM [FcCc]PF_6 in MeCN, 0.1 cm path length, with 515 nm, 1 kHz, 3.3 mJ/cm^2 pump. 47
Figure S32: Comparison of signal magnitudes of UV-vis and vis OTA data at 2 ps. The UV-vis data has been scaled to account for differences in concentration, path length, fluence, and extinction coefficient. 49
Figure S33: Comparison of signal magnitudes of UV-vis and vis OTA data at 2 ps after normalizing the UV-vis data by a factor of 10 to match the intensity of the bleach feature at 550 nm in the vis data set. 50
Figure S34: Spectral slices of NIR and SWIR OTA data. SWIR data have been scaled by 0.6 to account for higher fluence. 51
Figure 35: Static XANES and derivative spectrum of metallic Fe foil. Peak of first derivative is compared to reference value[1]: Bearden et al. Rev. Mod. Phys. 1967, 39 (1), 125–142. 53
Figure 36: Static XANES and derivative spectrum of metallic Co foil. Peak of first derivative is compared to reference value[1]: Bearden et al. Rev. Mod. Phys. 1967, 39 (1), 125–142. 53
Figure S37: Fluence dependence of of Fe K edge XTA signal at the 7.124 keV ESA maximum. 5 mM [FcCc]PF_6 in MeCN, 700 µm cylindrical liquid jet, 515 nm, 3 kHz, 20 mJ/cm^2 pump focused to a 692 µm (1/e^2) diameter spot size. 54
Figure S38: UV-vis spectrum of XTA sample before and after XTA measurement. The sample solution (5 mM [FcCc]PF_6 in acetonitrile) was diluted prior to the UV-vis measurement, and the spectra were normalized to the peak at 350 nm. 55
Figure S39: Individual scan points of Fe K-edge XTA spectrum at 150 ps. Each scan is represented by a different color. Static spectrum is shown in grey and plotted on the right axis. 5 mM [FcCc]PF_6 in MeCN, 700 µm cylindrical liquid jet, 515 nm, 3 kHz, 20 mJ/cm^2 pump. 58
Figure S40: Individual scan points of Co K-edge XTA spectrum at 150 ps. Each scan is represented by a different color. Static spectrum is shown in grey and plotted on the right axis. 5 mM [FcCc]PF_6 in MeCN, 700 µm cylindrical liquid jet, 515 nm, 3 kHz, 20 mJ/cm^2 pump. 59
Figure S41: Histograms of the XTA signal at the Co K-edge bleach (7732.9 ± 1 eV) and Fe K-edge excited-state absorption (7124.8 ± 0.5 eV). The histograms were fit to gaussian functions characterized by the center point, x_0, and standard deviation, σ. 60
Figure S42: Effect of outlier rejection on Fe K-edge EXAFS spectra. The tolerance was defined as the number of standard deviations away from the mean that a data point had to be to be

considered an outlier and rejected from the final calculation of the average and standard deviation. 61
Figure S43: Comparison of the standard deviation and 99.9% confidence interval of the Fe K-edge transient EXAFS data at 150 ps. 5 mM $[FcCc]PF_6$ in MeCN, 700 µm cylindrical liquid jet, 515 nm, 3 kHz, 20 mJ/cm^2 pump. 62
Figure S44: Comparison of the standard deviation and 99.9% confidence interval of the Fe K-edge transient XANES data at 150 ps. 5 mM $[FcCc]PF_6$ in MeCN, 700 µm cylindrical liquid jet, 515 nm, 3 kHz, 20 mJ/cm^2 pump. 63
Figure S45: Comparison of the standard deviation and 99.9% confidence interval of the Fe K-edge transient XANES data at 150 ps. 5 mM $[FcCc]PF_6$ in MeCN, 700 µm cylindrical liquid jet, 515 nm, 3 kHz, 20 mJ/cm2 pump. 64
Figure S46: Kinetic trace of Fe K-edge XTA signal at 7.124 keV. Error bars are the standard deviation of the data. The data were fit to a single exponential convoluted with a gaussian instrument response function (IRF). 5 mM $[FcCc]PF_6$ in MeCN, 700 µm cylindrical liquid jet, 515 nm, 3 kHz, 20 mJ/cm2 pump. 65
Figure S47: Fe 1s pre-edge XTA spectrum and TD-DFT calculations. TD-DFT sticks and broadened spectra have been shifted by +22.8 eV to align with experiment. The sticks (plotted on the right axis) have been scaled by 0.3 for clarity. Error bars are ± 99.9% confidence interval. 66
Figure S48: Atom labels for EXAFS scattering paths. 69
Figure S49: Static Fe K-edge EXAFS spectrum in energy space and background spectrum. 5 mM $[FcCc]PF_6$ in MeCN, 700 µm cylindrical liquid jet. 70
Figure S50: Static Fe K-edge EXAFS spectrum in k-space following background subtraction. Spectra with various k-weights are shown. 5 mM $[FcCc]PF_6$ in MeCN, 700 µm cylindrical liquid jet. 71
Figure S51: Real part of the background-subtracted static EXAFS in *k* space and corresponding fits (black traces). Traces are vertically offset by 2 and scaled by the specified factors for clarity. 74
Figure S52: Magnitude of Fourier transform of static EXAFS in position space and corresponding fits (black traces). 75
Figure S53: Real part of Fourier transform of static EXAFS in position space and corresponding fits (black traces). 76
Figure S54: Change in distance between C atoms 1 and 6 as a function of Fe-C bond length (R) and Fe-C bond length increase (ΔR) for excited-state EXAFS model. 80
Figure S55: Change in distance between C atoms 1 and 8 as a function of Fe-C bond length (R) and Fe-C bond length increase (ΔR) for excited-state EXAFS model. See Figure S54 for expression of d_{16}. 81
Figure S56: Change in distance between C atoms 1 and 7 as a function of Fe-C bond length (R) and Fe-C bond length increase (ΔR) for excited-state EXAFS model. See Figure S54 for expression of d_{16}. 82
Figure S57: Change in distance between Fe atom and C atom 1' of the cobaltocenium ring, expressed as a function of Fe-C bond length (R) and Fe-C bond length increase (ΔR) for excited-state EXAFS model. See Figure S54 for expression of d_{16}. 83

Figure S58: Change in distance between Fe atom and C atom 2’ of the cobaltocenium ring, expressed as a function of Fe-C bond length (R) and Fe-C bond length increase (ΔR) for excited-state EXAFS model. See Figure S54 for expression of d_{16}. 84
Figure S59: Reduced chi-squared surfaces along change in bond length for various shifts in absorption edge. 86
Figure S60: Reduced chi-squared surface along change in absorption edge for an excitation fraction of 3% and change in bond length of 0.25 Å. 87
Figure S61: Reduced chi-squared surface along change in bond length compared to changes in bond length calculated by DFT. The DFT values, given by the vertical dashed lines, represent the change in average Fe-C bond length between the S_0 state and the T_1 (vertical blue dashed line) or Q_1 (vertical blue dashed line) states. The horizontal dashed line represents $\Delta\chi^2 = +1$ from the minimum and bounds the 68% confidence region ($\pm1\sigma$) of the fitted ΔR parameter. 88
Figure S62: Comparison of XANES spectra calculated with FEFF10 with experimental static spectrum. Calculated spectra have been shifted by -10 eV to better match the experimental spectrum. 96
Figure S63: Comparison of EXAFS spectra calculated with FEFF10 with experimental static spectrum. Calculated spectra have been shifted by -10 eV to better match the experimental spectrum. 97
Figure S64: Calculation of the activation energy of ^{3}(d-d) → ^{5}(d-d) ISC with Equation 8 for a range of temperature and exponential prefactor values, with a time constant of $\tau = 30$ ps, as measured by OTA 99

1 Synthetic details

All air- and moisture-sensitive operations, including basic Schlenk and glovebox techniques, were performed using oven-dried glassware under a nitrogen atmosphere if not indicated otherwise. All reagents for which the syntheses are not given were purchased from Sigma-Aldrich, Acros, STREM, or Pressure Chemical and were used as received without further purification. Solvents were purified prior to use by passing through a column of activated alumina using an MBRAUN SPS. The synthesis of ferrocenyl cobaltocenium hexafluorophosphate followed a slightly modified procedure from the literature.[1,2]

Synthesis of lithioferrocene (FcLi)

Scheme 1

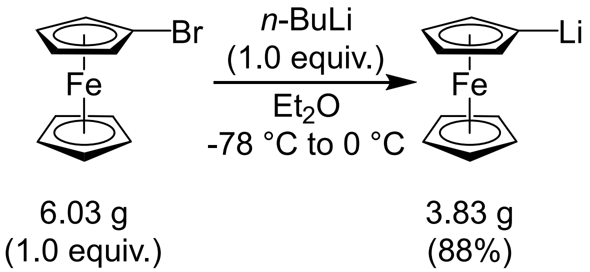

Ferrocenyl bromide (6.03 g, 22.8 mmol, 1.0 equiv) was added to a 500 mL round bottom flask, followed by the addition of 150 mL of diethyl ether at -78 °C. An equivalent amount of *n*-butyllithium (14.2 mL of 1.6 M in hexanes, 22.8 mmol, 1.0 equiv) was then gradually added dropwise over 10 minutes to the flask. The reaction mixture was gently warmed to 0 °C using an ice water bath with continuous stirring. An orange precipitate was observed at -13 °C. After reaching 0 °C, the solution was stirred for an additional 20 minutes. The resulting orange precipitate was subsequently isolated by vacuum filtration and dried, yielding orange lithioferrocene (3.83 g, 88%).

Synthesis of ferrocenyl cobaltocenium hexafluorophosphate

Scheme 2

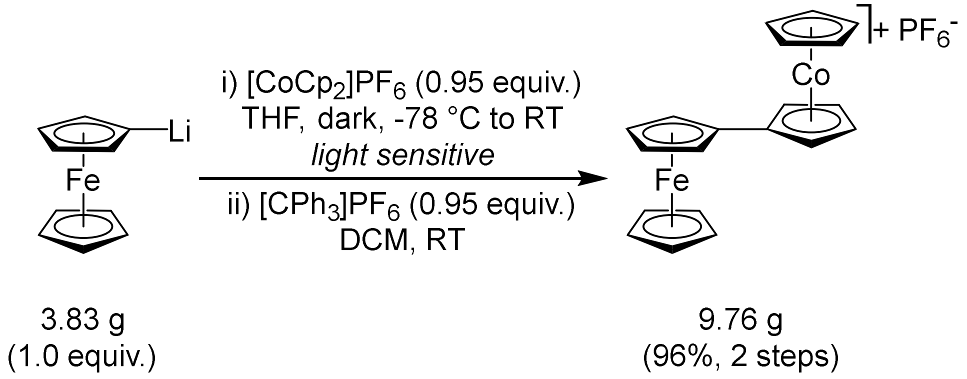

Cobaltocenium hexafluorophosphate (6.33 g, 19.0 mmol, 0.95 equiv relative to FcLi) is added to a 1 L round bottom flask, followed by the addition of 200 mL of THF. The mixture was then cooled to -78 °C. A solution of lithioferrocene (3.83 g, 20.0 mmol, 1.0 equiv) in THF (200 mL) was prepared and added dropwise to the reaction flask in the dark. The reaction mixture was stirred for 14 hours in the dark, with the flask covered in aluminum foil, and gradually allowed to warm to room temperature. A red-colored homogeneous solution was obtained, and volatiles were dried *in vacuo* in the dark. (*Note: This is a light-sensitive reaction.*) The resulting dried solid was dissolved in 150 mL of dichloromethane and filtered through a pad of Celite. To the filtrate, triphenylmethyl hexafluorophosphate (7.36 g, 19.0 mmol, 0.95 equiv) was added. The solution rapidly changed to a deep blue color within minutes of stirring at room temperature, and the mixture was stirred for an additional 30 minutes. (*Note: the deep blue colored solution is no longer light-sensitive.*) The reaction flask was then removed from the glovebox, and volatiles were dried *in vacuo.* The resulting solid was washed sequentially with diethyl ether (10 mL) and then with distilled cold water (3 mL) nine times, until the washing solution is colorless. The washed solid was dried *in vacuo* to afford the dark blue product (9.76 g, 96%). The spectral data were in agreement with the literature values.[2] ^{1}H NMR (CD_3CN, 500 MHz) δ (ppm): 5.84 (br, 2H), 5.65 (br, 2H), 5.37 (br, 5H), 4.74 (br, 2H), 4.56 (br, 2H), 4.08 (br, 5H).

Figure S1: 1H NMR spectrum of ferrocenyl cobaltocenium hexafluorophosphate at 23 °C (CD_3CN).

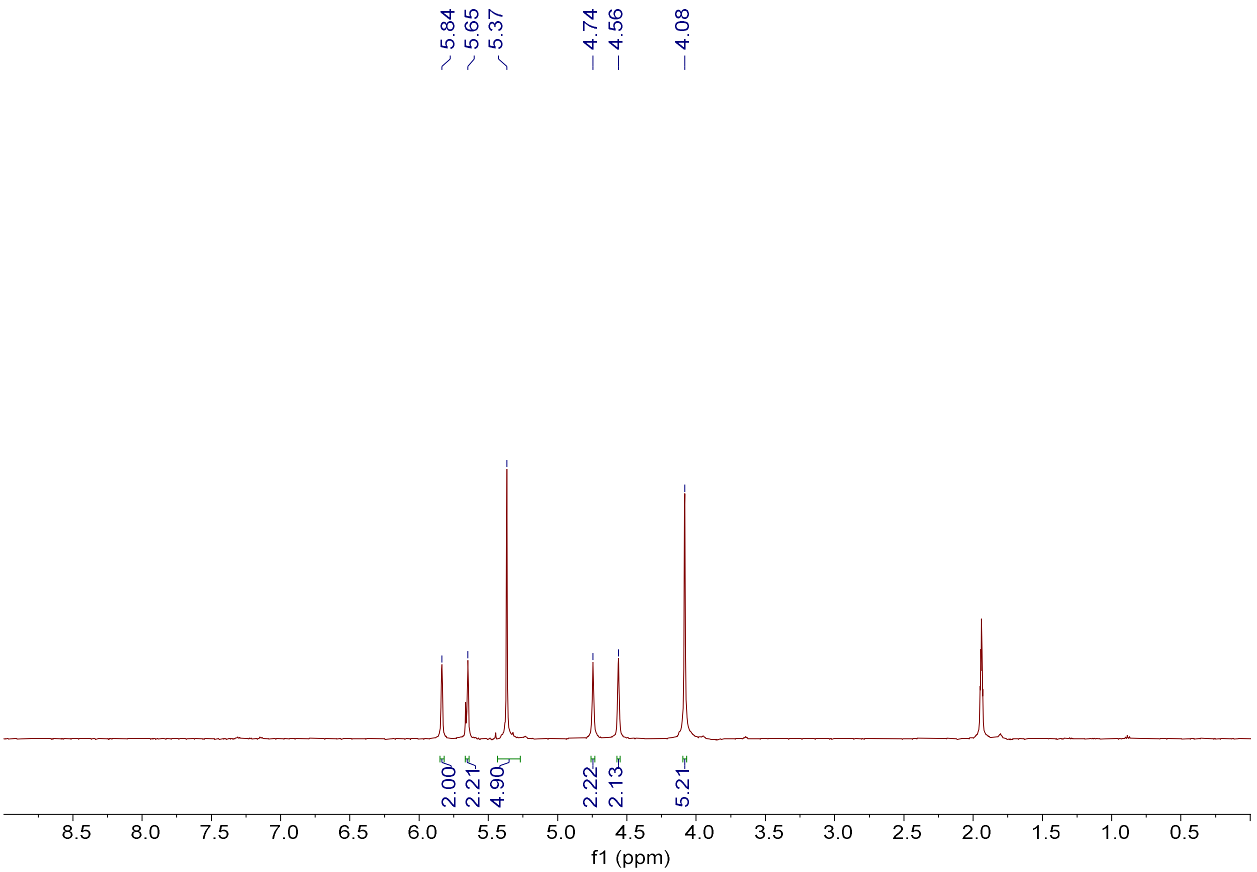

2 Density Functional Theory

Full Gaussian Citation:
Gaussian 16, Revision C.01,
M. J. Frisch, G. W. Trucks, H. B. Schlegel, G. E. Scuseria,
M. A. Robb, J. R. Cheeseman, G. Scalmani, V. Barone,
G. A. Petersson, H. Nakatsuji, X. Li, M. Caricato, A. V. Marenich,
J. Bloino, B. G. Janesko, R. Gomperts, B. Mennucci, H. P. Hratchian,
J. V. Ortiz, A. F. Izmaylov, J. L. Sonnenberg, D. Williams-Young,
F. Ding, F. Lipparini, F. Egidi, J. Goings, B. Peng, A. Petrone,
T. Henderson, D. Ranasinghe, V. G. Zakrzewski, J. Gao, N. Rega,
G. Zheng, W. Liang, M. Hada, M. Ehara, K. Toyota, R. Fukuda,
J. Hasegawa, M. Ishida, T. Nakajima, Y. Honda, O. Kitao, H. Nakai,
T. Vreven, K. Throssell, J. A. Montgomery, Jr., J. E. Peralta,
F. Ogliaro, M. J. Bearpark, J. J. Heyd, E. N. Brothers, K. N. Kudin,
V. N. Staroverov, T. A. Keith, R. Kobayashi, J. Normand,
K. Raghavachari, A. P. Rendell, J. C. Burant, S. S. Iyengar,
J. Tomasi, M. Cossi, J. M. Millam, M. Klene, C. Adamo, R. Cammi,
J. W. Ochterski, R. L. Martin, K. Morokuma, O. Farkas,
J. B. Foresman, and D. J. Fox, Gaussian, Inc., Wallingford CT, 2019.

Density functional theory (DFT) calculations were performed in Gaussian 16. All DFT calculations were performed using the B3PW91[3,4] or B3LYP[3,5] functionals and 6-311+G(d)[6–9] or LANL2DZ[10,11] basis sets. Structures were optimized to stationary points which were determined to be local minima by performing frequency calculations and verifying the absence of imaginary frequencies. Single point and time-dependent density functional theory calculations were performed at the same level of theory as the geometry optimizations. Implicit solvation was included through the polarizable continuum model (PCM)[12]. Thermal corrections[13] to free energies assumed an ideal gas at 298.15 K and 1 atm. Time-dependent (TD-)DFT calculations found the first 150 excited states. Molecular geometries were visualized with GaussView 6. Canonical and natural transition orbitals were visualized with isovalues of 0.02 using Chemissian. Broadened TD-DFT spectra in extinction coefficient units of $M^{-1}cm^{-1}$ were calculated with gaussian lineshapes of σ = 0.3 eV according to Equation S1.[14]

$$\varepsilon(\tilde{\upsilon}) = \sum_i 1.3062974 \cdot 10^8 \cdot \frac{f_i}{\sigma} \exp\left[-\left(\frac{(\tilde{\upsilon} - \tilde{\upsilon}_i)}{\sigma}\right)^2\right]$$

Equation S1

where f_i and $\tilde{\upsilon}_i$ are the oscillator strength and transition frequency (in cm^{-1}) of the *i*-th transition, and σ is the gaussian width (standard deviation), which was taken to be 0.3 eV.

2.1 Choice of Model Chemistry

The level of TD-DFT theory was chosen based on agreement with the experimental UV-vis spectrum (Figure S2). Warratz et al.[2] previously calculated the electronic transitions of $[FcCc]^+$ with B3LYP/LANL2DZ. This level of theory successfully reproduces the relative transition energies and intensities, but the absolute transition energies are 0.69 eV lower than experiment. In contrast, Wagenknecht and coworkers[15,16] showed that B3PW91/6-311+G(d) with the polarizable continuum model (PCM) treatment of solvent faithfully reproduces the experimental MMCT energies of bis-ferrocenyl titanocene complexes. We found that this level of theory also reproduces the MMCT energy of $[FcCc]^+$, and exclusively use B3PW91/6-311+G(d) PCM(acetonitrile) in the main text. In the SI, we also include supplemental B3LYP/6-311+G(d) PCM(acetonitrile) calculations.

Figure S2: Comparison of TD-DFT spectra of $[FcCc]^+$ with the experimental UV-vis spectrum of $[FcCc]PF_6$ in acetonitrile. TD-DFT oscillator strength sticks (right axis) have been broadened with gaussians lineshapes of σ = 0.3 eV.

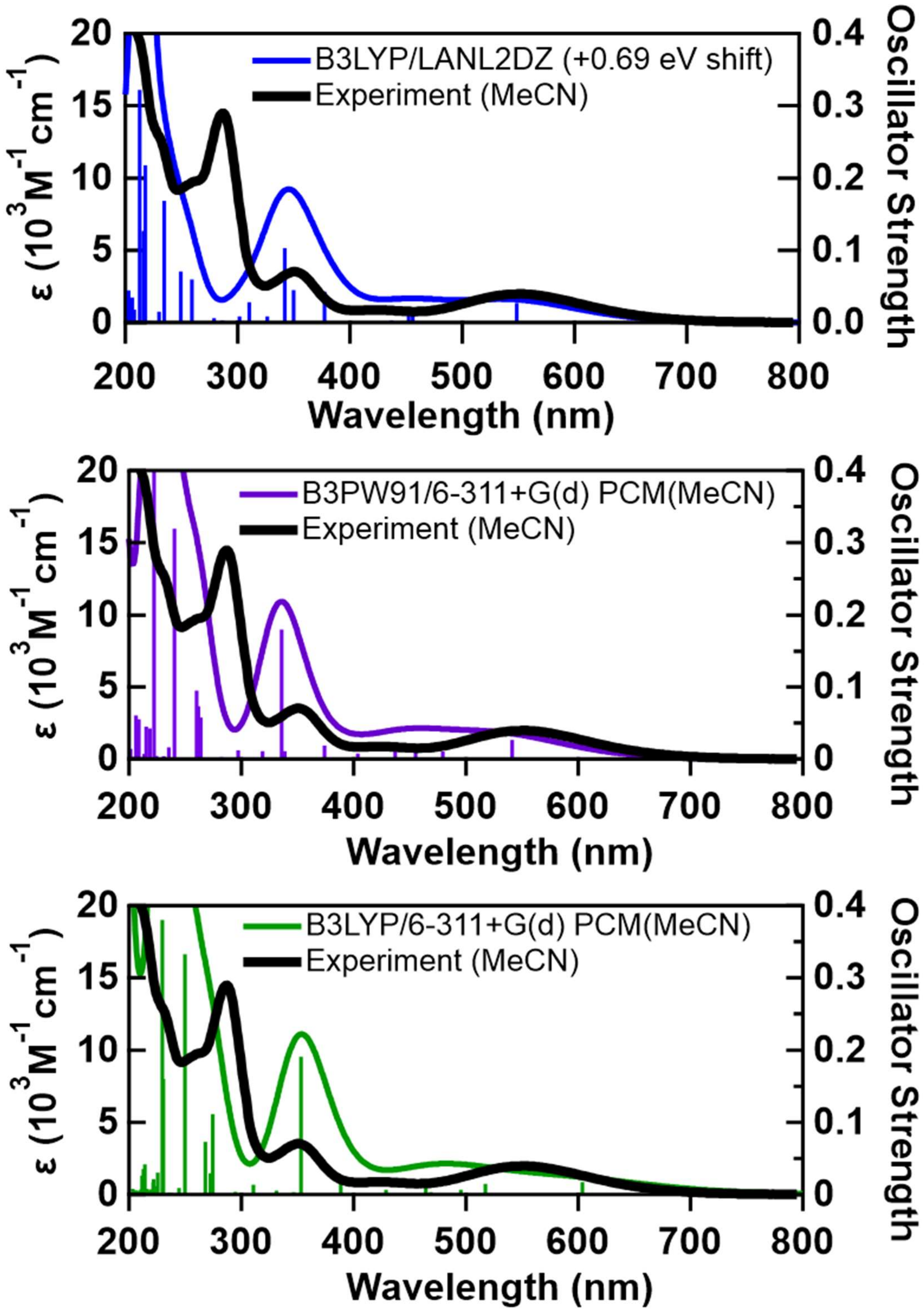

Figure S3: UV-vis spectrum (left axis) of $[FcCc]PF_6$ in acetonitrile (MeCN) overlaid with TD-DFT stick spectrum (right axis) of $[FcCc]^+$ calculated at B3PW91/6-311+G(d) PCM(acetonitrile).

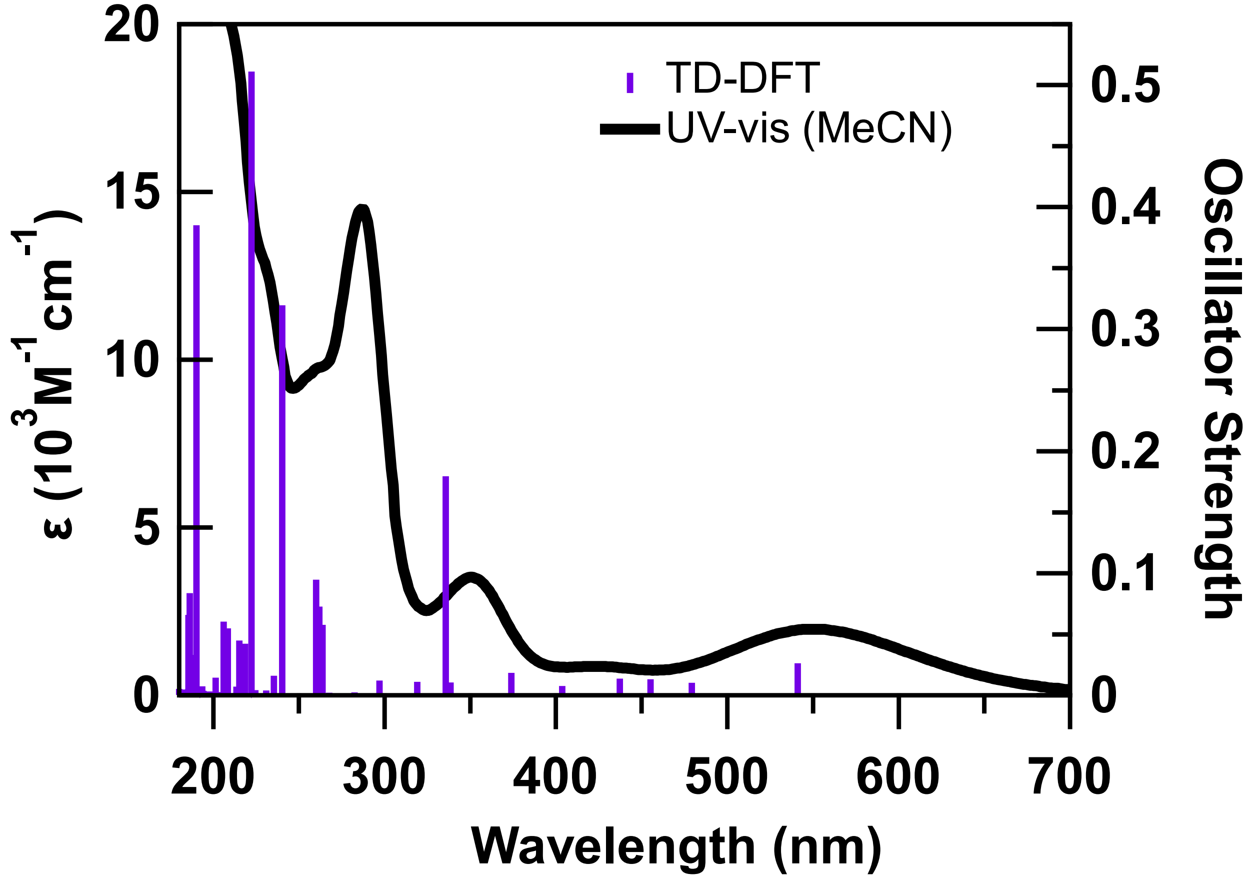

Table S1: Selected TD-DFT transitions of $[FcCc]^{+}$ at B3PW91/6-311+G(d) PCM(acetonitrile) level. Natural transition orbitals depicted are the highest contributing and visualized at 0.02 isovalue. Symmetry labels are irreducible representations of the C_s point group.

Transition	Assignment	Symmetry	Energy (eV) / Wavelength (nm)	Oscillator Strength	Natural Transition Orbitals (Hole/Electron)	
$S_0 \rightarrow S_1$	MMCT	A”	2.248 / 551.5	0.0002	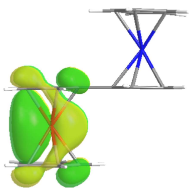	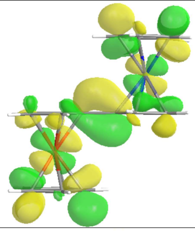
$S_0 \rightarrow S_2$	MMCT	A’	2.291 / 541.1	0.0263	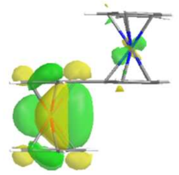	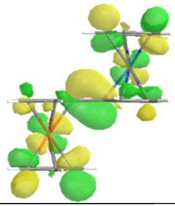
$S_0 \rightarrow S_{18}$	LM’CT	A’	3.693 \ 335.7	0.1796	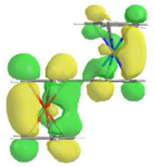	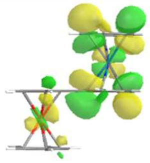
$S_0 \rightarrow S_{33}$	LMCT	A’	5.159 / 240.3	0.3196	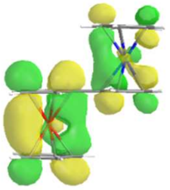	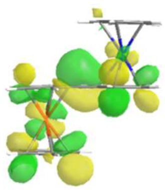
$S_0 \rightarrow S_{45}$	LMCT	A’	5.577 / 222.3	0.5111	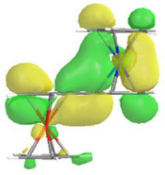	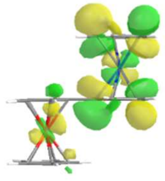

Figure S4: Relative self-consistent field (SCF) energies and Gibbs free energies (at 298 K and 1 atm) of the lowest singlet (S0), triplet (T1) and quintet (Q1) states of $[FcCc]^{+}$ at two different levels of theory. Both calculations include PCM(acetonitrile) implicit solvation. The Gibbs free energies of the triplet and quintet states contain contributions from the electronic entropy due to spin degeneracy.

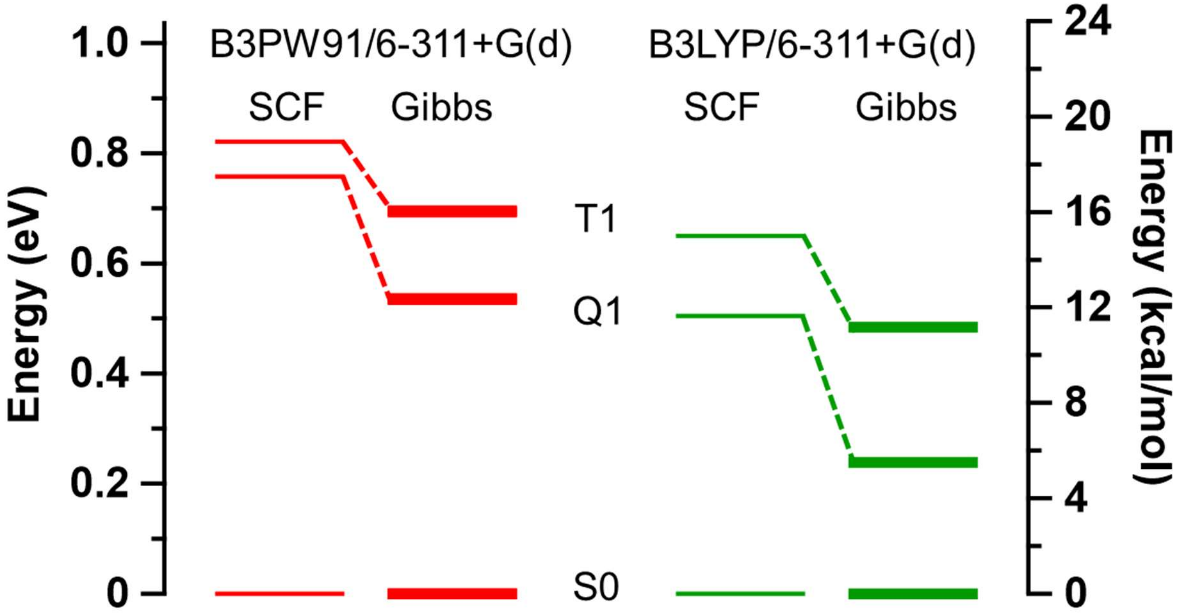

Figure S5: Energy barrier between T1 and Q1 states. Single point energy calculations were performed at geometries interpolated between the optimized T1 and Q1 states. Relative SCF energy is plotted here as a function of geometry interpolation point. B3PW91/6-311+G(d) PCM(acetonitrile).

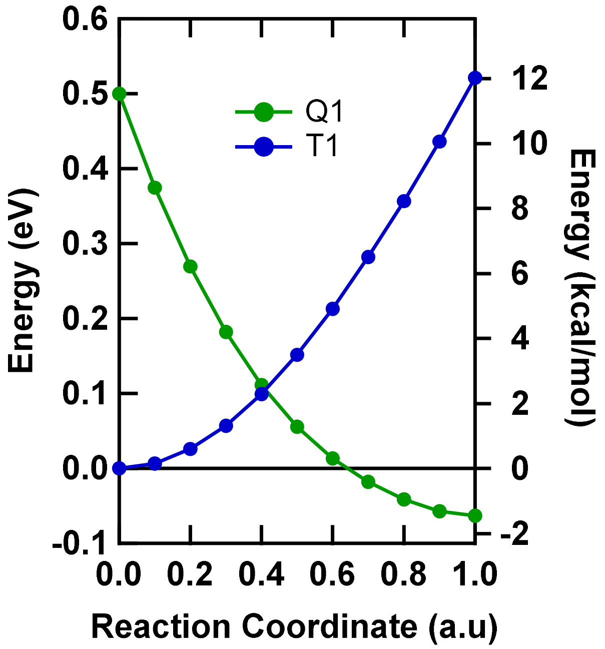

Figure S6: TD-DFT vertical transition energies of the S0, T1, and Q1 states, offset by their relative SCF energy. Assignments of states are given based on visual inspection of natural transition orbitals. B3PW91/6-311+G(d) PCM(acetonitrile).

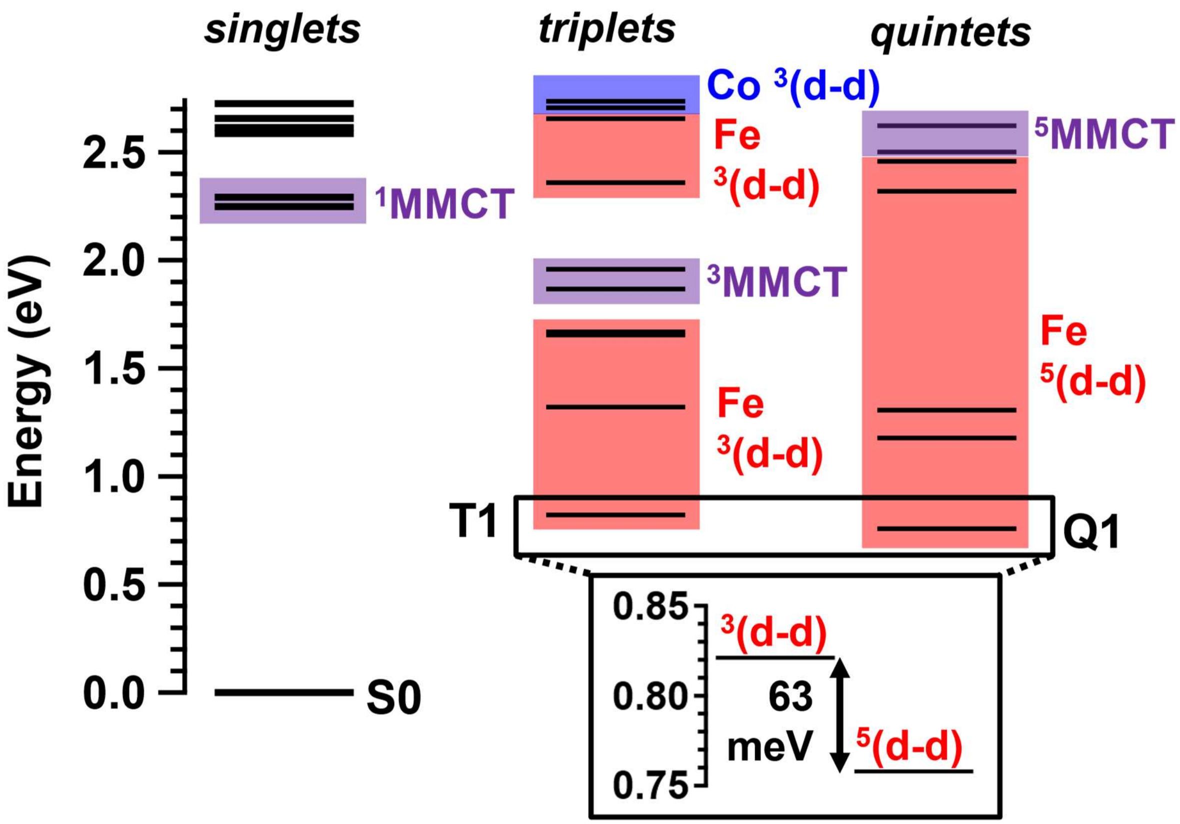

Table S2: $\langle \hat{S}^2 \rangle$ expectation values before and after annihilation of the first spin contaminant of the lowest triplet (T_1) and quintet (Q_1) states of $[FcCc]^+$ with the 6-311+G(d) basis set, PCM(acetonitrile) solvent model, and either the B3PW91 or B3LYP functional.

		B3PW91		B3LYP	
	Ideal	Before	After	Before	After
T_1	2.0	2.1149	2.0060	2.1205	2.0064
Q_1	6.0	6.0220	6.0001	6.0225	6.0001

Table S3: Selected TD-DFT transitions of the lowest triplet (T_1) state of $[FcCc]^+$ at B3PW91/6-311+G(d) PCM(acetonitrile) level. Natural transition orbitals depicted are the highest contributing and visualized at 0.02 isovalue.

Transition	Assignment	Energy (eV) / Wavelength (nm)	Oscillator Strength	Natural Transition Orbitals (Hole/Electron)
$T_1 \rightarrow T_5$	MMCT	1.046 / 1185.6	0.172	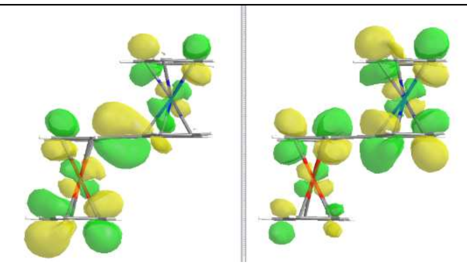 α → α
$T_1 \rightarrow T_{27}$	LMCT	3.484 / 355.8	0.156	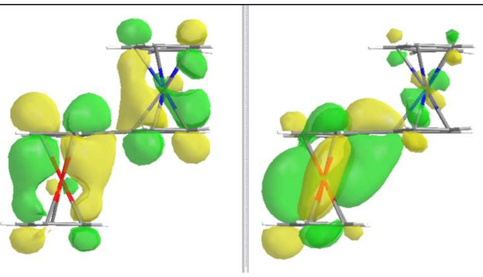 α → α

Table S4: Selected TD-DFT transitions of the lowest quintet (Q_1) state of $[FcCc]^+$ at B3PW91/6-311+G(d) PCM(acetonitrile) level. Natural transition orbitals depicted are the highest contributing and visualized at 0.02 isovalue.

Transition	Assignment	Energy (eV) / Wavelength (nm)	Oscillator Strength	Natural Transition Orbitals (Hole/Electron)
$Q_1 \rightarrow Q_{18}$	LM'CT	2.944 / 421.2	0.075	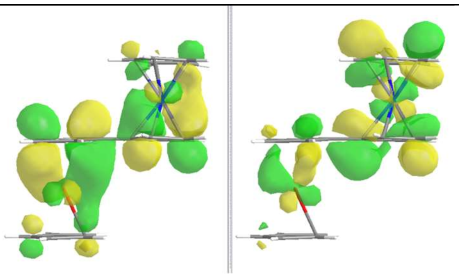 β → β
$Q_1 \rightarrow Q_{28}$	LM'CT	2.291 / 541.1	0.100	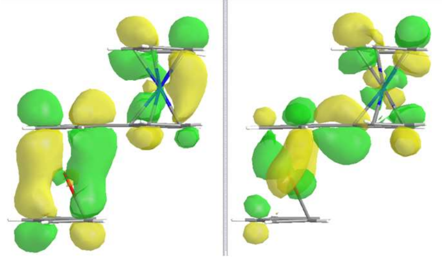 β → β

Figure S7: Broadened TD-DFT spectra (traces, left axis) and oscillator strengths (sticks, right axis) of the lowest singlet (S0), triplet (T1), and quintet (Q1) states of $[FcCc]^+$ calculated with B3PW91/6-311+G(d) PCM(acetonitrile) and gaussian broadening of linewidth σ = 0.3 eV.

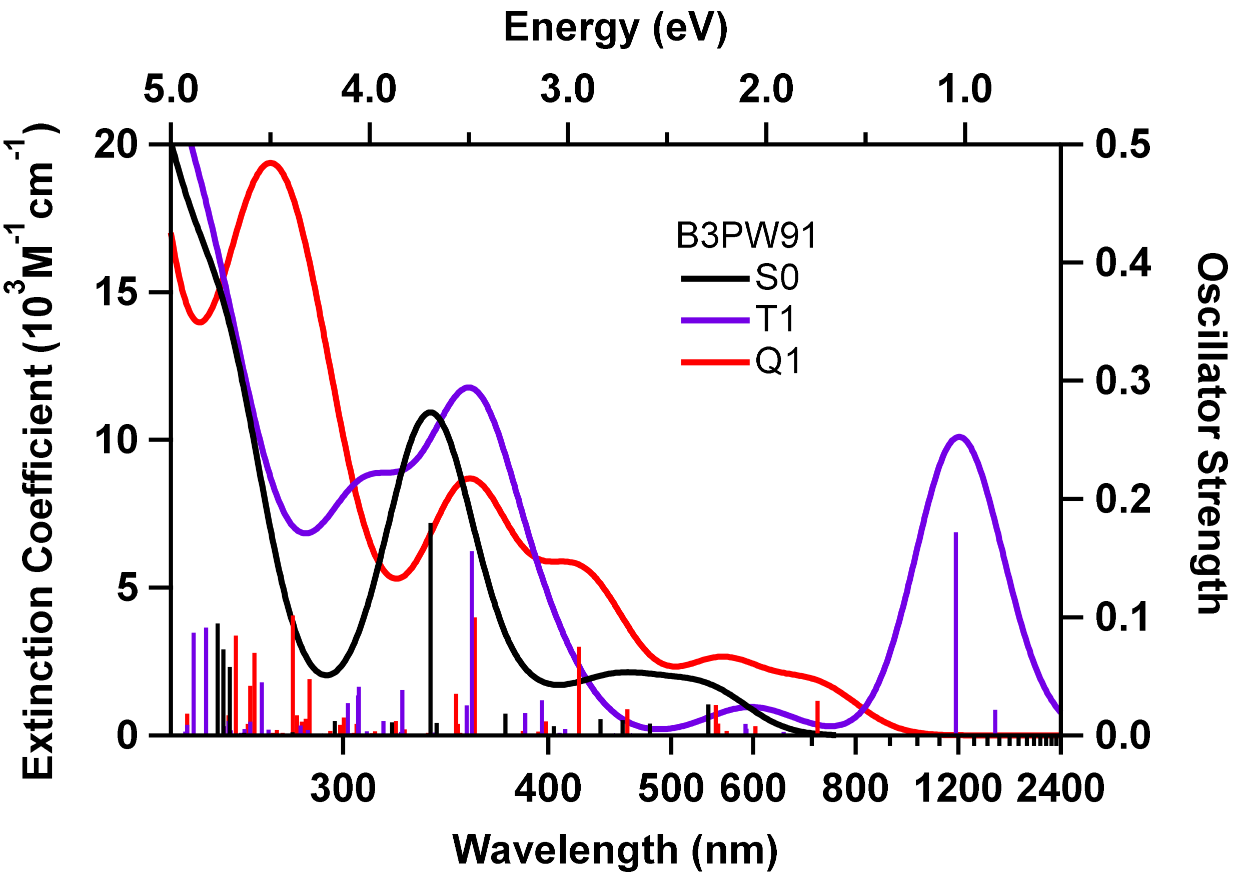

Figure S8: Broadened TD-DFT spectra (traces, left axis) and oscillator strengths (sticks, right axis) of the lowest singlet (S0), triplet (T1), and quintet (Q1) states of $[FcCc]^+$ calculated with B3LYP/6-311+G(d) PCM(acetonitrile) and gaussian broadening of linewidth σ = 0.3 eV.

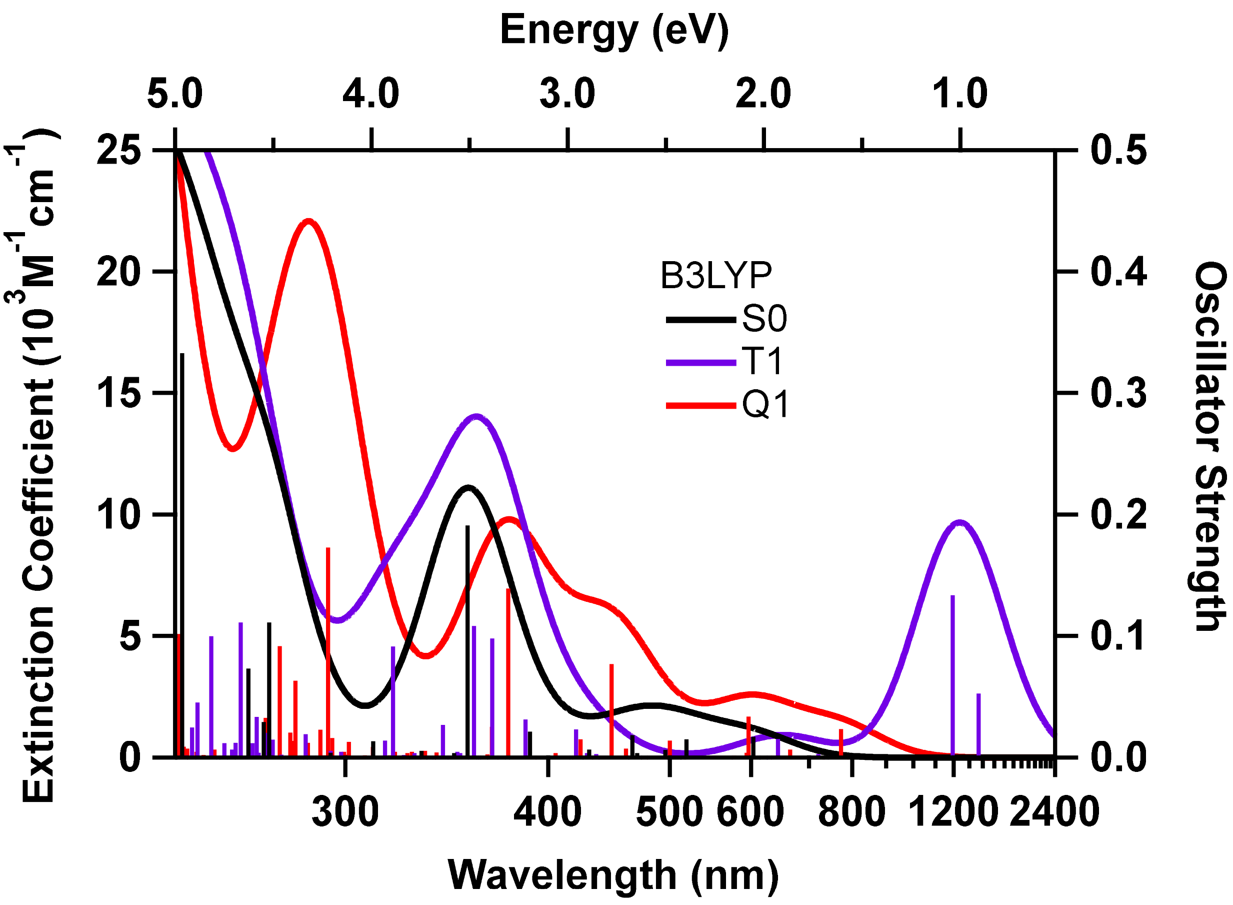

Figure S9: Comparison of OTA and TD-DFT spectra. Top panel: experimental static (right axis) and transient (left axis) spectra at 0.3 ps, 2 ps and 200 ps. The UV-vis and SWIR OTA data have been scaled to be on the same vertical scale as the vis and NIR OTA data; see text for details. Bottom panel: broadened TD-DFT spectrum of the S0 state (right axis) and difference spectra of the T1 and Q1 states with the S0 state (left axis). B3LYP/6-311+G(d) PCM(acetonitrile), gaussian linewidth of σ = 0.3 eV.

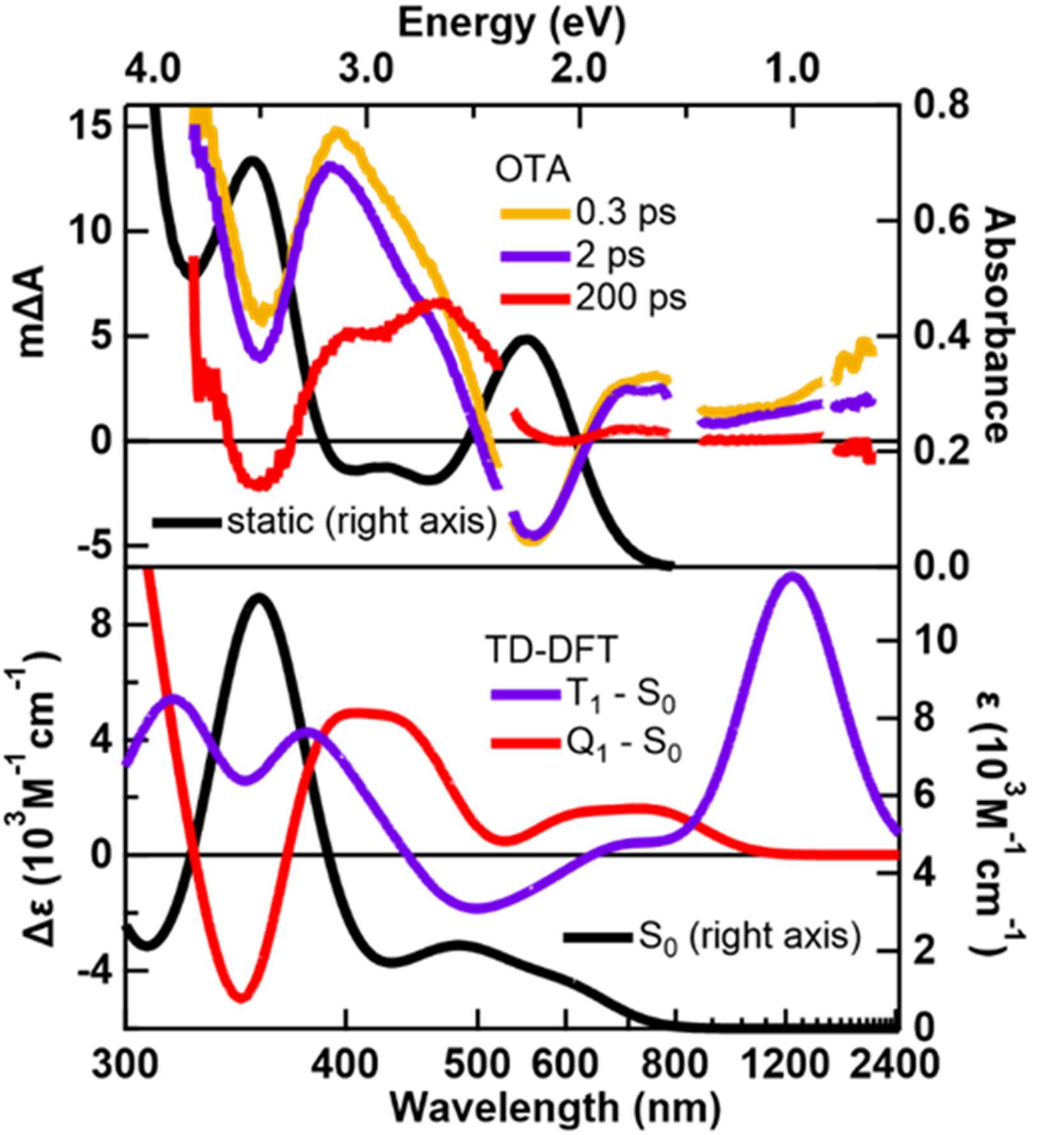

3 Optical transient absorption spectroscopy

3.1 General Considerations

Optical transient absorption (OTA) spectroscopy was used to characterize the excited-state dynamics initiated by MMCT excitation of $[FcCc]PF_6$ in acetonitrile. We performed OTA experiments with different probe pulses to cover a broad spectral range spanning from the ultraviolet (UV), visible (vis), near-infrared (NIR), and short-wave infrared (SWIR).

UV-vis OTA experiments were performed at the Materials Research Laboratory Central Research Facilities at the University of Illinois. A Ti:sapphire regenerative amplifier (Spitfire, Spectra-Physic) produced 35 fs pulses centered at 800 nm at a repetition rate of 2 kHz, which pumped an optical parametric amplifier (OPA) (TOPAS Twins/ NirUVis, Light Conversion) to generate 532 nm pump pulses which were reduced to 500 Hz with a mechanical chopper. Probe pulses in the UV-vis range of 320-650 nm were produced through white light generation (WLG) in CaF_2 pumped with a portion of the 800 nm output of the amplifier.

To probe at longer wavelengths, we performed experiments at the Center for Nanoscale Materials, a U.S. Department of Energy Office of Science User Facility located at Argonne National Laboratory. A Ti:sapphire regenerative amplifier (Spitfire, Spectra-Physic) produced 120 fs pulses centered at 800 nm at a repetition rate of 1 kHz, which pumped an OPA (Light Conversion) to generate 515 nm pump pulses which were reduced to 500 Hz with a mechanical chopper. Probe pulses in the vis (420-850 nm) and NIR (850-1500 nm) regions were generated by WLG in a 2 mm or 8 mm sapphire crystal, respectively, pumped by a portion of the 800 nm output of the amplifier. Probe pulses in the SWIR (1500-2100 nm) range were generated as the idler pulse in an OPA pumped with 800 nm pulses.

The vis, NIR, and SWIR experiments were performed with depolarized probe pulses to reduce the contribution of signal anisotropy. The UV-vis experiments, on the other hand, were performed with perpendicular pump-probe polarization, and thus likely contain contribution from rotational diffusion of the initially anisotropic ensemble of excited-state molecules. Such rotational randomization typically occurs on the ~10-100 ps timescale. Nevertheless, the UV-vis and vis data sets exhibit similar spectral features and time constants.

The UV-vis, vis, and NIR OTA data were corrected for the group-velocity dispersion (GVD) of the probe by fitting time zero versus probe energy to a 2nd order polynomial. The SWIR data showed little GVD and were thus not corrected.

Laser fluence and beam area were calculated from the 1/e^2 beam diameter (d_{1/e^2}) as

$$\text{Beam area}(cm^2) = \frac{1}{2}\pi\left(\frac{d_{1/e^2}(cm)}{2}\right)^2$$

Equation S2

$$\text{Fluence}(\frac{mJ}{cm^2}) = \frac{\text{Average power}(mW)}{\text{Repetition rate}(Hz) \times \text{Beam area}(cm^2)}$$

Equation S3

Figure S10: Fluence dependence of NIR OTA signal. 1 mM [FcCc]PF_6 in MeCN, 0.1 cm path length, with 515 nm, 1 kHz pump focused to a 594 µm (1/e^2) diameter spot size.

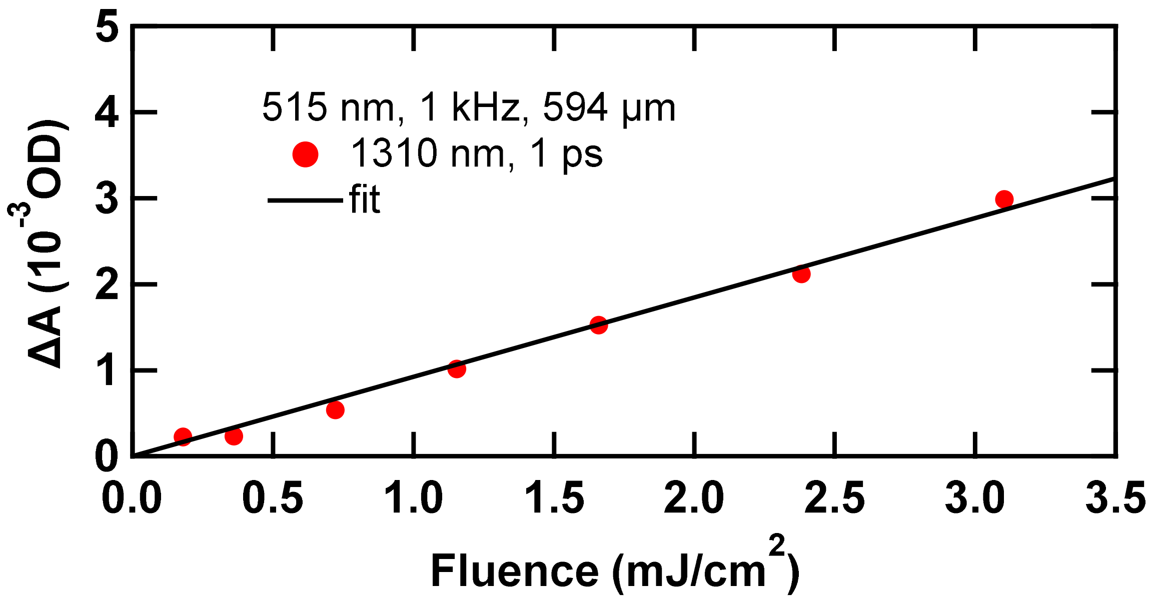

Figure S11: Fluence dependence of UV-vis OTA signal. 0.25 mM $[FcCc]PF_6$ in MeCN, 0.2 cm path length, with 532 nm, 500 Hz pump focused to a 377 μm ($1/e^2$) diameter spot size.

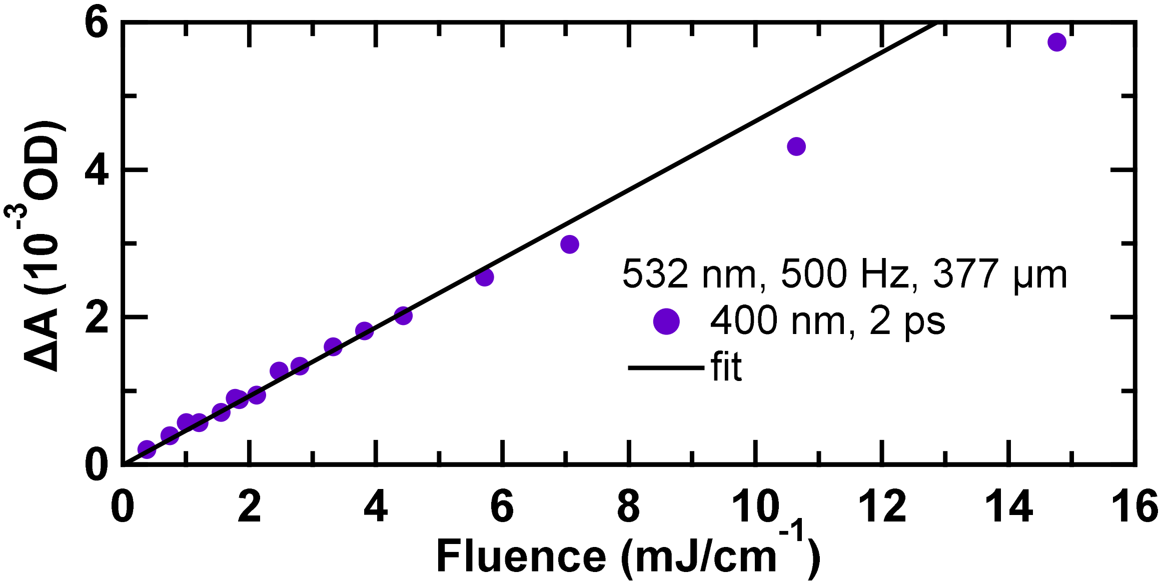

Figure S12: Static UV-vis spectrum before and after UV-vis OTA experiment.

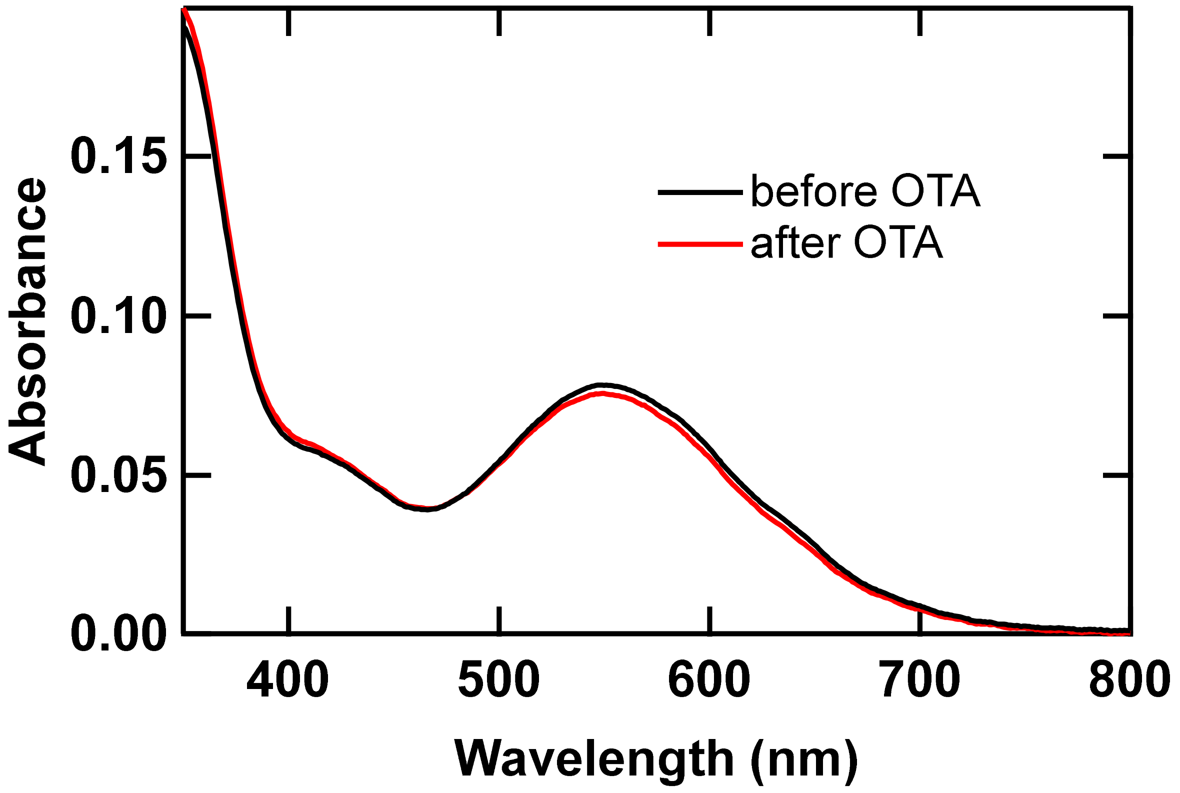

3.2 Target Analysis

Below, we show the OTA data sets and their kinetic fits. Kinetic traces of the OTA data were globally fit to a 3-component sequential model, as shown in Equation S4. The solutions to the rate equations were convoluted with a gaussian instrument response function.

Equation S4 $$A \xrightarrow{\tau_1} B \xrightarrow{\tau_2} C \xrightarrow{\tau_3} \text{ground state}$$

To improve the signal -to-noise, kinetic traces at adjacent probe wavelengths within a certain window were averaged together at the expense of spectral resolution. For example, in the UV-vis data set, the kinetic traces were spaced every 10 nm and averaged with the other kinetic traces within a ±5 nm window. The vis data were spaced by 5 nm and averaged in ±2.5 nm windows, the NIR data were spaced by 20 nm and averaged in ±10 nm windows, and the SWIR data were spaced by 50 nm and averaged in ±25 nm windows. For clarity, only a few select probe wavelengths are depicted in the kinetic traces below. The species-associated difference spectra (SADS) show the amplitude of the fit for a given component at each probe wavelength that was fit.

For the UV-vis, vis, and NIR data sets, the data points around time zero during pump-probe overlap were excluded from the fit due to the presence of coherent artifacts. For these experiments, the full width at half maximum (FWHM) of the instrument response function (IRF) was held constant. Fitting the solvent-only coherent artifact signal in the time domain with a gaussian function (Figure S13 & Figure S14) gave FWHM values of ~0.2 ps, so this value was used as the IRF parameter in the fits. The SWIR data, on the other hand, are essentially free of coherent artifacts, so we did not exclude any data points around time zero and let the IRF vary.

We note that the value of τ_1 obtained by the fits is very close to the IRF of the measurements, meaning the decay of this species is convoluted with the rise of the signal. In the case of the UV-vis, vis, and NIR data, where we excluded points during pump-probe overlap due to the presence of coherent artifacts, the τ_1 decay is hardly captured by the data. This situation is especially dire in the UV-vis and vis data, many data points around time zero are excluded and few data points related to τ_1 are actually fit. Therefore, the time constants and SADS for species A should be considered less accurate than those of species B and C.

The NIR and SWIR data do not show any signal at long time delays. In other words, the τ_3 = ~500 ps species is optically silent in this region. For consistency with the global fits of the UV-vis and vis data, this third component was still included in the NIR and SWIR global fits, but its time constant was held at a fixed value.

As shown in Figure S30, the dips in the SWIR transient spectra at 1725 nm occurs at the same wavelength as a minimum in the probe intensity, and are thus likely artifacts due to probe instability in that region.

Figure S13: Solvent-only scan of acetonitrile with UV-vis probe and 532 nm pump. Coherent artifact signal was fit with a gaussian to determine the full width at half maximum (fwhm) of the instrument response function. 0.2 mM [FcCc]PF_6 in MeCN, 0.2 cm path length, with 532 nm, 500 Hz, 4 mJ/cm^2 pump

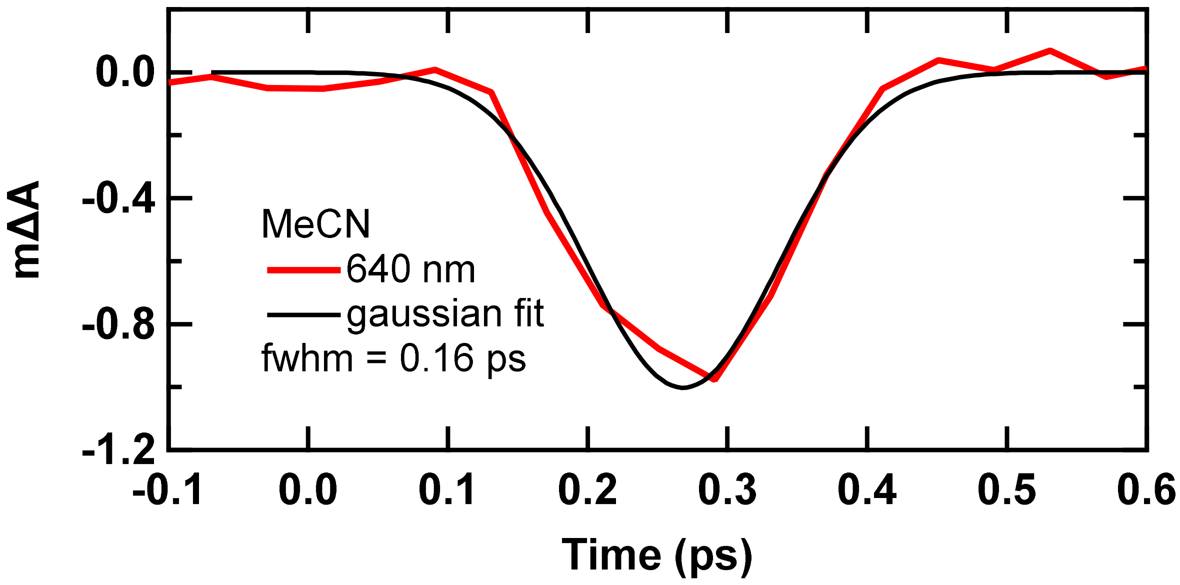

Figure S14: Solvent-only scan of acetonitrile, with vis probe and 515 nm pump. Coherent artifact signal was fit with a gaussian to determine the full width at half maximum (fwhm) of the instrument response function. The three data points around time zero that deviate from the gaussian shape of the signal were excluded from the fit. 1 mM [FcCc]PF_6 in MeCN, 0.1 cm path length, with 515 nm, 1 kHz, 1.7 mJ/cm^2 pump.

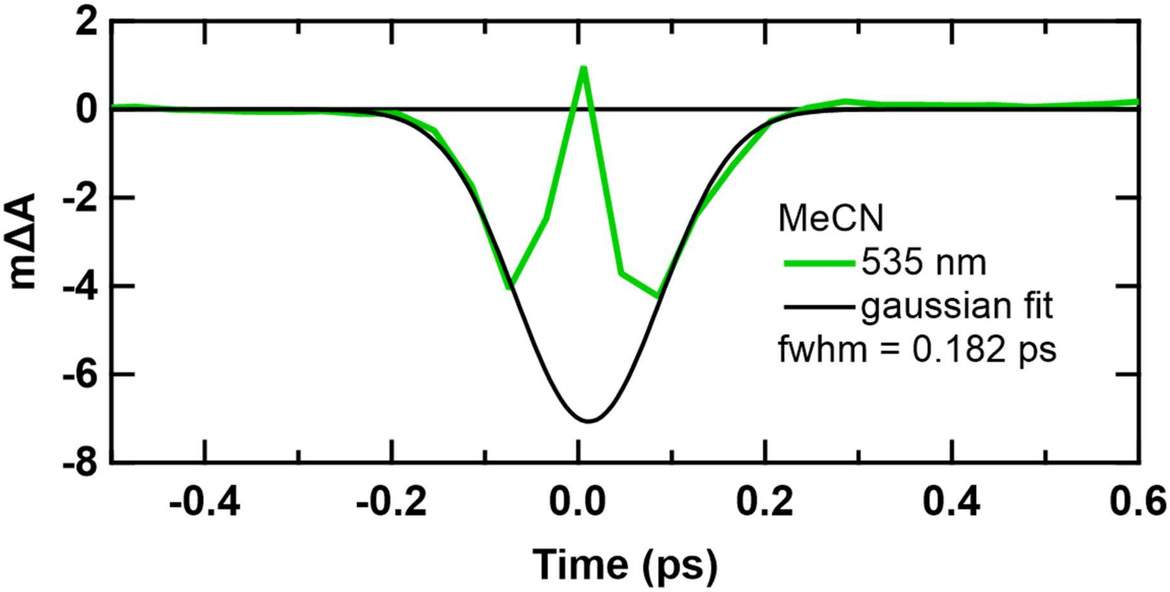

Figure S15: 2D map of UV-vis transient absorption data of 0.2 mM $[FcCc]PF_6$ in MeCN, 0.2 cm path length, with 532 nm, 500 Hz, 4 mJ/cm^2 pump.

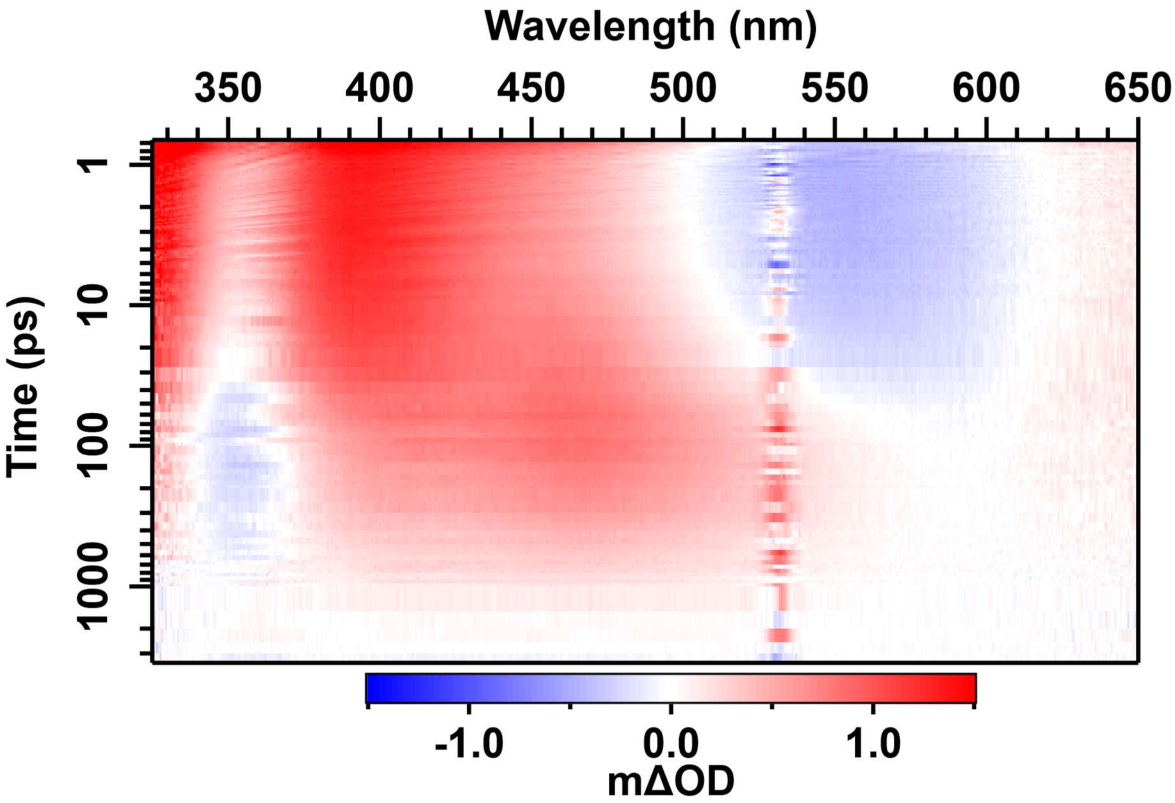

Figure S16: Kinetic traces (thick lines) and global fit (thin lines) at select wavelengths of UV-vis transient absorption data of 0.2 mM $[FcCc]PF_6$ in MeCN, 0.2 cm path length, with 532 nm, 500 Hz, 4 mJ/cm^2 pump.

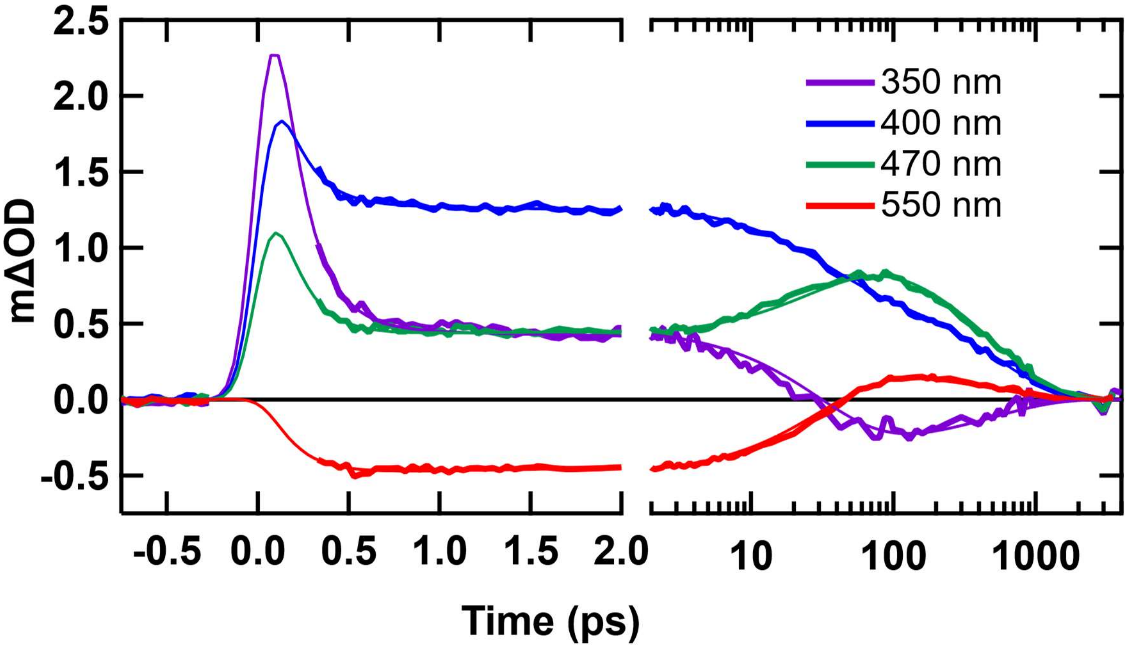

Figure S17: Spectral slices of UV-vis transient absorption data of 0.2 mM $[FcCc]PF_6$ in MeCN, 0.2 cm path length, with 532 nm, 500 Hz, 4 mJ/cm^2 pump.

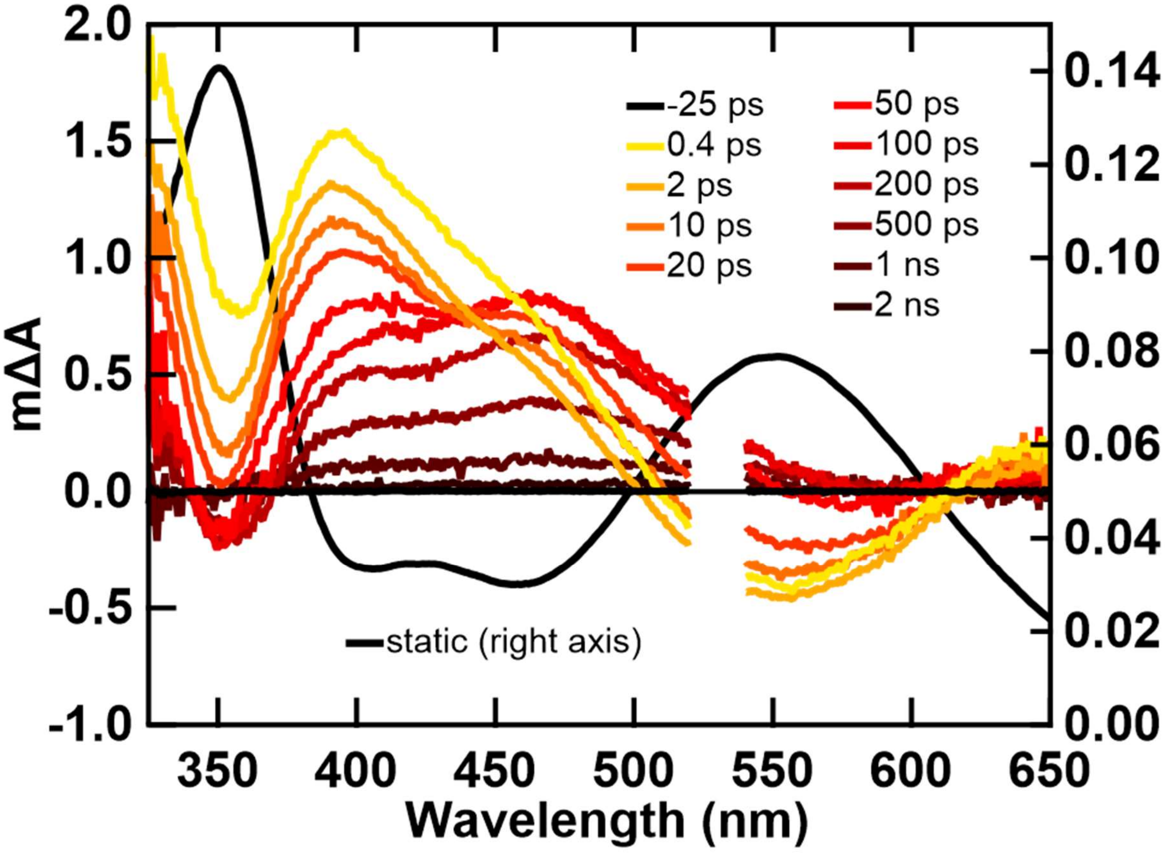

Figure S18: Species-associated decay spectra (SADS) from target analysis to a 3-component sequential model of UV-vis transient absorption data of 0.2 mM [FcCc]PF_6 in MeCN, 0.2 cm path length, with 532 nm, 500 Hz, 4 mJ/cm^2 pump.

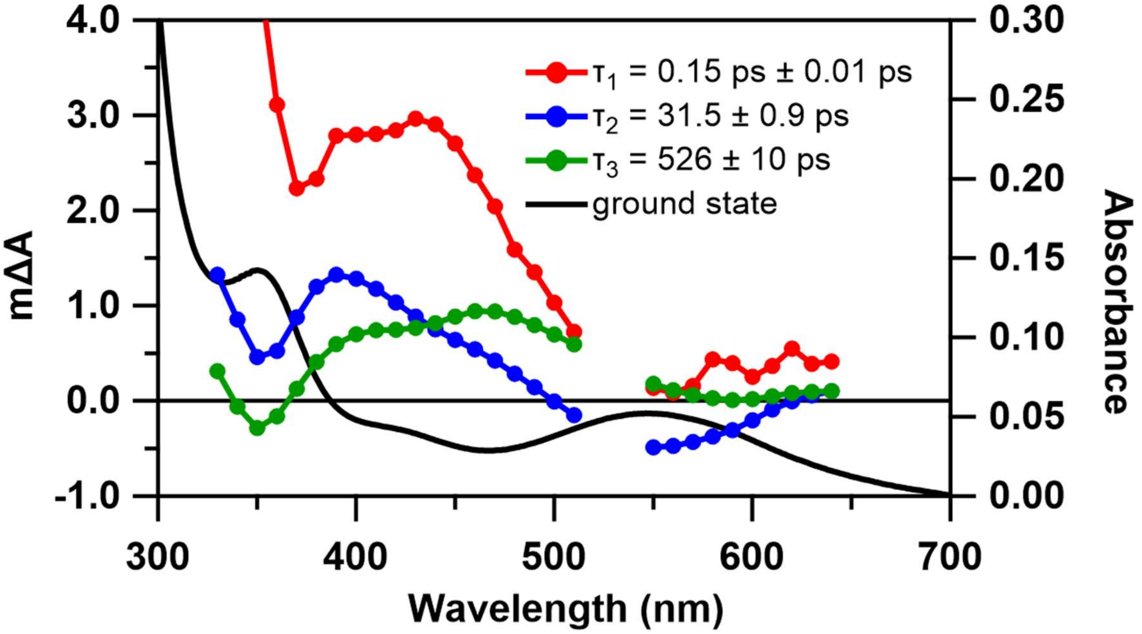

Figure S19: 2D map of vis transient absorption data of 1 mM $[FcCc]PF_6$ in MeCN, 0.1 cm path length, with 515 nm, 1 kHz, 1.7 mJ/cm^2 pump.

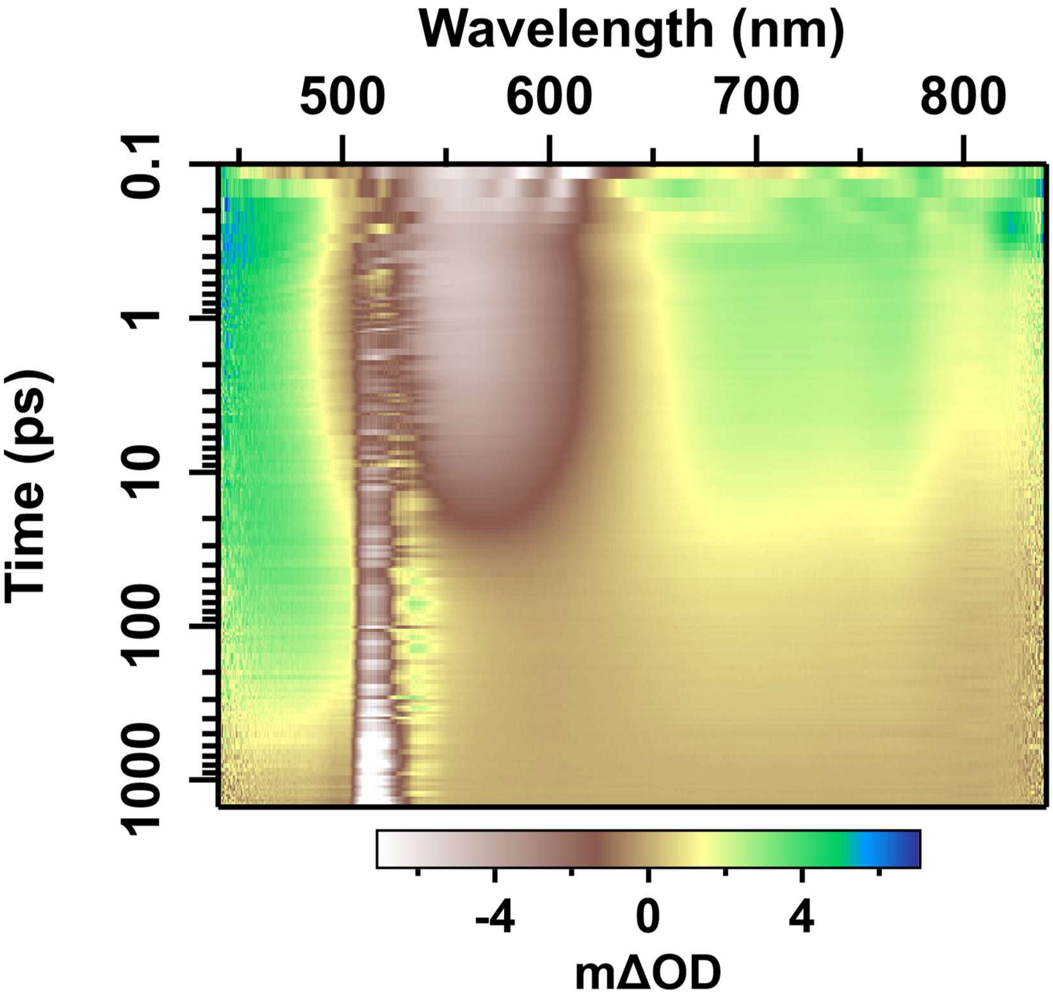

Figure S20: Kinetic traces (thick lines) and global fit (thin lines) at select wavelengths of vis transient absorption data of 1 mM [FcCc]PF_6 in MeCN, 0.1 cm path length, with 515 nm, 1 kHz, 1.7 mJ/cm^2 pump.

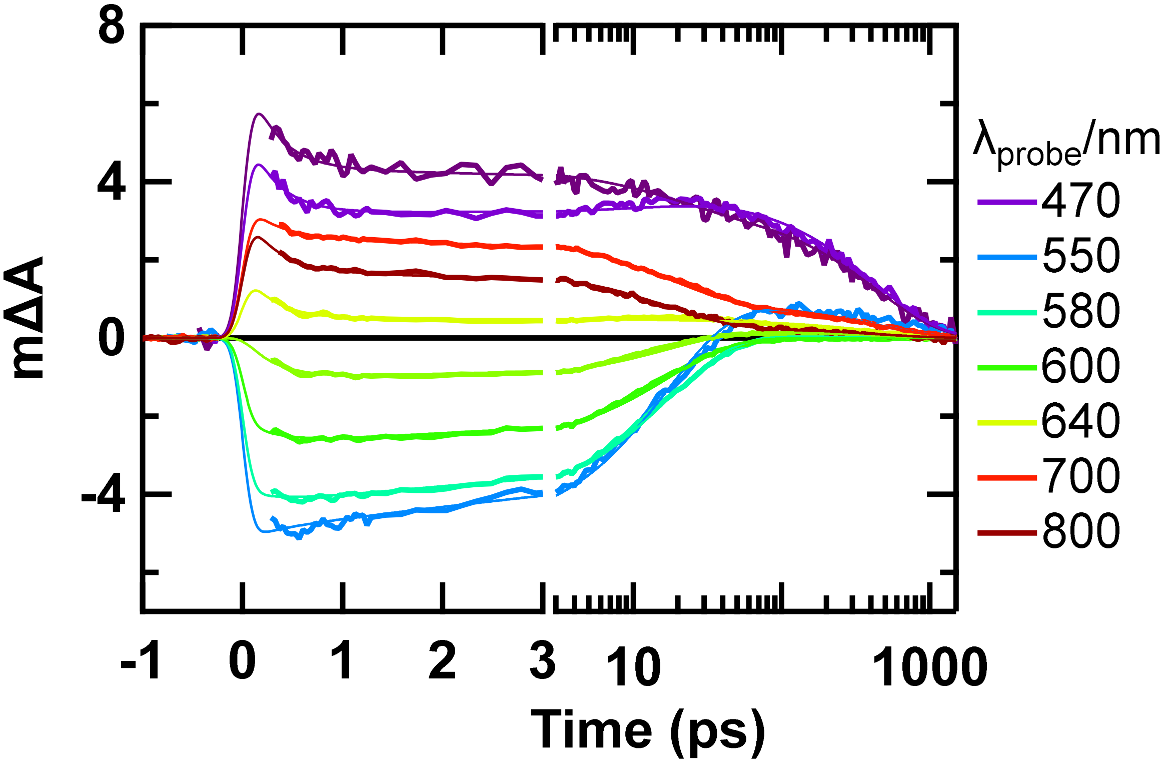

Figure S21: Spectral slices of vis transient absorption data of 1 mM $[FcCc]PF_6$ in MeCN, 0.1 cm path length, with 515 nm, 1 kHz, 1.7 mJ/cm^2 pump.

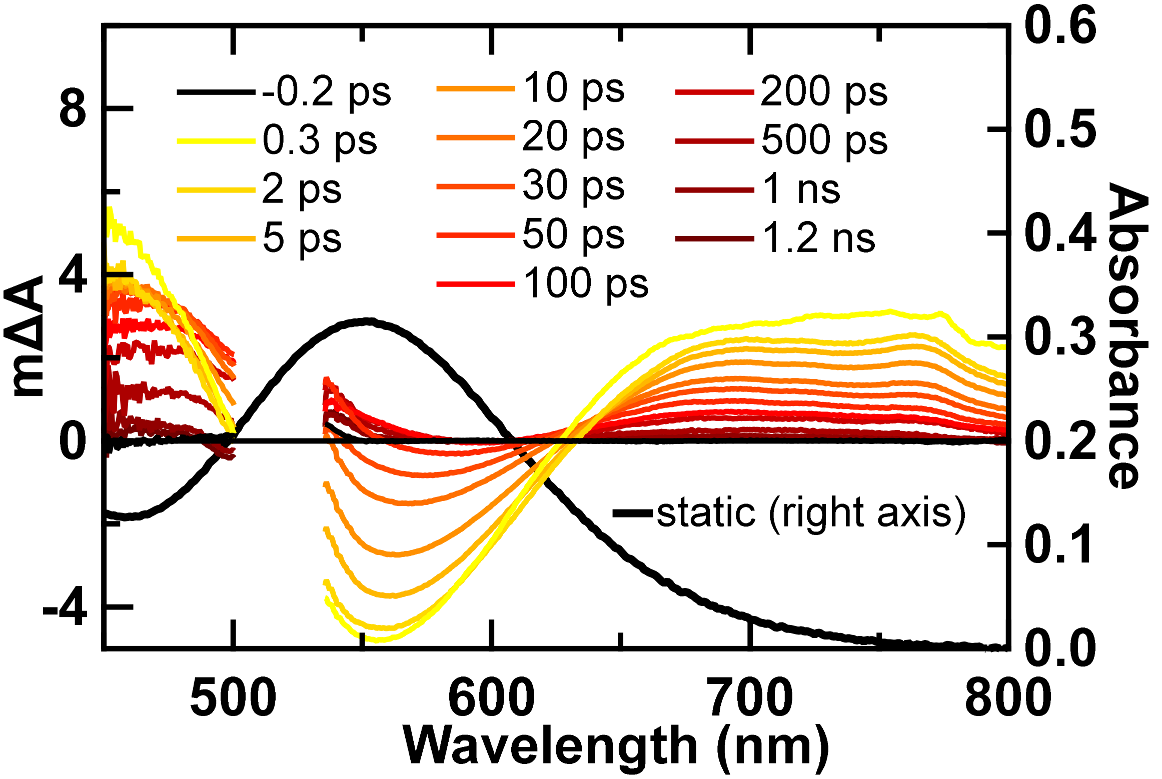

Figure S22: Species-associated decay spectra (SADS) from target analysis to a 3-component sequential model of vis transient absorption data of 1 mM $[FcCc]PF_6$ in MeCN, 0.1 cm path length, with 515 nm, 1 kHz, 1.7 mJ/cm^2 pump.

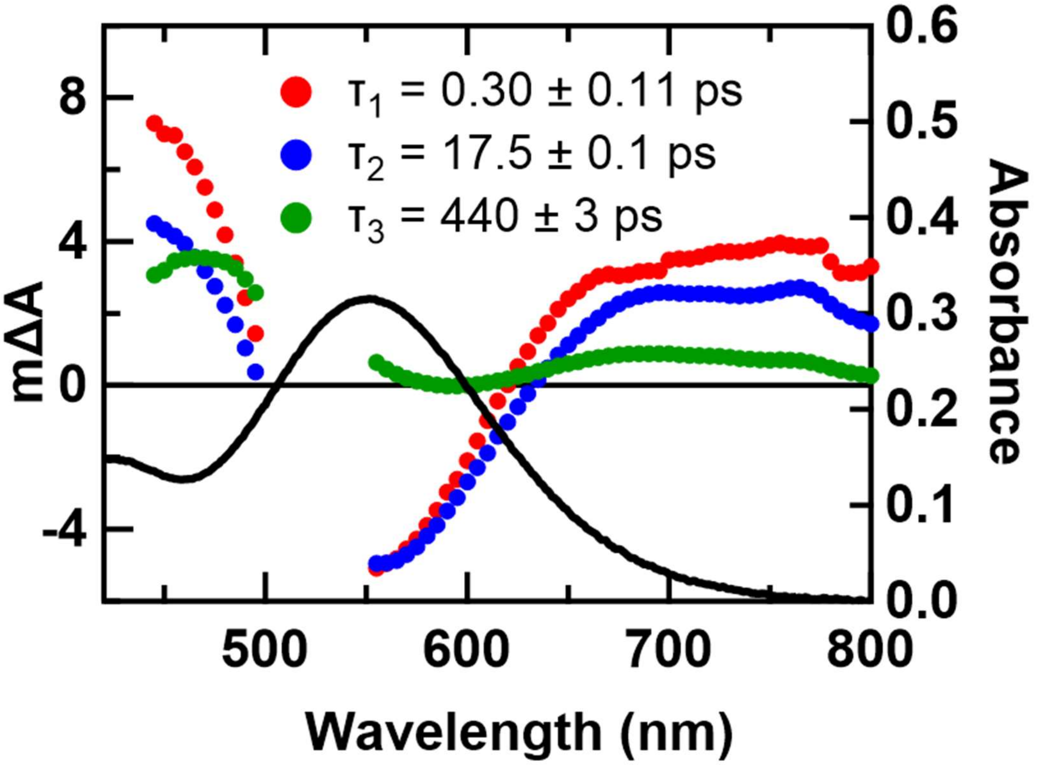

Figure S23: 2D map of NIR transient absorption data of 1 mM [FcCc]PF_6 in MeCN, 0.1 cm path length, with 515 nm, 1 kHz, 1.7 mJ/cm^2 pump.

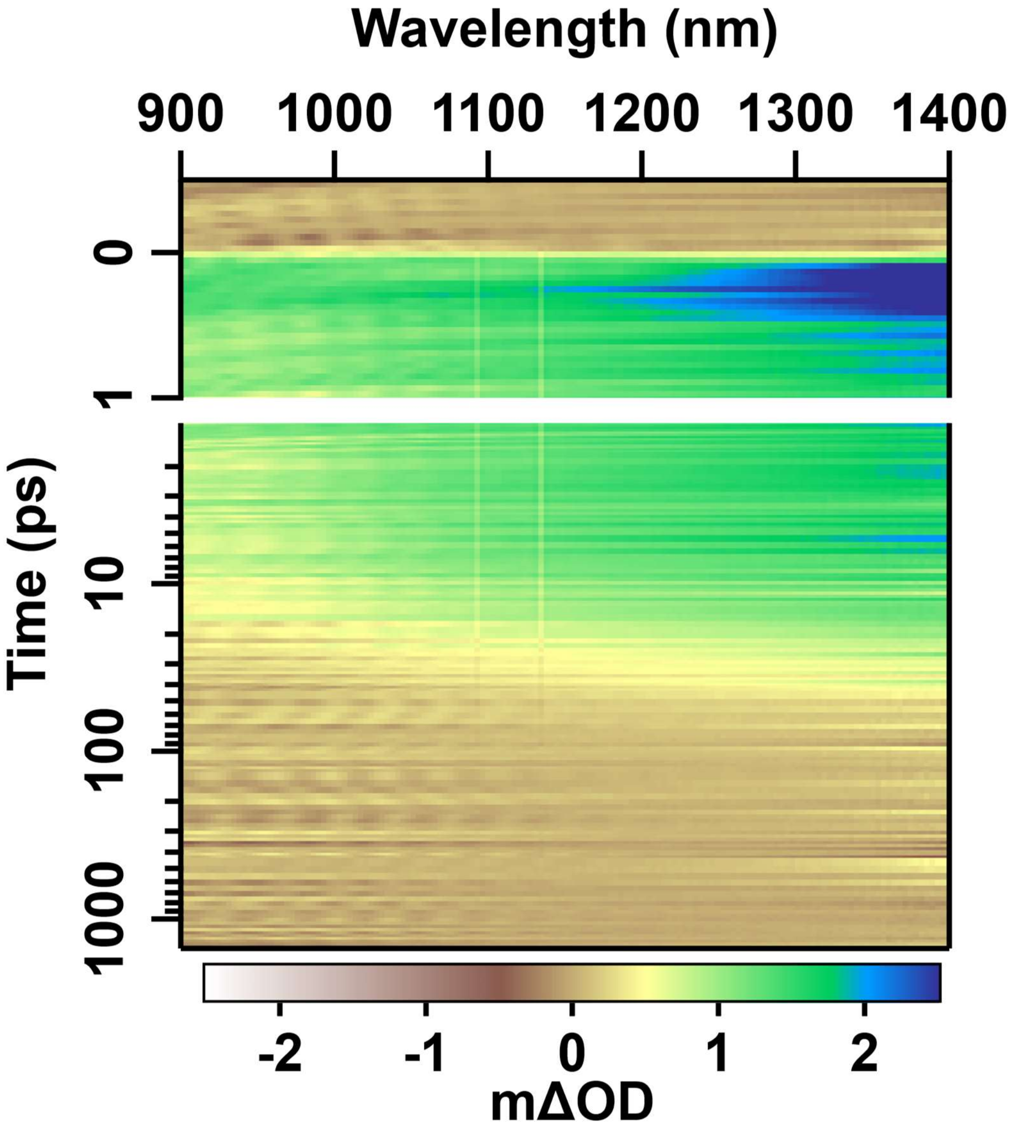

Figure S24: Kinetic traces (thick lines) and global fit (thin lines) at select wavelengths of NIR transient absorption data of 1 mM [FcCc]PF_6 in MeCN, 0.1 cm path length, with 515 nm, 1 kHz, 1.7 mJ/cm^2 pump.

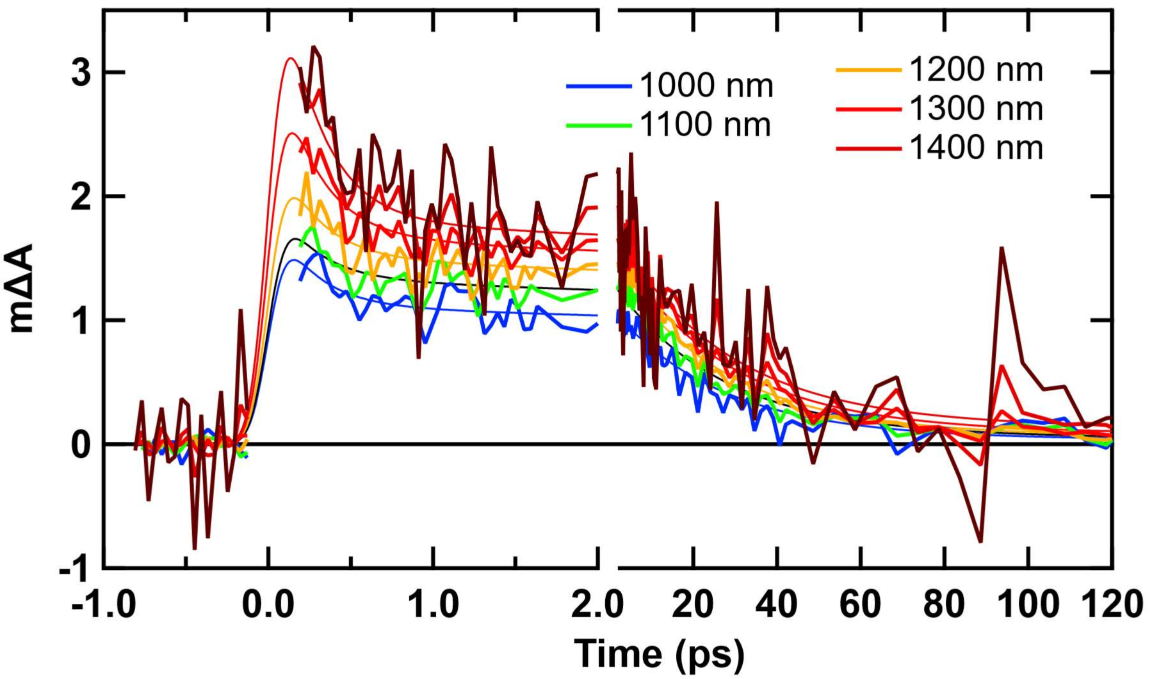

Figure S25: Spectral slices of NIR transient absorption data of 1 mM [FcCc]PF_6 in MeCN, 0.1 cm path length, with 515 nm, 1 kHz, 1.7 mJ/cm^2 pump.

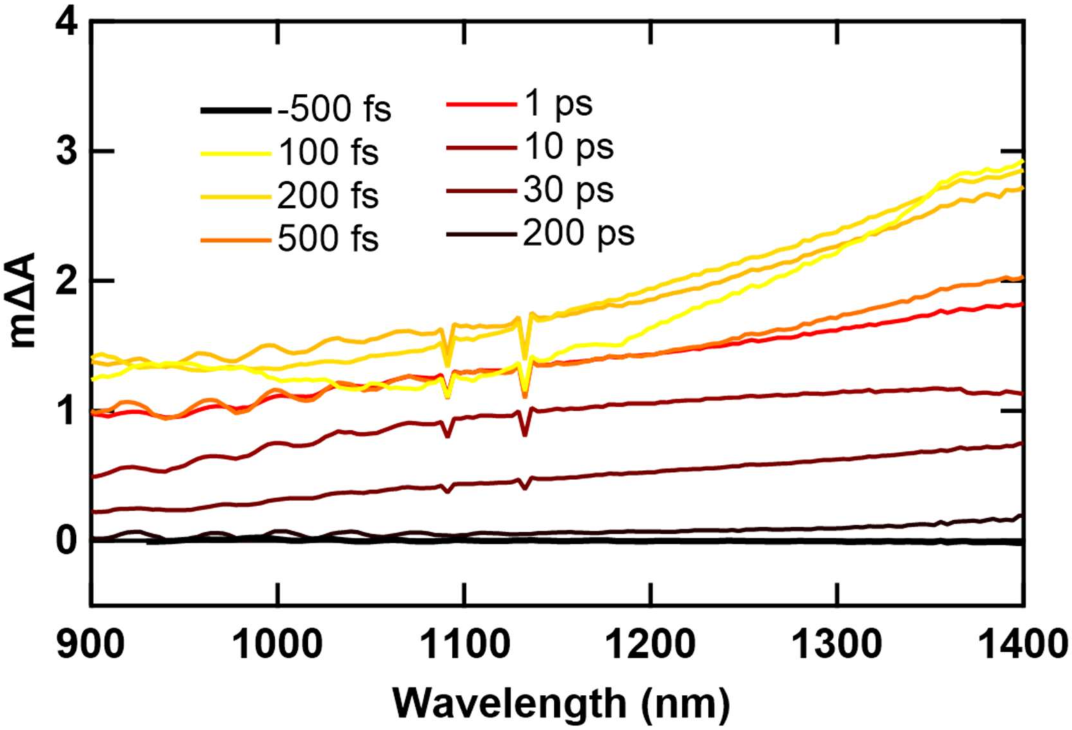

Figure S26: Species-associated decay spectra (SADS) from target analysis to a 3-component sequential model of NIR transient absorption data of 1 mM $[FcCc]PF_6$ in MeCN, 0.1 cm path length, with 515 nm, 1 kHz, 1.7 mJ/cm^2 pump.

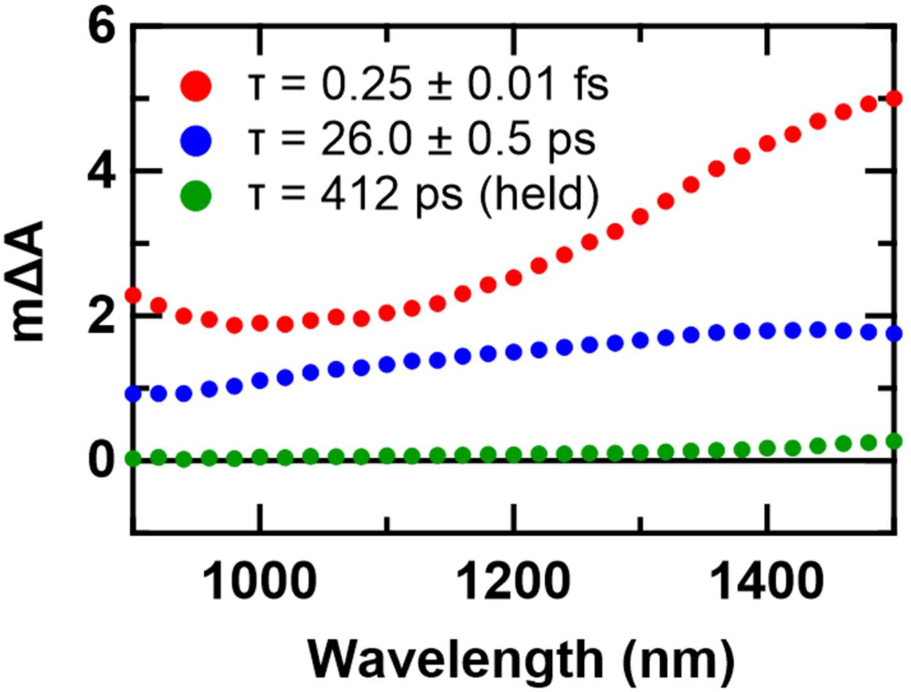

Figure S27: 2D map of SWIR transient absorption data of 1 mM $[FcCc]PF_6$ in MeCN, 0.1 cm path length, with 515 nm, 1 kHz, 3.3 mJ/cm^2 pump.

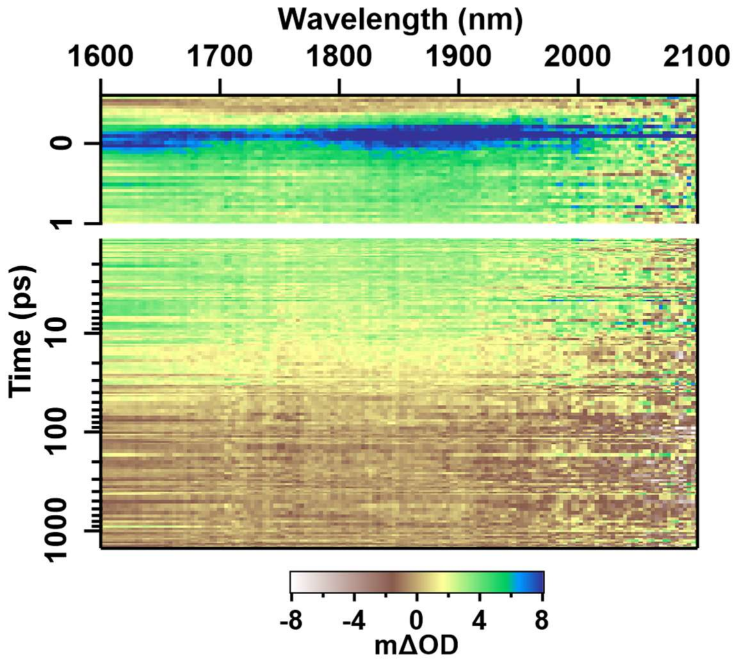

Figure S28: Kinetic traces (thick line) and global fit (thin line) at select wavelength of SWIR transient absorption data of 1 mM [FcCc]PF_6 in MeCN, 0.1 cm path length, with 515 nm, 1 kHz, 3.3 mJ/cm^2 pump.

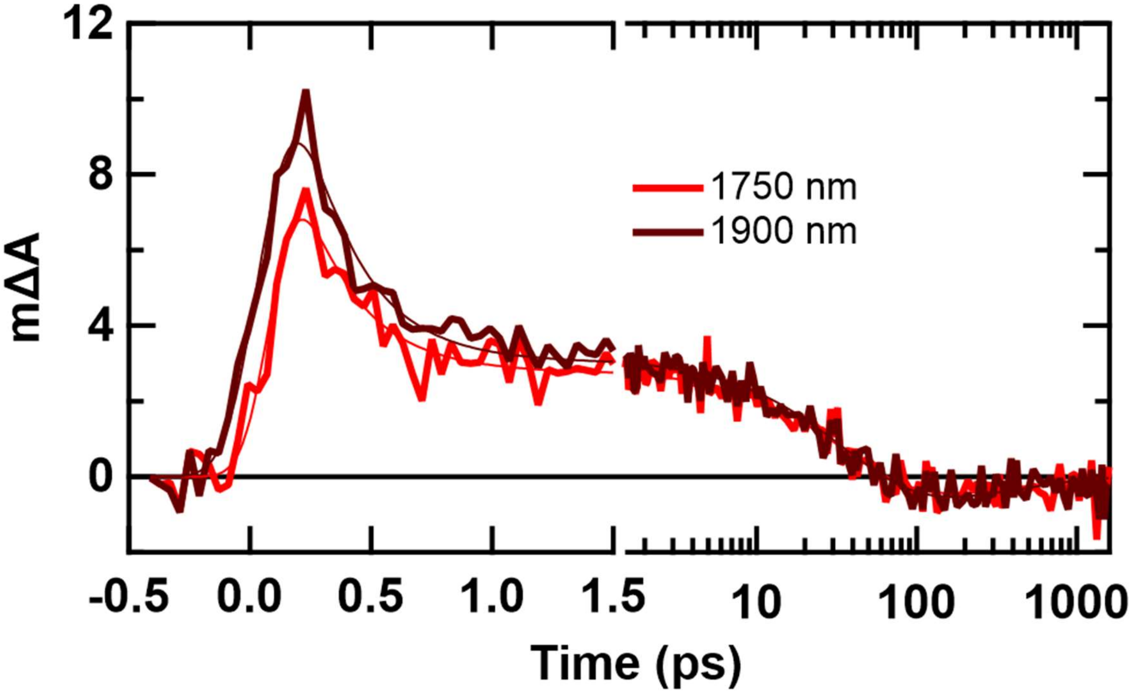

Figure S29: Spectral slices of SWIR transient absorption data of 1 mM $[FcCc]PF_6$ in MeCN, 0.1 cm path length, with 515 nm, 1 kHz, 3.3 mJ/cm^2 pump.

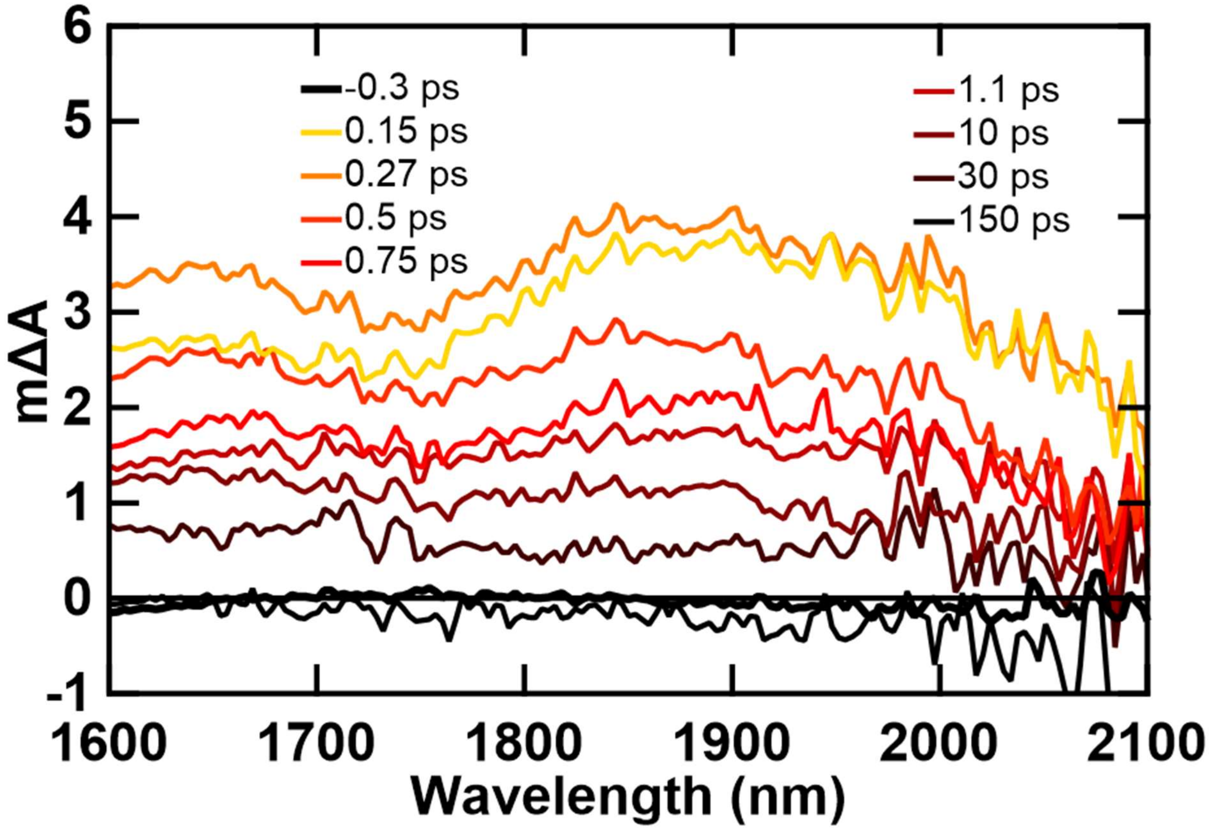

Figure S30: Spectral slices of SWIR transient absorption data of 1 mM $[FcCc]PF_6$ in MeCN, 0.1 cm path length, with 515 nm, 1 kHz, 3.3 mJ/cm^2 pump. Probe spectrum is shown for comparison. The dip in the transient spectra at 1725 nm are likely an artifact caused by instabilities of the probe at the minimum of intensity at the same wavelength.

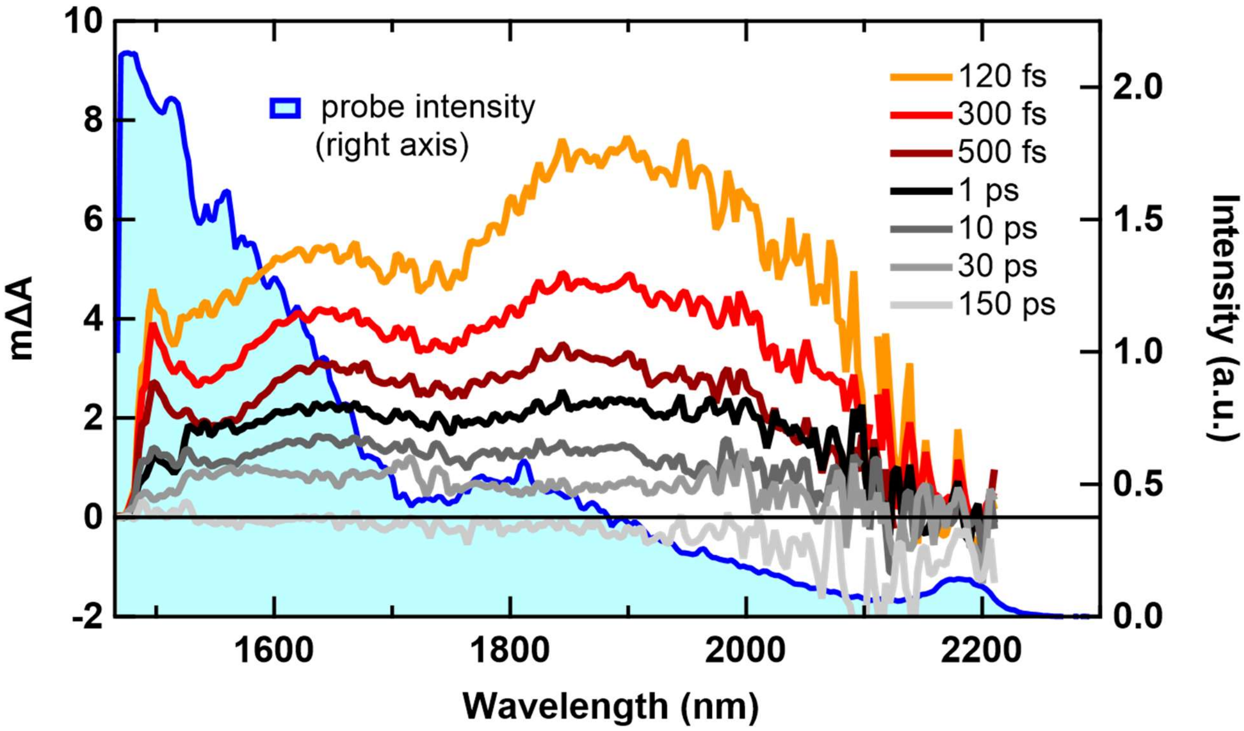

Figure S31: Species-associated decay spectra (SADS) from target analysis to a 3-component sequential model of SWIR transient absorption data of 1 mM $[FcCc]PF_6$ in MeCN, 0.1 cm path length, with 515 nm, 1 kHz, 3.3 mJ/cm^2 pump.

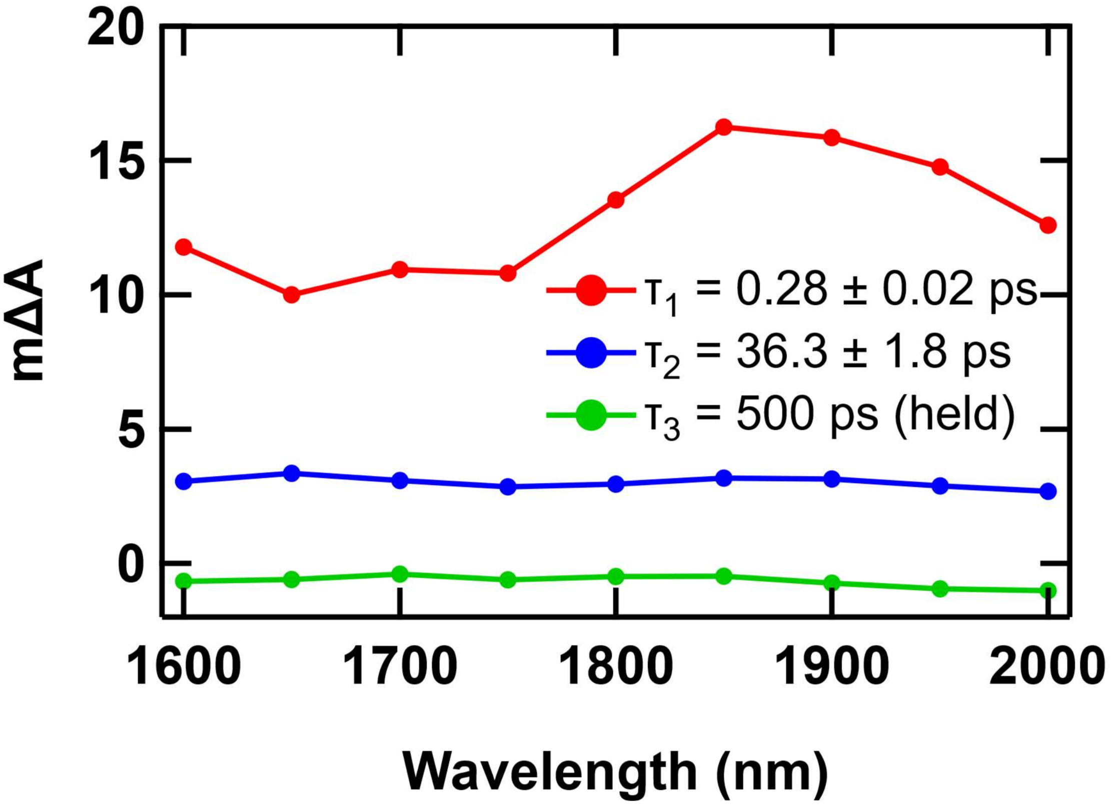

3.3 Comparison of Signal Amplitudes

The vis, NIR, and SWIR experiments were performed on the same setup with the same concentration of 1 mM and same sample path length of 1 mm, which allows for direct comparison of their signal amplitudes. The only difference in experimental conditions was that vis and NIR experiments were performed at a pump fluence of 1.7 mJ/cm^2, while the SWIR experiment was performed at 3.3 mJ/cm^2 to improve signal-to-noise. Therefore, in Figure 5 of the Main Text, the SWIR spectral traces were scaled by a factor of 0.6 to account for the increased pump fluence.

The UV-vis data, on the other hand, were collected on a different setup, which complicates comparison of signal magnitudes with the vis/NIR/SWIR experiments. The UV-vis experiment was performed with a 532 nm pump at a fluence of 4 mJ/cm^2 and 0.2 mM of sample in a 2 mm sample cell. In principle, accounting for the differences in concentration, path length, extinction coefficient, and fluence should allow for a comparison of the signal strengths. However, even after accounting for all of these factors, the UV-vis signal strength was still lower than that of the vis data set by a factor of 10 (Figure S32). Fortunately, the UV-vis and vis data sets overlap spectrally, which allowed us to normalize the signal magnitude of the UV-vis experiment to that of the vis experiment. The UV-vis spectral traces in Figure 5 of the Main Text were thus scaled by a factor of 10 to match the amplitude of the 550 nm bleach signal at 2 ps in the vis data set.

The UV-vis experiment was performed on a different instrument, which used the 532 nm output of an OPA at a fluence of 4 mJ/cm^2 as the pump pulse and a 2 mm sample cell. Even after accounting for the differences in experimental conditions, direct comparison of the signal magnitude from the UV-vis experiment with those of the vis/NIR/SWIR experiments was not possible. The UV-vis signal was lower than the vis signal by a factor of 10, possibly due to reduced spatial overlap of the pump and probe pulses. Thankfully, these two experiments overlapped spectrally in the 450-650 nm range. Therefore, we used the transient signal at the bleach feature at 550 nm at 2 ps to normalize the UV-vis experiment to the vis experiment.

Figure S32: Comparison of signal magnitudes of UV-vis and vis OTA data at 2 ps. The UV-vis data has been scaled to account for differences in concentration, path length, fluence, and extinction coefficient.

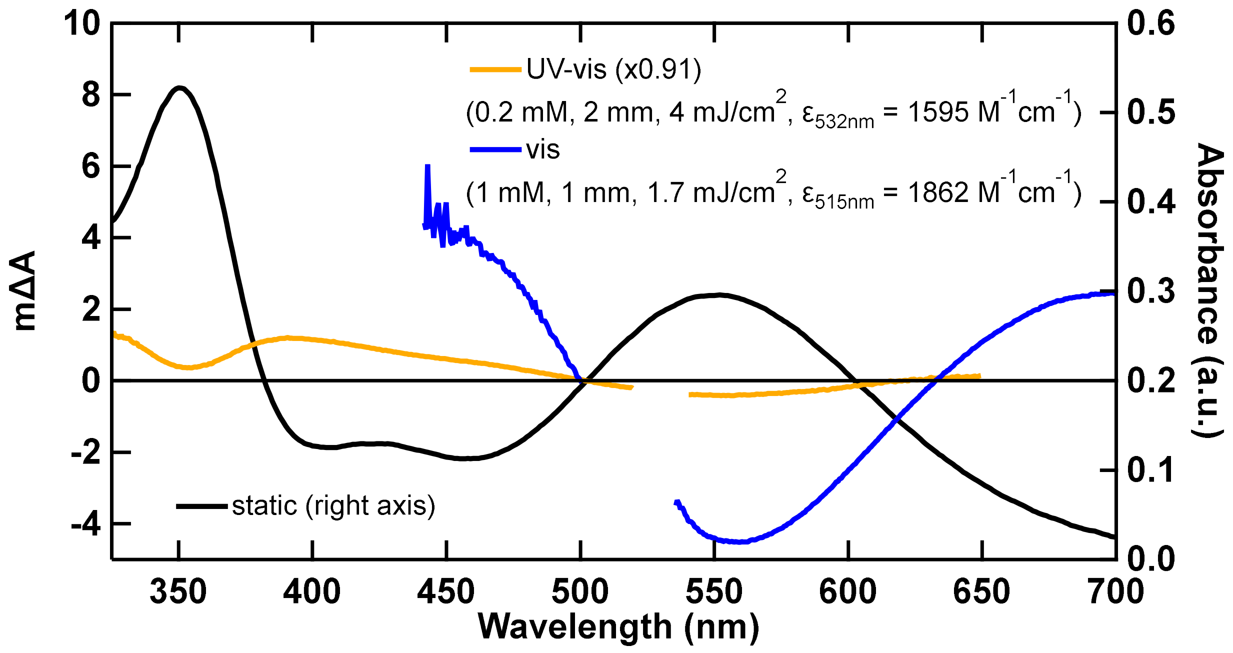

Figure S33: Comparison of signal magnitudes of UV-vis and vis OTA data at 2 ps after normalizing the UV-vis data by a factor of 10 to match the intensity of the bleach feature at 550 nm in the vis data set.

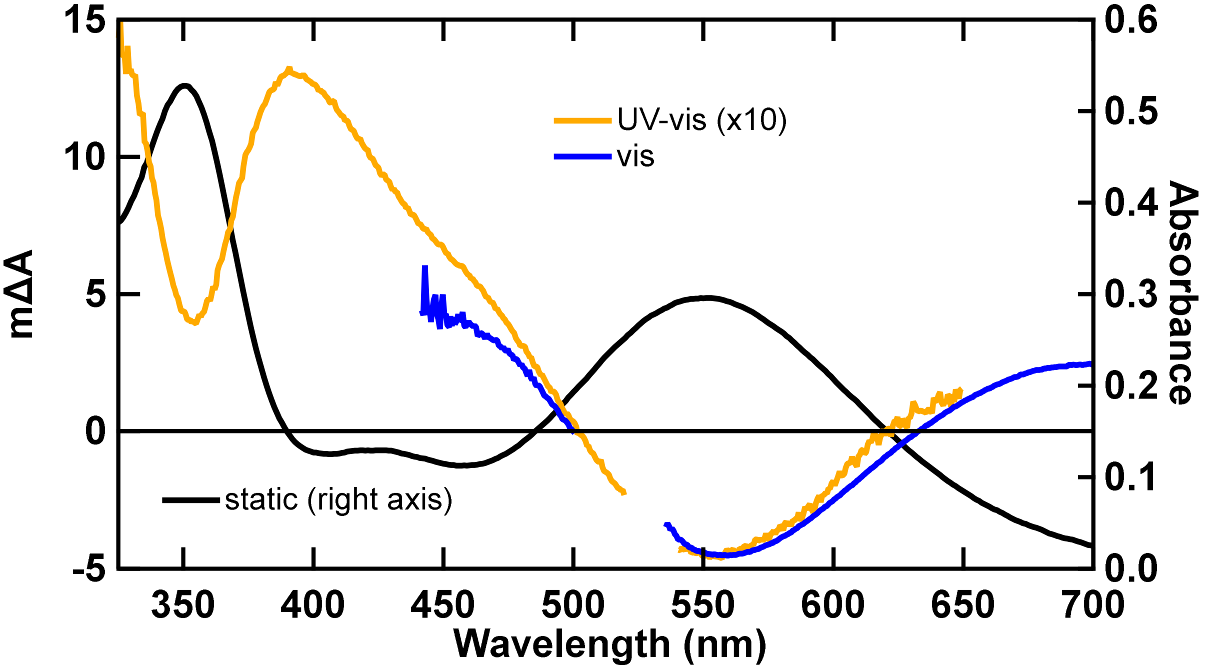

Figure S34: Spectral slices of NIR and SWIR OTA data. SWIR data have been scaled by 0.6 to account for higher fluence.

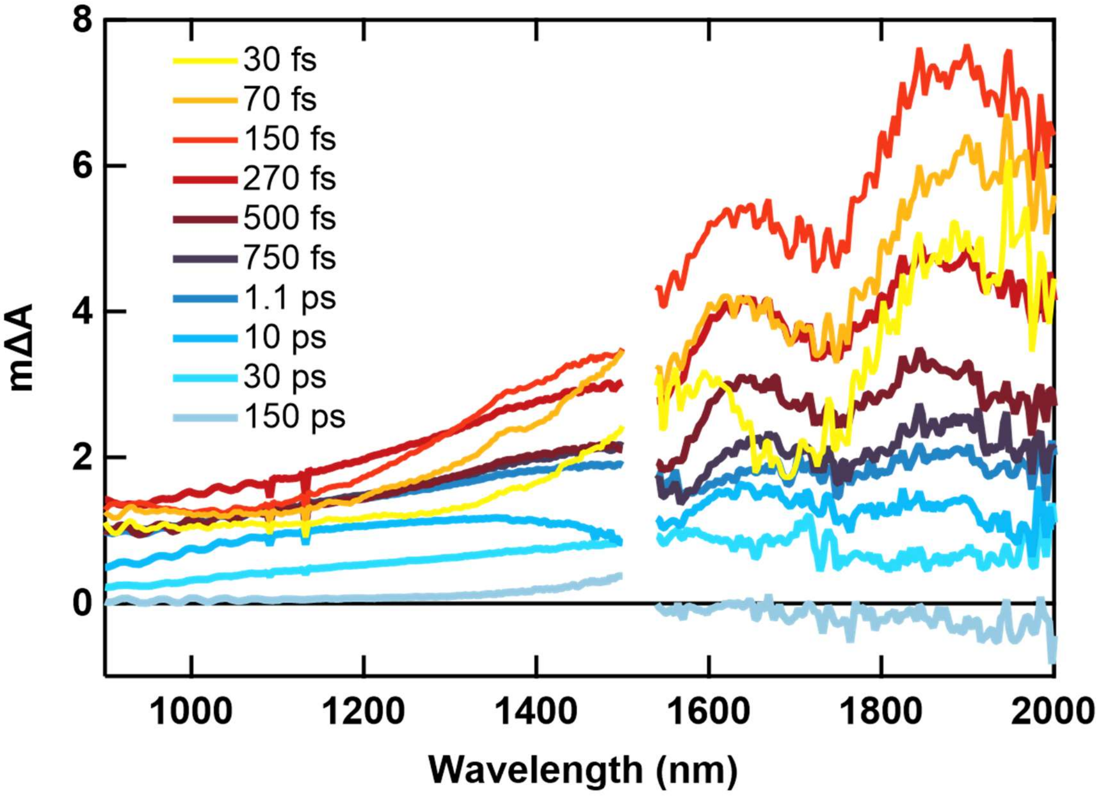

4 X-ray Transient Absorption Spectroscopy

X-ray transient absorption (XTA) spectroscopy measurements were performed at beamline 11ID-D of the Advanced Photon Source. We collected spectra in the X-ray absorption near edge structure (XANES) and extended X-ray absorption fine structure (EXAFS) regions. Spectra were collected in total fluorescence yield. The static X-ray absorption spectra were normalized to 1 above the edge. The transient signal magnitudes are on the same scale as the static spectra, i.e. an XTA signal of mΔA = 10 (ΔA = 0.01) corresponds to a transient absorption signal that is 1% of the edge jump of the static spectrum.

To test the monochromator calibration, we measured metallic foils of Fe and Co (Exafs Materials). Comparison of the edge energies (defined as the first peak in the derivative spectrum) to published values[17] (Figure 35 and Figure 36) show that the absolute energy scales are shifted by -0.6 eV at the Fe edge and by +0.8 eV at the Co edge. The spectra in the main text have been shifted accordingly; the spectra in the SI are uncorrected.

The pump laser wavelength was 515 nm. A pump laser fluence dependence of the XTA signal (Figure S37) showed linearity of the signal up to 20 mJ/cm^2, above which the signal saturated. In the OTA experiments, however, we performed a fluence dependence up to 16 mJ/cm^2 (Figure S11), which showed saturation of the OTA signal above 7 mJ/cm^2. Despite the more limited linear regime in the optical experiment, we chose to maximize the signal-to-noise during our XTA beamtime by measuring at the highest fluence possible in the linear regime based on our XTA fluence dependence. For that reason, we measured at a fluence of 20 mJ/cm^2. Due to the linearity of the XTA signal and the agreement in the lifetimes obtained from kinetic fitting, we are confident that we are probing the same dynamics in the OTA and XTA experiments.

The sample was a ~700 µm diameter cylindrical liquid jet of a recirculating solution of 5 mM [FcCc]PF_6. The sample was changed every ~12 hours. As shown the UV-vis spectra in Figure S38, the sample shows minimal damage during this time.

Figure 35: Static XANES and derivative spectrum of metallic Fe foil. Peak of first derivative is compared to reference value[1]: Bearden et al. Rev. Mod. Phys. 1967, 39 (1), 125–142.

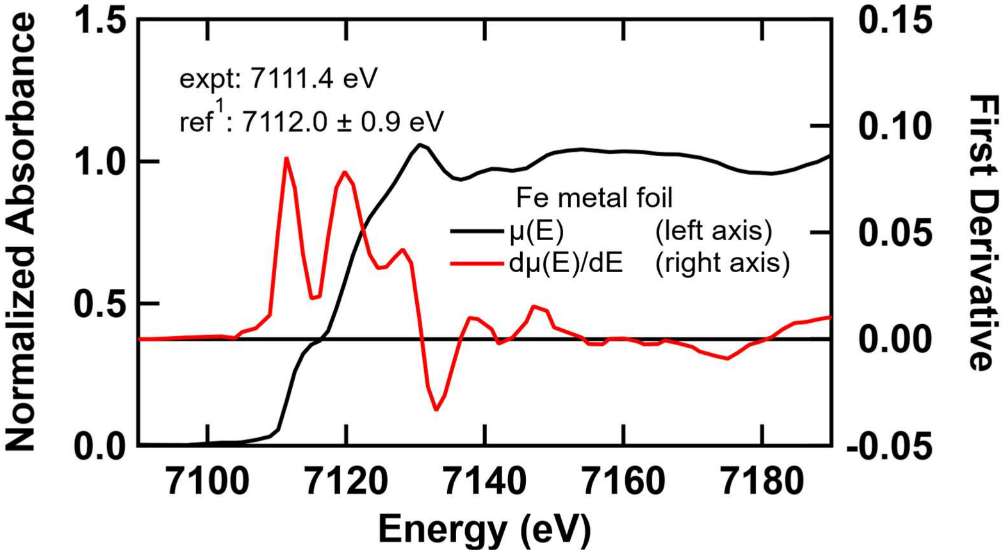

Figure 36: Static XANES and derivative spectrum of metallic Co foil. Peak of first derivative is compared to reference value[1]: Bearden et al. Rev. Mod. Phys. 1967, 39 (1), 125–142.

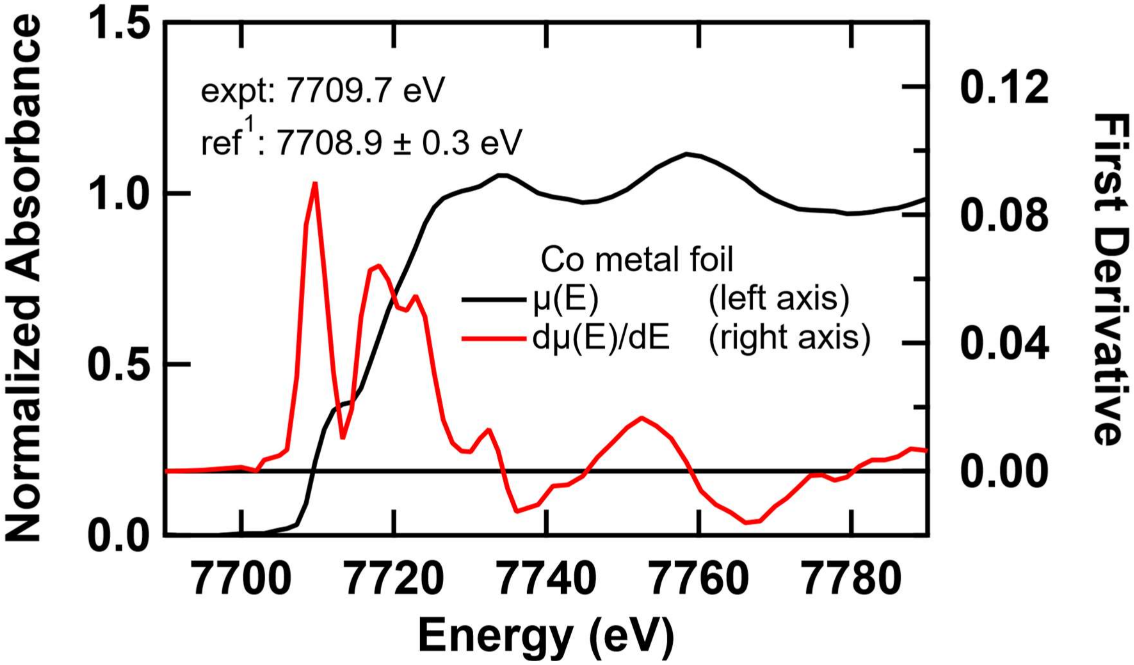

Figure S37: Fluence dependence of of Fe K edge XTA signal at the 7.124 keV ESA maximum. 5 mM [FcCc]PF_6 in MeCN, 700 µm cylindrical liquid jet, 515 nm, 3 kHz, 20 mJ/cm^2 pump focused to a 692 µm (1/e^2) diameter spot size.

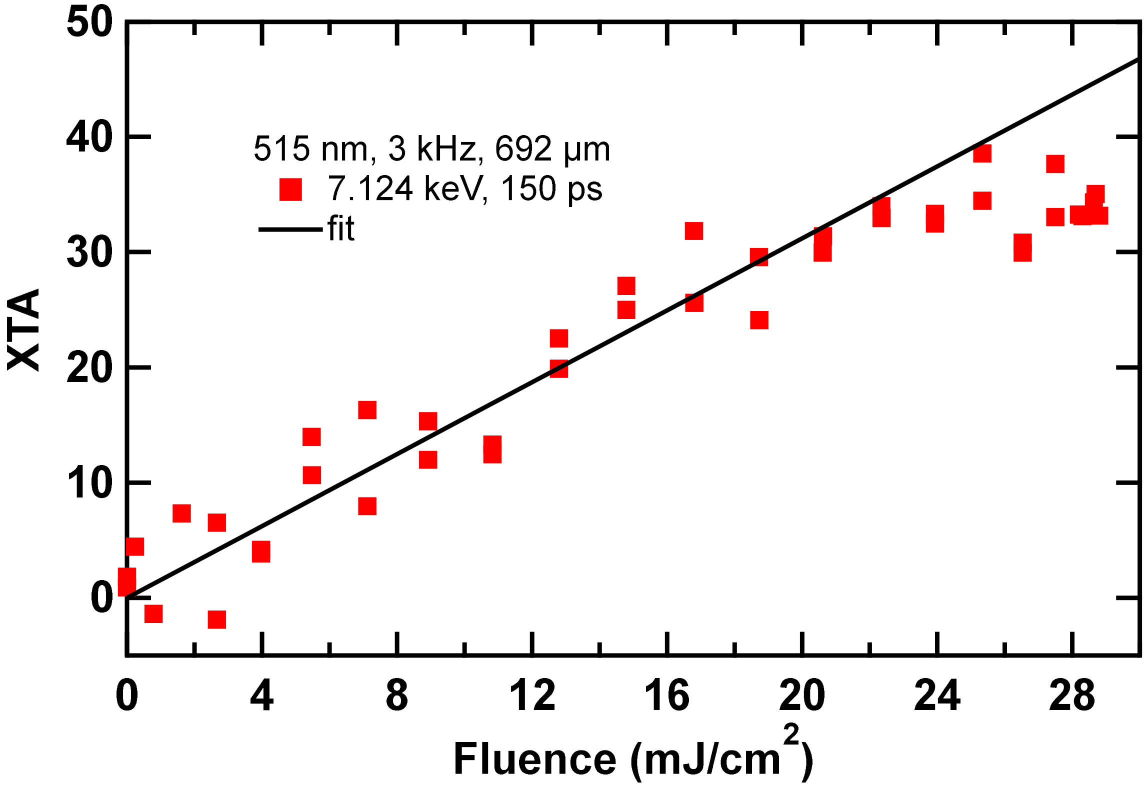

Figure S38: UV-vis spectrum of XTA sample before and after XTA measurement. The sample solution (5 mM [FcCc]PF_6 in acetonitrile) was diluted prior to the UV-vis measurement, and the spectra were normalized to the peak at 350 nm.

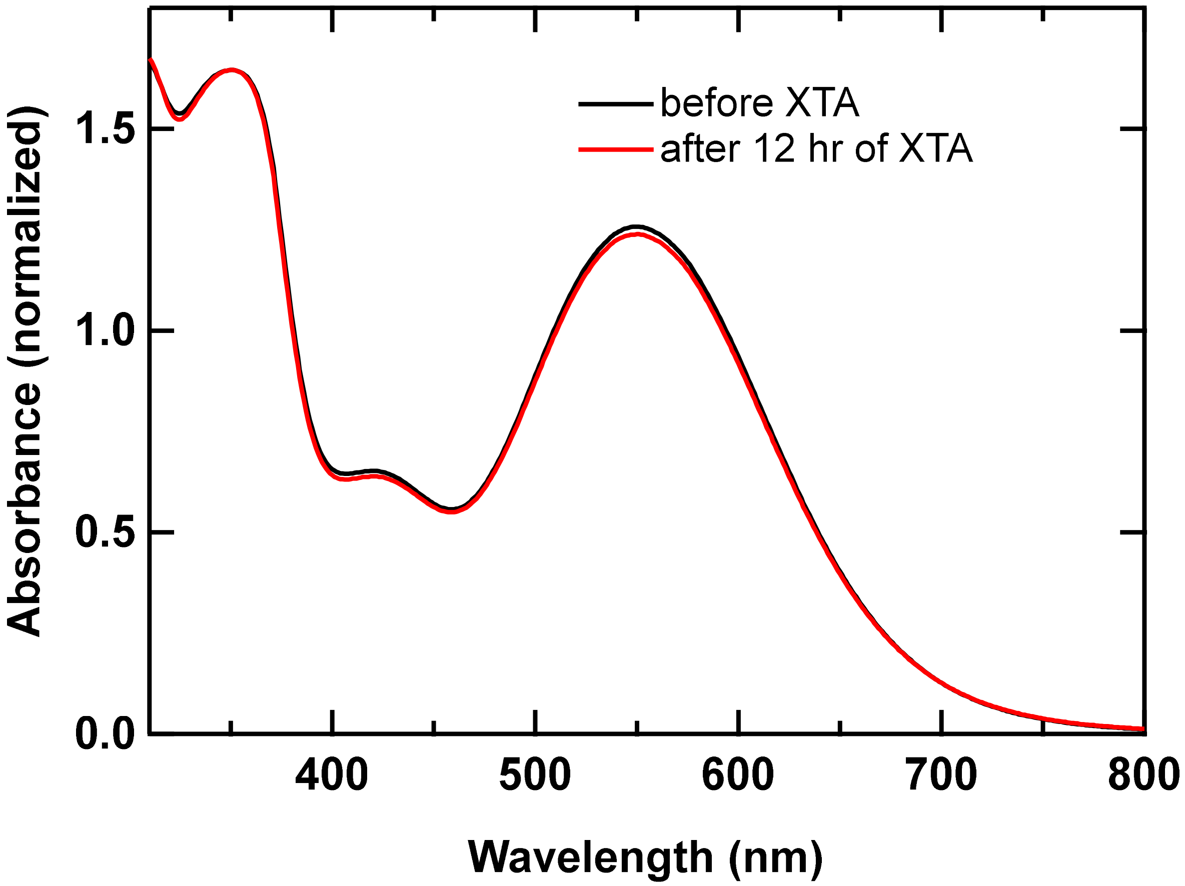

4.1 Data Processing

The XTA spectra were collected and averaged over multiple individual scans to build statistics. In regions of low signal strength, particularly the Fe pre-edge, Fe EXAFS, and Co XANES, it was necessary to perform a large number of scans to resolve a transient signal. The raw data, including every scan point at the Fe edge and Co edge, are shown in Figure S39 and Figure S40, respectively.

The individual scan points were binned according to their monochromator energies. At the Fe K edge, we utilized 0.4 eV bins in the the average of the pre-edge region (7.105 – 7.14 eV), 0.6 eV bins in the XANES region (7.012 eV – 7.138 eV) and 5 eV bins in the EXAFS region (7.138 eV – 7.612 eV). For the Co K-edge XANES (7.61 – 7.75 eV), we utilized 1 eV bins.

The XTA signal at a given monochromator energy is shown as a histogram in Figure S41. These histograms show that the transient absorption signal is well-described as a normally distributed random variable. Fitting the histograms to gaussian functions, we obtain standard deviations of 3.05 mΔA at the Co K-edge bleach and 5.04 mΔA at the Fe K-edge ESA. With the relatively large sample sizes at these energies ($N = 461$ and $N = 128$, respectively), these standard deviations should give a fair approximation of the standard deviation of the population, which characterizes the inherent uncertainties of the measurement. Errors in the XTA signal can be caused by fluctuations in the liquid jet, instability of the laser power/alignment, and many other factors. Despite this inherent error in the XTA signal (standard deviation of the population), it is possible to narrow down the uncertainty in the average XTA signal (mean of the population) by increasing the sample size. The uncertainty in the average XTA signal is known as the standard error of the mean, and from this quantity we are able to calculate confidence intervals.

Before averaging, the XTA spectra were background-subtracted by taking the mean of the pre-edge baseline points and subtracting that value from the entire spectrum. Then, for each energy bin, the average and standard deviation of the transient signal was calculated. A tolerance was defined as the number of standard deviations away from the mean that individual scan points would be considered outliers and rejected. Figure S42 shows the dependence of the outlier rejection tolerance on the final average and standard deviation. The number of data points rejected roughly follows the proportion expected for a normal distribution. We ultimately employed a tolerance of 3σ, which resulted in the rejection of 13 (0.26%) out of 4975 data points in the Fe K-edge XANES, 32 (0.64%) of 4985 data points in the Fe K-edge EXAFS, and 27 (0.27%) out of 10105 data points at the Co K-edge XANES. After rejecting the outlier points as defined by the tolerance, we calculated a final average, standard deviation, standard error of the mean, and (two-sided) 99.9% confidence interval of the data. The confidence interval is calculated by multiplying the standard error of the mean by the appropriate Student's t value for the number of data points averaged. For an infinite sample size, the 99.9% confidence interval is 3.090x the standard error of the mean.

As discussed above, the standard deviation reports on the uncertainty in the XTA signal inherent to the experiment (due to jet fluctuations, laser power fluctuations, etc.). On the other hand, the standard error of the mean and 99.9% confidence interval report on the uncertainty in the average

XTA signal. A comparison of error bars based on these quantities for the Fe K-edge transient EXAFS spectrum is shown in Figure S43-Figure S45. The standard deviation is relatively constant over the whole spectral range, despite the data points above 7.41 eV having significantly fewer numbers of scans that contribute to the average. On the other hand, the 99.9% confidence interval better reflects the uncertainty in the average XTA signal, which is minimized by increasing the sample size of the measurement. For this reason, we present our XTA spectra in the main text with error bars of ± the 99.9% confidence interval and use these errors in the χ^2 analysis used to evaluate the excited-state EXAFS fitting described in Section 4.4.

Figure S39: Individual scan points of Fe K-edge XTA spectrum at 150 ps. Each scan is represented by a different color. Static spectrum is shown in grey and plotted on the right axis. 5 mM [FcCc]PF_6 in MeCN, 700 μm cylindrical liquid jet, 515 nm, 3 kHz, 20 mJ/cm^2 pump.

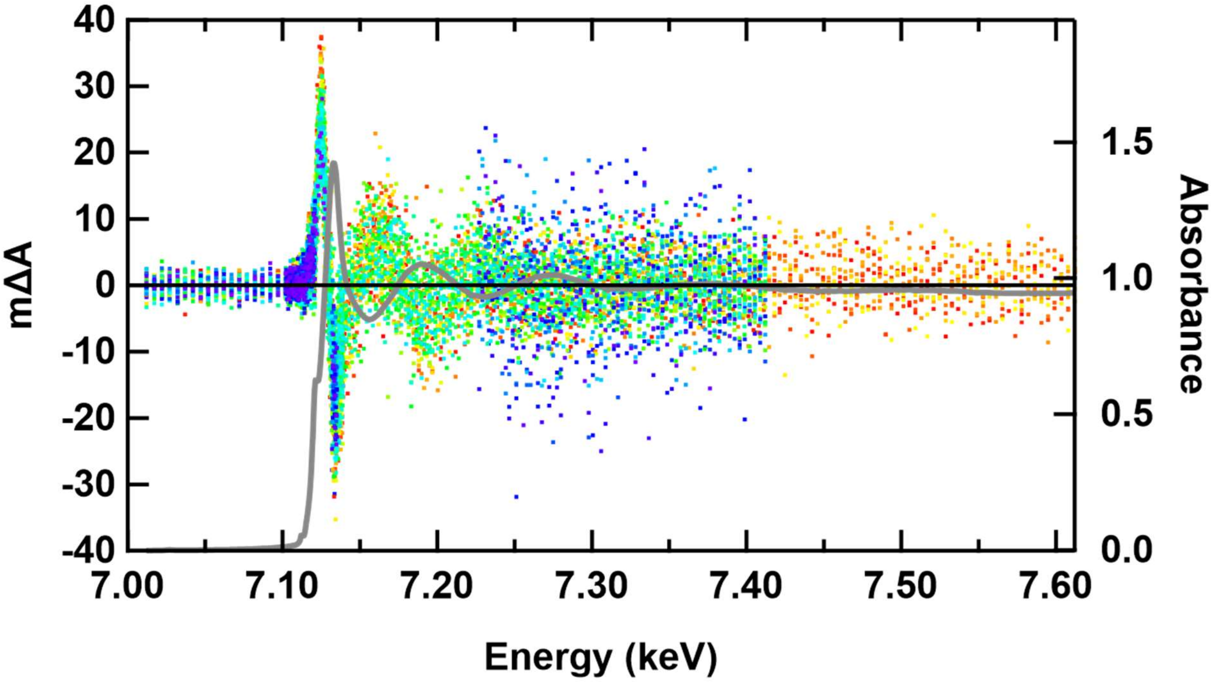

Figure S40: Individual scan points of Co K-edge XTA spectrum at 150 ps. Each scan is represented by a different color. Static spectrum is shown in grey and plotted on the right axis. 5 mM $[FcCc]PF_6$ in MeCN, 700 µm cylindrical liquid jet, 515 nm, 3 kHz, 20 mJ/cm^2 pump.

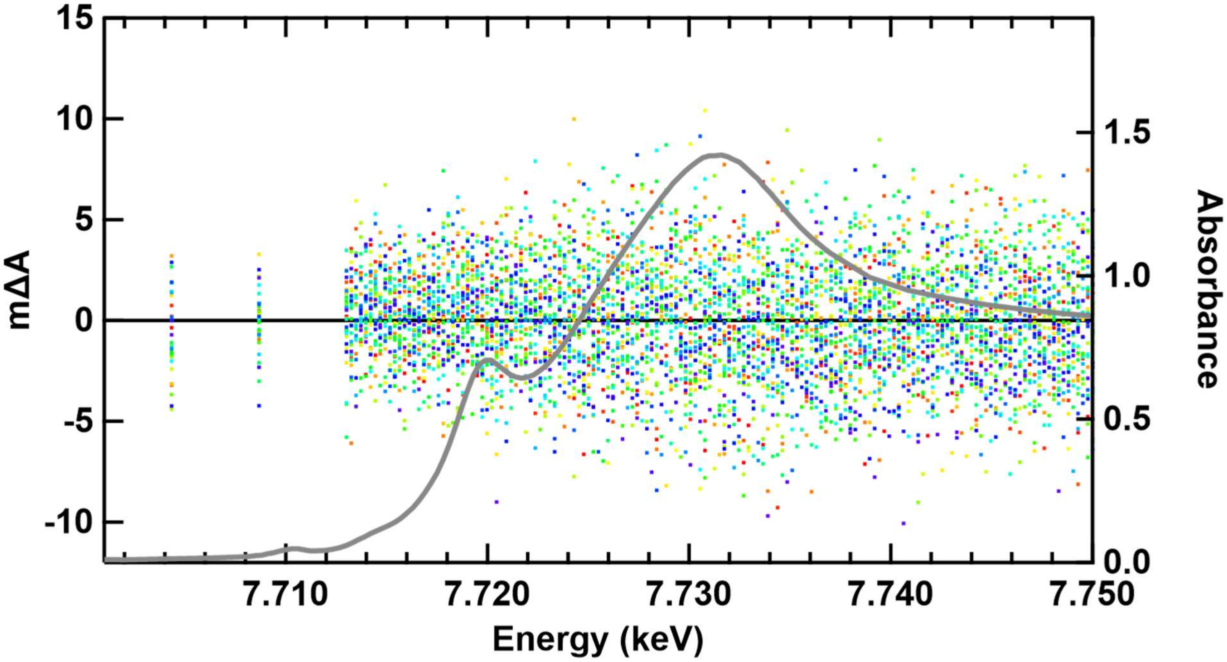

Figure S41: Histograms of the XTA signal at the Co K-edge bleach (7732.9 ± 1 eV) and Fe K-edge excited-state absorption (7124.8 ± 0.5 eV). The histograms were fit to gaussian functions characterized by the center point, x_0, and standard deviation, σ.

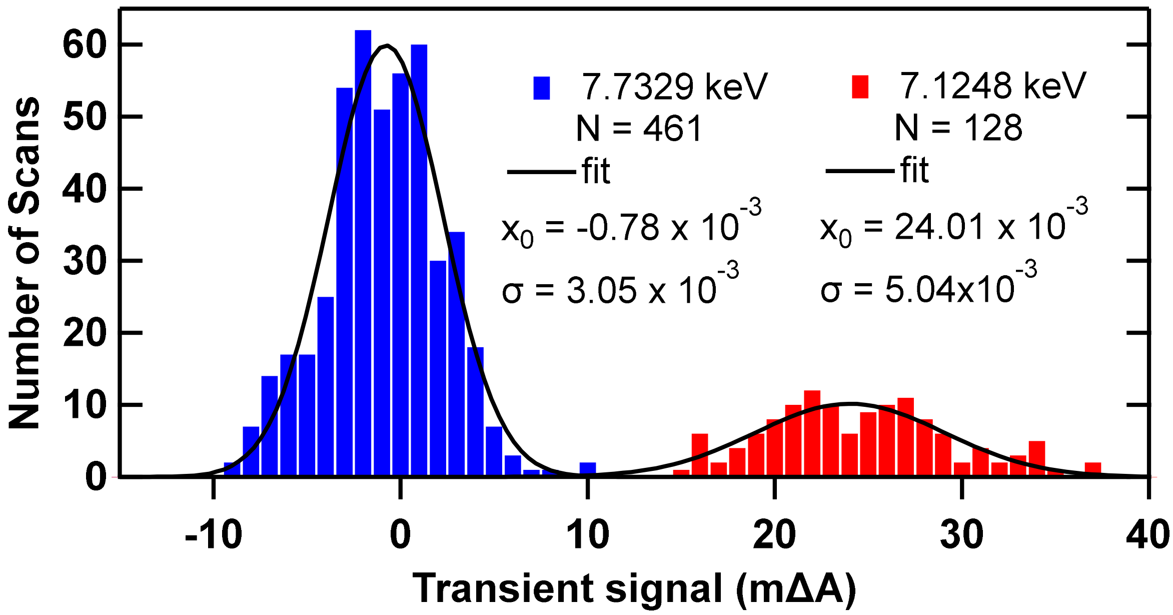

Figure S42: Effect of outlier rejection on Fe K-edge EXAFS spectra. The tolerance was defined as the number of standard deviations away from the mean that a data point had to be to be considered an outlier and rejected from the final calculation of the average and standard deviation.

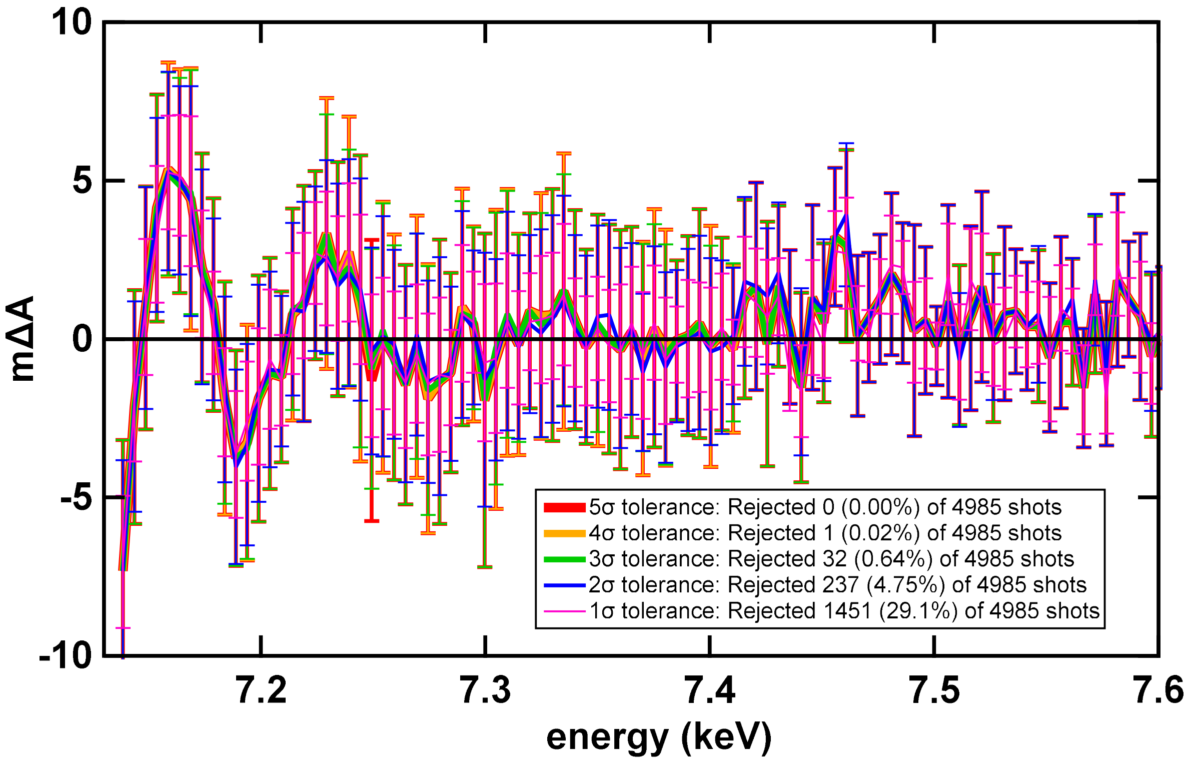

Figure S43: Comparison of the standard deviation and 99.9% confidence interval of the Fe K-edge transient EXAFS data at 150 ps. 5 mM [FcCc]PF_6 in MeCN, 700 μm cylindrical liquid jet, 515 nm, 3 kHz, 20 mJ/cm^2 pump.

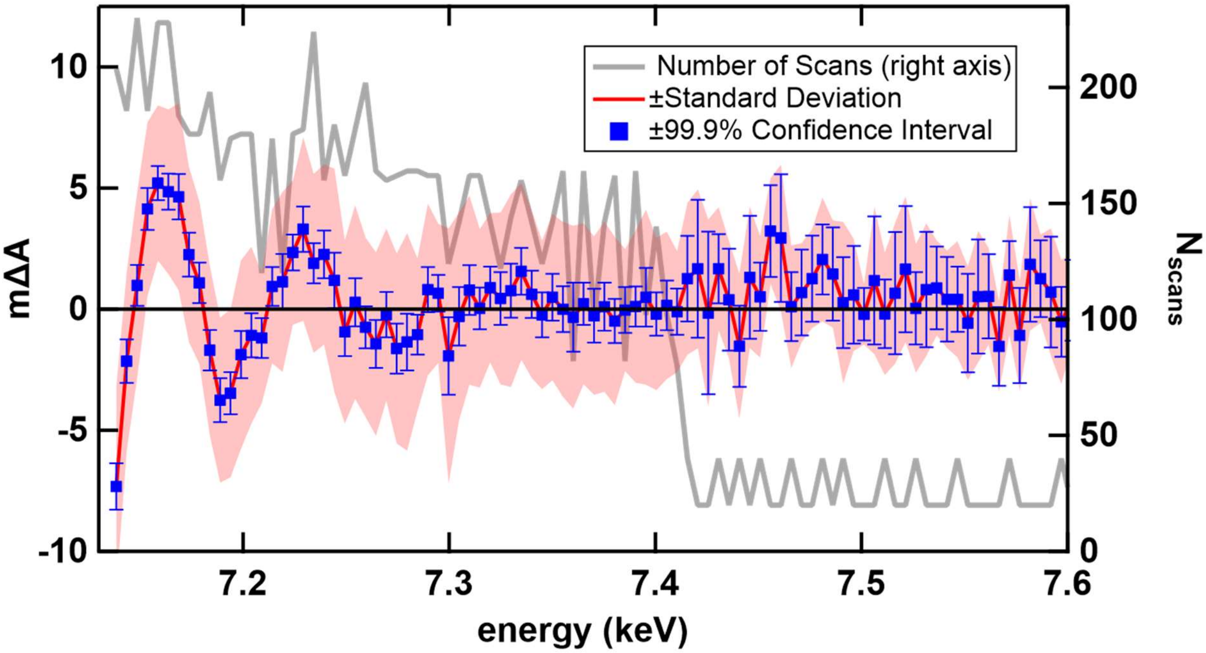

Figure S44: Comparison of the standard deviation and 99.9% confidence interval of the Fe K-edge transient XANES data at 150 ps. 5 mM $[FcCc]PF_6$ in MeCN, 700 μm cylindrical liquid jet, 515 nm, 3 kHz, 20 mJ/cm^2 pump.

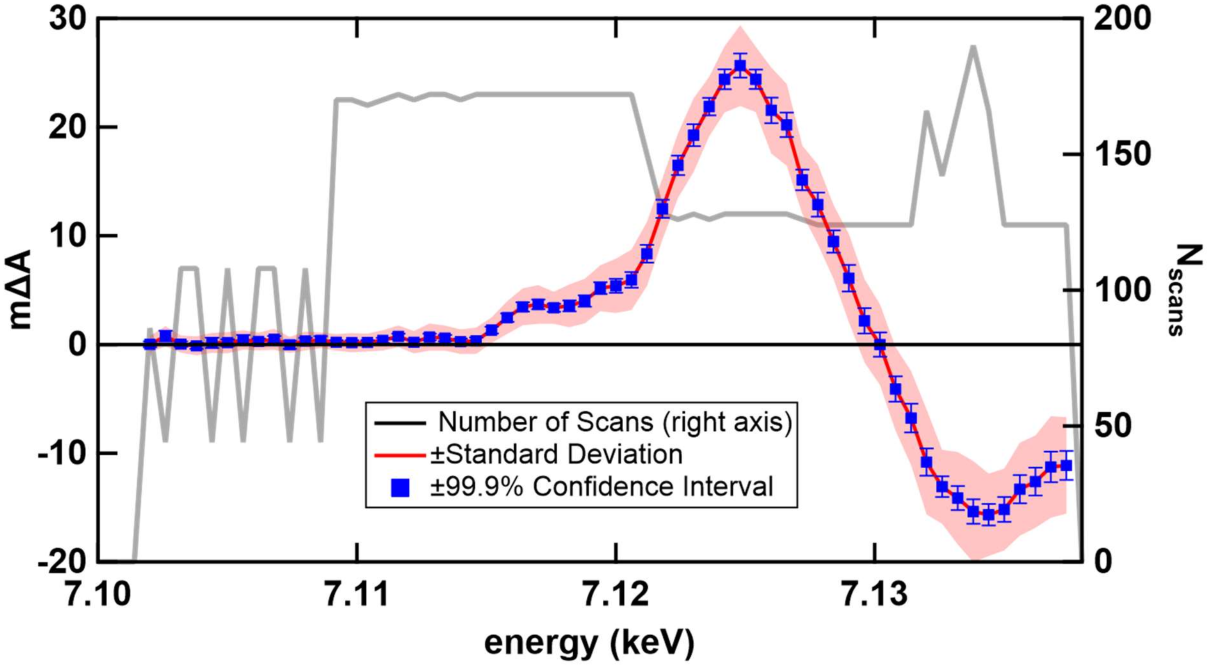

Figure S45: Comparison of the standard deviation and 99.9% confidence interval of the Fe K-edge transient XANES data at 150 ps. 5 mM [FcCc]PF_6 in MeCN, 700 µm cylindrical liquid jet, 515 nm, 3 kHz, 20 mJ/cm2 pump.

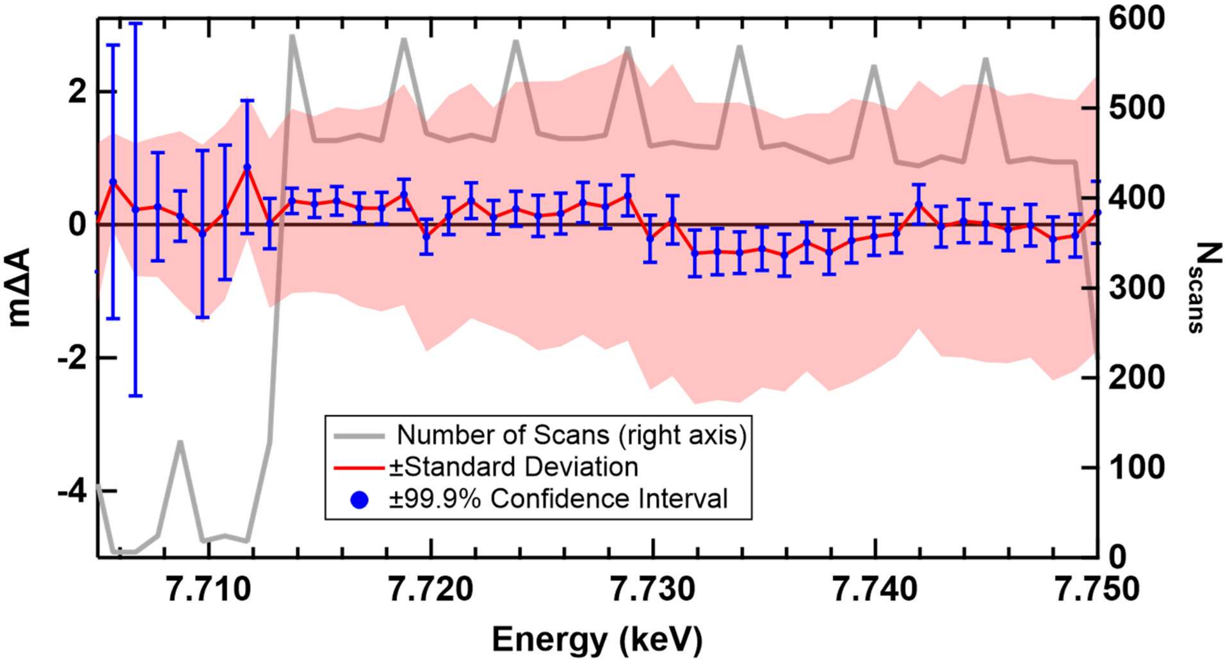

4.2 Kinetic Fitting

A kinetic trace of the XTA data was collected at the maximum of the ESA feature at the Fe K-edge (7.1248 keV). The data were fit with a single exponential convoluted with a gaussian instrument response function (IRF). The amplitude, time constant, FWHM of the gaussian IRF, and time zero (t_0) were varied as fit parameters. In Figure 6c of the Main Text, the kinetic trace has been shifted horizontally by the t_0 obtained in the fit.

Figure S46: Kinetic trace of Fe K-edge XTA signal at 7.124 keV. Error bars are the standard deviation of the data. The data were fit to a single exponential convoluted with a gaussian instrument response function (IRF). 5 mM [FcCc]PF_6 in MeCN, 700 µm cylindrical liquid jet, 515 nm, 3 kHz, 20 mJ/cm2 pump.

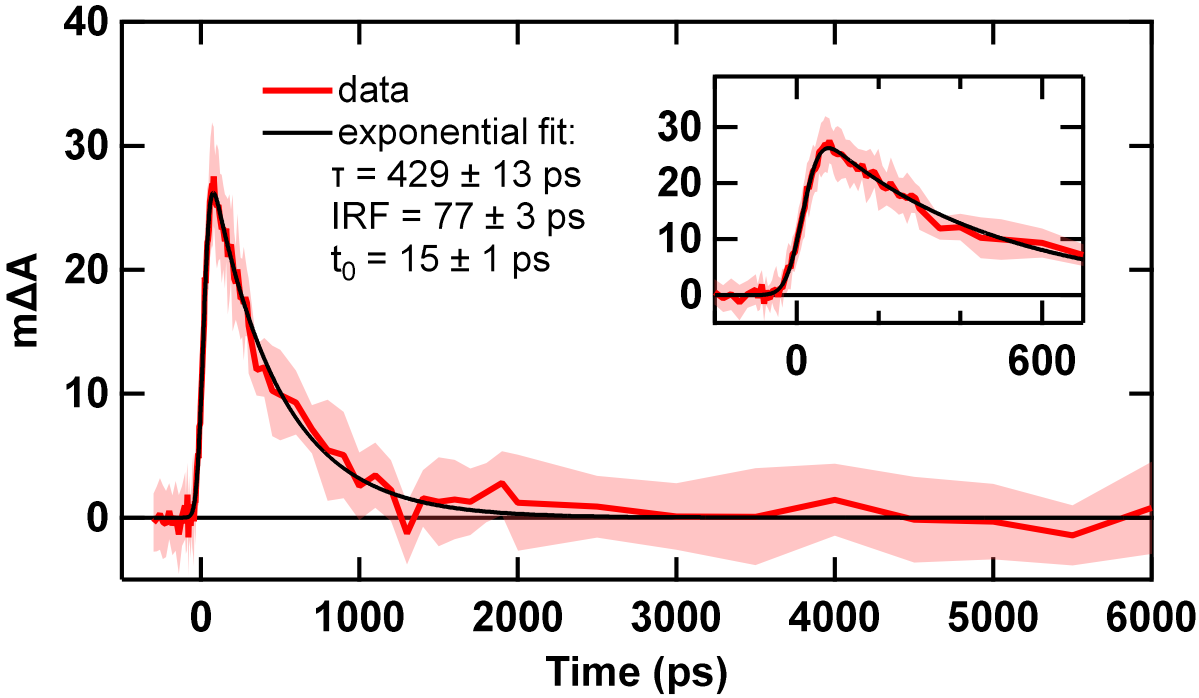

4.3 Pre-Edge Region

The pre-edge region is a sensitive probe of electronic structure and 3d orbital occupation. The low-spin, d^6 ground states of ferrocene and cobaltocenium lead to a single pre-edge transition to the vacant e_{1g} orbitals in the static Fe and Co K-edge spectra of [FcCc]PF_6. In the Fe K-edge XTA (Figure S47), we observe a pre-edge ESA with peaks at 7.1116 eV and 7.1128 eV. The double-peaked structure is likely due to competition of the excited-state pre-edge peak(s) with a bleach of the ground-state pre-edge peak. To gain further insight into the pre-edge signal, we performed TD-DFT calculations of the S_0, T_1, and Q_1 states of $[FcCc]^+$ in ORCA while restricting the initial orbital of the transitions to the Fe 1s orbital. The T_1 and Q_1 states coincidentally give very similar pre-edge spectra, despite the latter having one more 3d hole. The pre-edge ESA observed in experiment is consistent with a ligand-field excited state with more 3d holes than the low-spin Fe(II) ground state, but the similar $T_1 – S_0$ and $Q_1 – S_0$ theoretical difference spectra prevent us from using this pre-edge signal to discriminate between the ^{3}(d-d) and ^{5}(d-d) states.

Figure S47: Fe 1s pre-edge XTA spectrum and TD-DFT calculations. TD-DFT sticks and broadened spectra have been shifted by +22.8 eV to align with experiment. The sticks (plotted on the right axis) have been scaled by 0.3 for clarity. Error bars are ± 99.9% confidence interval.

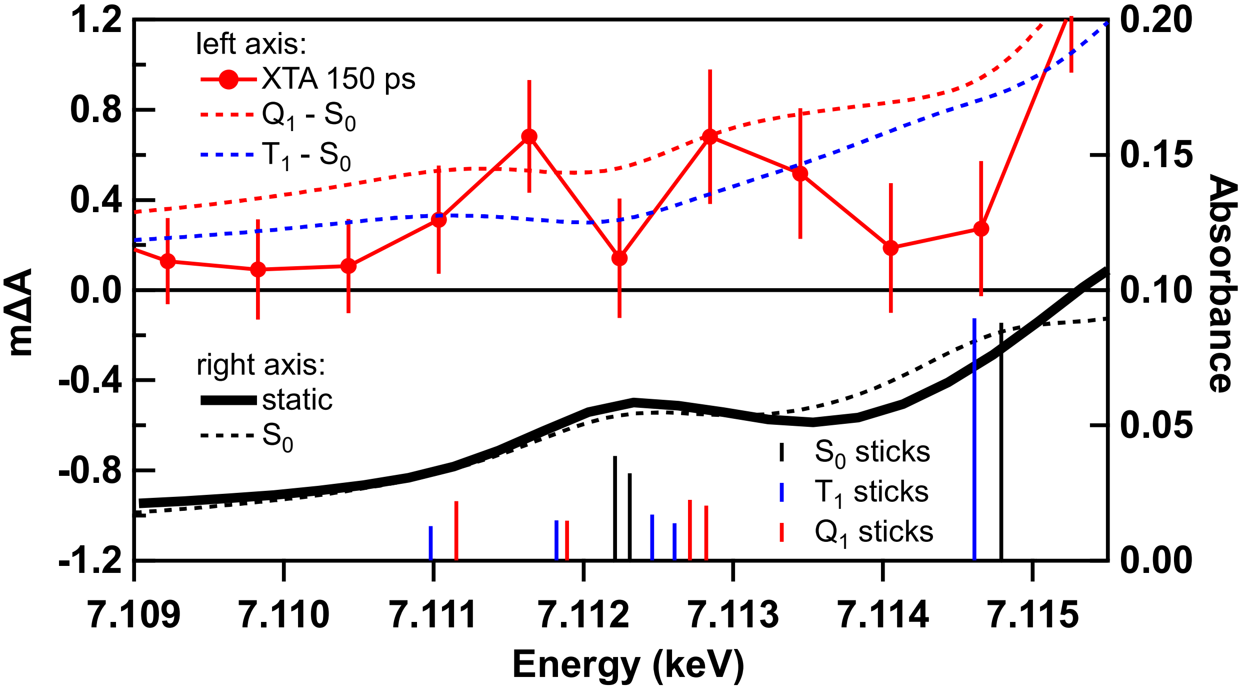

4.4 EXAFS Analysis

The EXAFS signal, χ(E), was isolated from the static Fe K-edge X-ray absorption spectrum through background subtraction in the program Athena with an Rbkg parameter of 1.0 and an edge position (or ionization threshold) of E_0 = 7.12 keV. The EXAFS spectrum in *k*-space, χ(k), where $k = \sqrt{2m_e(E - E_0)}/\hbar$ is the photoelectron wavevector, ħ is reduced Planck's constant, m_e is the mass of the electron, and E is the photon energy, is plotted below for various *k*-weights. *k*-weighting, where a *k*-weight of n corresponds to $\chi(k)^*k^n$, is used to enhance EXAFS features at high *k*. In Figure 8c of the Main Text, the EXAFS spectrum is shown in energy space with a *k*-weight of 2, i.e. $\chi(E)^*k^2$. *k*-space is a reciprocal space with units of $Å^{-1}$, and Fourier transform of the waveform gives the EXAFS signal in position space (R-space), χ(R). The Fourier transform was performed over a *k* range of 3 $Å^{-1}$ to 10.5 $Å^{-1}$ with a Hanning window of $\Delta k = 1\ Å^{-1}$.

EXAFS oscillations arise from scattering of the excited photoelectron off neighboring atoms. Wave interference of the emitted and backscattered photoelectron modulate the amplitude of the photoelectron wavefunction at the absorbing atom, thus modulating its overlap with the 1s core-hole wavefunction and the resulting absorption intensity. The modulated absorption intensity appears as interference fringes in *k*-space, which can be modeled with the phenomenological EXAFS equation:

$$\chi(k) = S_0^2 \sum_i^{paths} N_i \frac{f_i(k)}{kR_i^2} e^{-\frac{2R_i}{\lambda(k)}} e^{-2k^2\sigma_i^2} \sin\left(2kR_i + \delta_i(k)\right)$$

The sum is over all the scattering paths that contribute to the wave interference. S_0^2 is the amplitude reduction factor (which is usually between 0.7 and 1). is the degeneracy of the path, and R_i is half of the path length. For single scattering paths, N_i is the coordination number and R_i is the bond length. The scattering probability for a given path is $f_i(k)$ and the phase shift that the photoelectron experiences upon scattering is $\delta_i(k)$, both of which are *k*-dependent. The photoelectron mean free path, $\lambda(k)$, causes damping for paths with large R_i, while the thermal disorder parameter, σ_i^2, also known as the Debye-Waller factor, causes damping at high *k*.

Fitting of the data to the EXAFS equation was performed in the program Artemis. The structural model used was based on the DFT geometry-optimized structure of the S_0 state of $[FcCc]^+$. The scattering paths for this structure, including their degeneracies, effective path lengths, scattering probabilities, and phase shifts, were calculated by Feff with an R_{max} of 6 Å, degeneracy tolerances ("distance fuzz" and "angle fuzz") of 0.03 Å and 3°, and maximum n_{leg} of 4. The resulting structural model is highly symmetric, with all 10 Fe-C single scattering pathways being degenerate. We found that a minimal model containing only the 10-fold degenerate Fe-C single scattering pathway did not provide satisfactory fits. Previous studies found that including multiple-scattering pathways involving the C atoms of the cyclopentadiene rings is necessary to model the EXAFS of ferrocene at high *R*.[18,19] We therefore included all scattering pathways involving C atoms up to 4.1006 Å, while disregarding those that involve H atoms. This amounted to 18 pathways including Fe-C-Fe, Fe-C-C-Fe, Fe-C-C-C-Fe, and Fe-C-Fe-C-Fe, which are tabulated in Table 6. To specify the paths, we borrow the notation of Ruiz-Lopez *et al.*[18] in numbering the Fe atom as 0 and the C atoms as 1-10 (Figure S48), while adding

the additional indices 1’-5’ for the C atoms of the neighboring cobaltocenium cyclopentadiene ring.

The parameters that are fit in Artemis include the change in ionization threshold (ΔE_0) from the value originally selected in Athena (which was 7.12 keV), the change in half pathlength (ΔR) from the effective half pathlength (R_{eff}) found by Feff, the Debye-Waller factor (σ^2), which was assumed to be the same for each path, and the amplitude reduction factor, S_0^2. Each of these parameters may be expressed as a math expression that is dependent on other fit parameters. To avoid overfitting the data, we used the math expression $\Delta R = \alpha * R_{eff}$ for each scattering pathway, which allows us to use only one structural fitting parameter, α, which is the fractional amount by which each scattering path length is varied. In other words, the half path length obtained by the fit is $R = R_{eff} + \Delta R = R_{eff}*(1 + \alpha)$. The data were fit in R space with *k* weights of 1, 2, and 3.

The fitting parameters are tabulated in Table S5. From the fitted value of α, and the Fe-C single scattering path R_{eff} of 2.0503 Å, we obtain an Fe-C bond length of 2.039 ± 0.007 Å.

Table S5: Ground-state EXAFS fit parameters.

Parameter	Name in Artemis	Fitted Value ± Standard Deviation [Initial Value]	Units
α	alpha	-0.00548726 ± 0.00323749 [-0.005]	-
ΔE_0	enot	0.70454746 ± 1.19706271 [1.15235]	eV
S_0^2	amp	0.76936053 ± 0.06241626 [0.75]	-
σ^2	ss	0.00200409 ± 0.00084766 [0.002]	$Å^2$

Figure S48: Atom labels for EXAFS scattering paths.

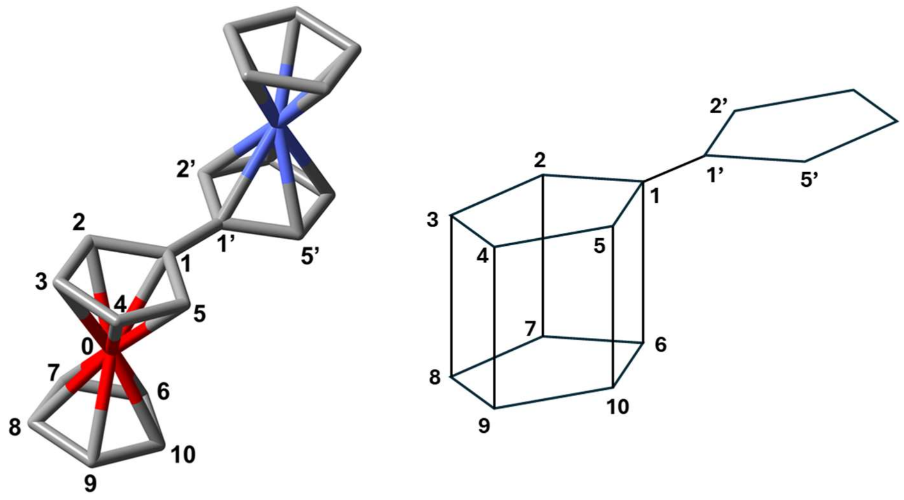

Table 6: Scattering pathways included in EXAFS model.

Index	Path	R_{eff} (Å)	n_{leg}	N
1	0-1-0	2.05	2	10
2	0-1-2-0	2.746	3	20
3	-	-	-	-
4	-	-	-	-
5	0-1'-0	3.116	2	1
6	-	-	-	-
7	0-1-3-0	3.204	3	20
8	0-1-1'-0	3.307	3	2
9	0-1-2-1-0	3.477	4	20
10	0-1-1'-1-0	3.499	4	1
11	-	-	-	-
12	0-1-6-0	3.702	3	10
13	0-1-7-0	3.85	3	20
14	0-2-1'-0	3.896	3	4
15	-	-	-	-
16	0-2'-0	4.041	2	2
17	0-1-8-0	4.066	3	20
18	0-1-0-1-0	4.101	4	10
19	0-1-0-3-0	4.101	4	20
20	0-1-0-2-0	4.101	4	20
21	0-1-0-8-0	4.101	4	20
22	0-1-0-7-0	4.101	4	20
23	0-1-0-6-0	4.101	4	10

Figure S49: Static Fe K-edge EXAFS spectrum in energy space and background spectrum. 5 mM $[FcCc]PF_6$ in MeCN, 700 μm cylindrical liquid jet.

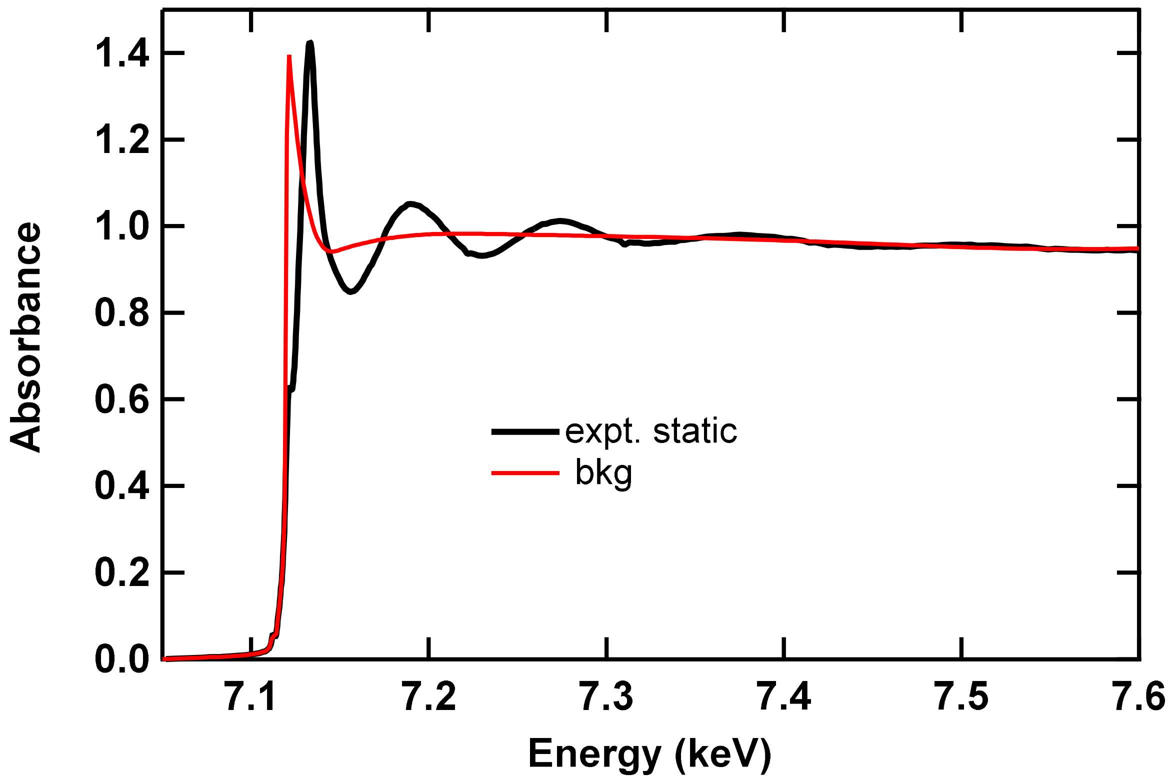

Figure S50: Static Fe K-edge EXAFS spectrum in k-space following background subtraction. Spectra with various k-weights are shown. 5 mM [FcCc]PF_6 in MeCN, 700 μm cylindrical liquid jet.

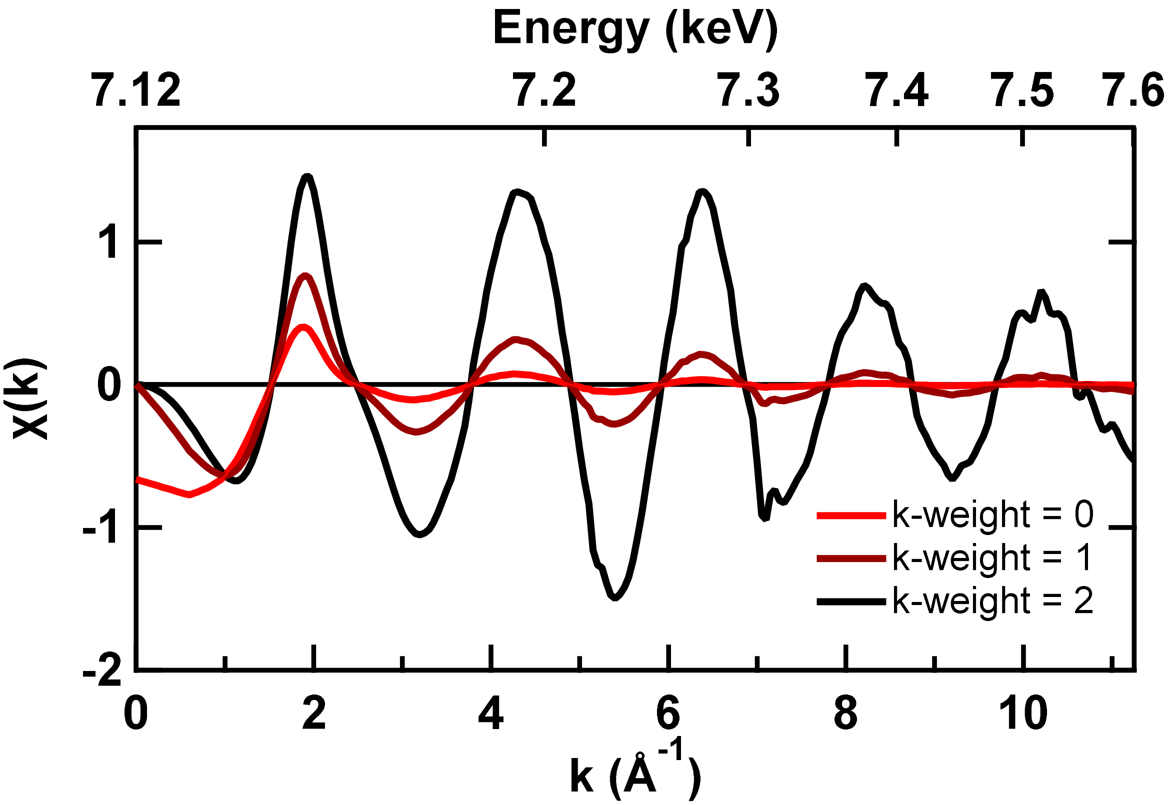

4.4.1 Artemis Log file of static (ground state) EXAFS fit.

```
ame             : Fit 260    (oypfw)
 Description     : fit to GS_and_8percXS_spectra.txt
 Figure of merit : 260
 Time of fit     : 2024-06-07T19:15:34
 Environment     : Demeter 0.9.26 with perl 5.024000 and using Ifeffit 1.2.12 on
Windows 10
 Interface       : Artemis (Wx 0.9928)
 Prepared by     :
 Contact         :


=*=*=*=*=*=*=*=*=*=*=*=*=*=*=*=*=*=*=*=*=*=*=*=*=*=*=*=*=*=*=*=*=*=*=*=*=*=*=


Independent points          : 14.1142578
Number of variables         : 4
Chi-square                  : 4251.6824147
Reduced chi-square          : 420.3652402
R-factor                    : 0.0135364
Number of data sets         : 1



guess parameters:
  alpha              =  -0.00548726    # +/-   0.00323749     [-0.005]
  enot               =   0.70454746    # +/-   1.19706271     [1.15235]
  amp                =   0.76936053    # +/-   0.06241626     [0.75]
  ss                 =   0.00200409    # +/-   0.00084766     [0.002]

set parameters:
  alpha2             =  -0.00500000
  ss2                =   0.00200000

Correlations between variables:
                enot & alpha             -->  0.8933
                  ss & amp               -->  0.8810
All other correlations below 0.4

===== Data set >> GS_and_8percXS_spectra.txt << ====================================

: name                    = GS_and_8percXS_spectra.txt
: k-range                 = 3 - 10.5
: dk                      = 1
: k-window                = Hanning
: k-weight                = 1,2,3
: R-range                 = 1 - 4
: dR                      = 0.2
: R-window                = Hanning
: fitting space           = r
: background function     = no
: phase correction        = no
: background removal      = E0: 7120, Rbkg: 1.0, range: [0:11.312], clamps: 0/24, kw:
2
: user-supplied epsilon_k = 0
: epsilon_k by k-weight   = 4.029e-004
: epsilon_r by k-weight   = 6.487e-002
: R-factor by k-weight    = 1 -> 0.01212,  2 -> 0.01294,  3 -> 0.01555

  name                      N       S02     sigma^2   e0     delr     Reff     R
=====================================================================================
==
[feff_artemis] C         10.000   0.769   0.00200   0.705 -0.01125  2.05030  2.03905
```

```
[feff_artemis] C C       20.000   0.769   0.00200   0.705 -0.01516  2.76350  2.74834
[feff_artemis] C          1.000   0.769   0.00200   0.705 -0.01710  3.11620  3.09910
[feff_artemis] C C       20.000   0.769   0.00200   0.705 -0.01758  3.20430  3.18672
[feff_artemis] C C        2.000   0.769   0.00200   0.705 -0.01815  3.30740  3.28925
[feff_artemis] C C C     20.000   0.769   0.00200   0.705 -0.01908  3.47670  3.45762
[feff_artemis] C C C      1.000   0.769   0.00200   0.705 -0.01920  3.49860  3.47940
[feff_artemis] C C       10.000   0.769   0.00200   0.705 -0.02032  3.70290  3.68258
[feff_artemis] C C       20.000   0.769   0.00200   0.705 -0.02113  3.85020  3.82907
[feff_artemis] C C        4.000   0.769   0.00200   0.705 -0.02123  3.86900  3.84777
[feff_artemis] C          2.000   0.769   0.00200   0.705 -0.02217  4.04090  4.01873
[feff_artemis] C C       20.000   0.769   0.00200   0.705 -0.02231  4.06590  4.04359
[feff_artemis] C         10.000   0.769   0.00200   0.705 -0.02250  4.10060  4.07810
[feff_artemis] C         20.000   0.769   0.00200   0.705 -0.02250  4.10060  4.07810
[feff_artemis] C         20.000   0.769   0.00200   0.705 -0.02250  4.10060  4.07810
[feff_artemis] C         20.000   0.769   0.00200   0.705 -0.02250  4.10060  4.07810
[feff_artemis] C         20.000   0.769   0.00200   0.705 -0.02250  4.10060  4.07810
[feff_artemis] C         10.000   0.769   0.00200   0.705 -0.02250  4.10060  4.07810

  name                       ei       third     fourth
========================================================
[feff_artemis] C         0.00000   0.00000   0.00000
[feff_artemis] C C       0.00000   0.00000   0.00000
[feff_artemis] C         0.00000   0.00000   0.00000
[feff_artemis] C C       0.00000   0.00000   0.00000
[feff_artemis] C C       0.00000   0.00000   0.00000
[feff_artemis] C C C     0.00000   0.00000   0.00000
[feff_artemis] C C C     0.00000   0.00000   0.00000
[feff_artemis] C C       0.00000   0.00000   0.00000
[feff_artemis] C C       0.00000   0.00000   0.00000
[feff_artemis] C C       0.00000   0.00000   0.00000
[feff_artemis] C         0.00000   0.00000   0.00000
[feff_artemis] C C       0.00000   0.00000   0.00000
[feff_artemis] C         0.00000   0.00000   0.00000
[feff_artemis] C         0.00000   0.00000   0.00000
[feff_artemis] C         0.00000   0.00000   0.00000
[feff_artemis] C         0.00000   0.00000   0.00000
[feff_artemis] C         0.00000   0.00000   0.00000
[feff_artemis] C         0.00000   0.00000   0.00000


=*=*=*=*=*=*=*=*=*=*=*=*=*=*=*=*=*=*=*=*=*=*=*=*=*=*=*=*=*=*=*=*=*=*=*=*=*=*=*=
```

Figure S51: Real part of the background-subtracted static EXAFS in *k* space and corresponding fits (black traces). Traces are vertically offset by 2 and scaled by the specified factors for clarity.

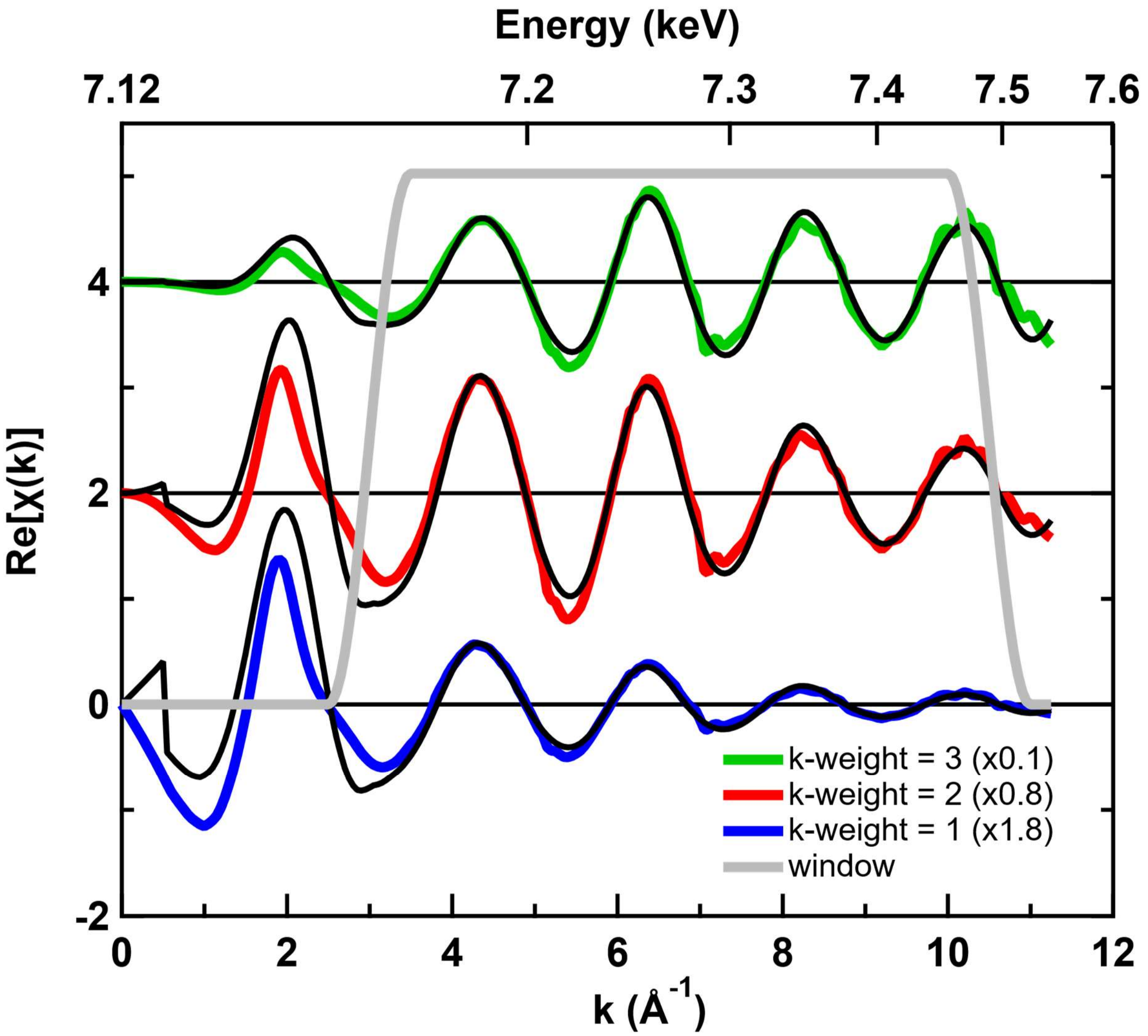

Figure S52: Magnitude of Fourier transform of static EXAFS in position space and corresponding fits (black traces).

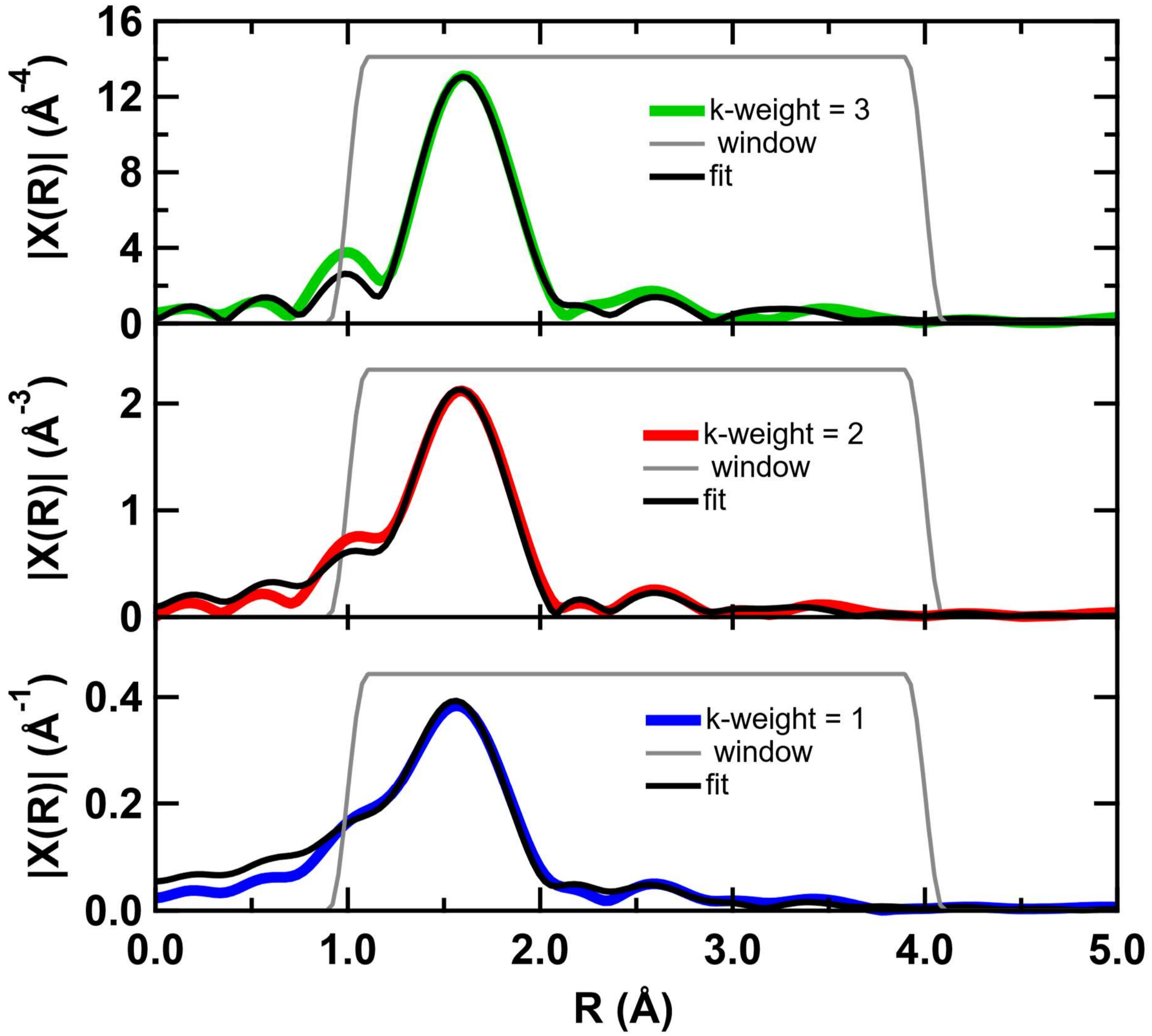

Figure S53: Real part of Fourier transform of static EXAFS in position space and corresponding fits (black traces).

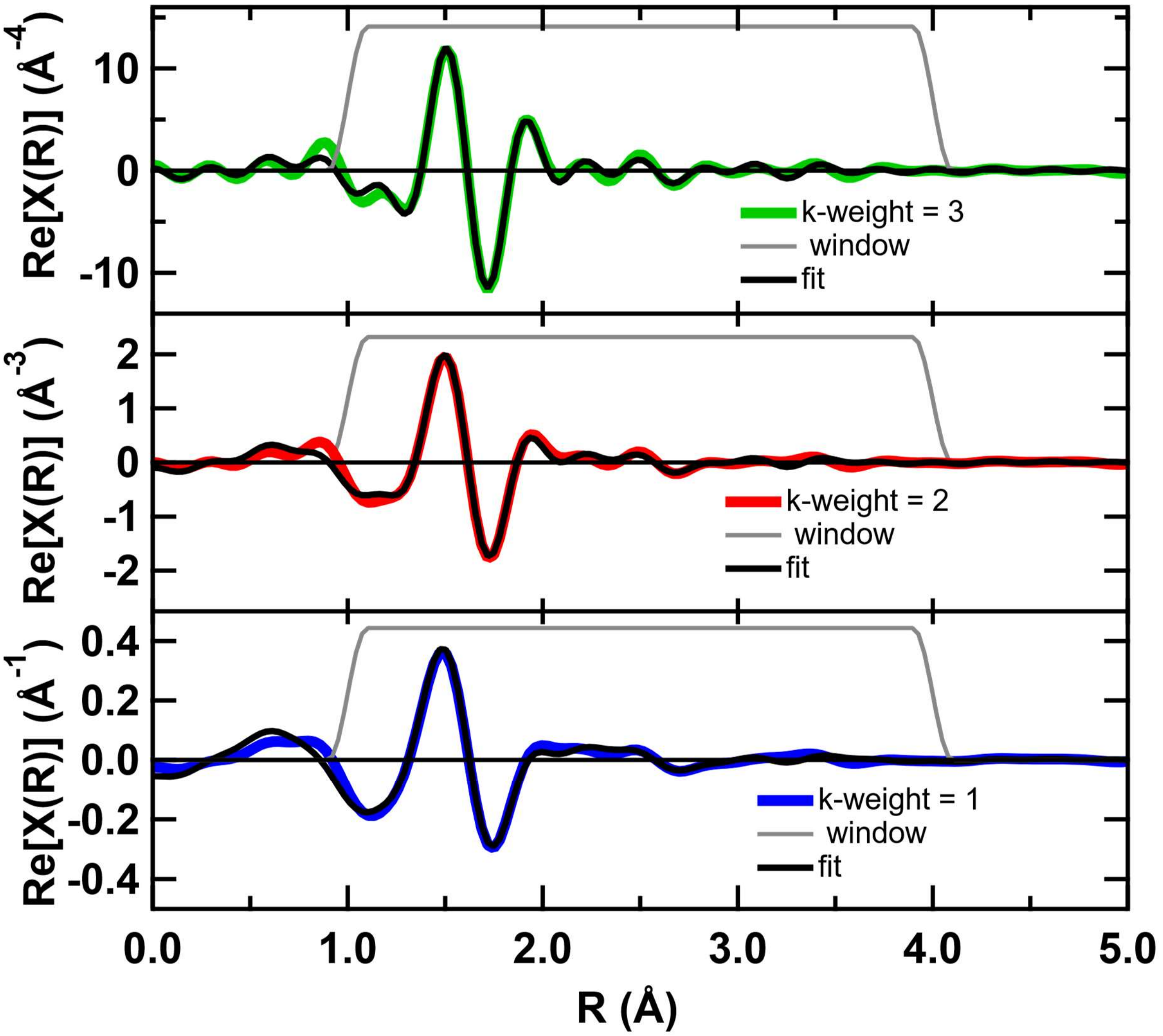

4.5 Excited-State EXAFS Fitting

Fitting the excited-state EXAFS presents additional challenges. First, reconstructing the excited-state spectrum from the transient spectrum requires knowledge of the excitation fraction, which can be difficult to accurately estimate. Second, the low signal-to-noise ratio of the XTA spectrum compared to the static spectrum severely limits the k range of the data. Third, the different ionization threshold of the excited state compared to the ground state changes the calculation of k and k-weighting between the ground and excited state.

For this reason, we followed the procedure outlined by Gawelda *et al.*[20] which overcomes these challenges by fitting the XTA spectrum to a model excited-state minus ground-state difference EXAFS spectrum *in energy space*, $\chi_{XS}(E) - \chi_{GS}(E)$. The ground-state model EXAFS spectrum is fixed to that obtained from the ground-state fit. Then, excited-state model EXAFS spectra are calculated for different ionization thresholds and changes in structure. For a structural model of the excited state, we considered changes in geometry where the Fe-C distances are symmetrically expanded while all C-C bond lengths are held constant. We denote the change in ionization threshold in the excited state (relative to the fitted the ground-state value) as $\Delta E_0^{(XS)}$, the ground-state Fe-C bond length as R, and the change in Fe-C bond length as ΔR. Besides varying ΔR and $\Delta E_0^{(XS)}$, we kept all other parameters, including the amplitude reduction factor and Debye-Waller factor the same. In Table S7, we express how the path length of each scattering path changes with ΔR. For the multiple-scattering pathways involving the C atoms of different rings, changes in ΔR result in changes in the interring C-C distances. In Figure S54 through Figure S58, we derive the changes in these C-C distances as a function of ΔR. For the structural parameters included in these derivations, such as the distance between C atoms 1 and 2 (d_{12}), and the C-Fe-C angle between atoms 1, 0, and 6 (θ_{106}), we use the average values from the DFT geometry-optimized structure of the S_0 state of $[FcCc]^+$.

The fit starts from the fitted structure of the ground state. In other words, the fitted change in each scattering path is $R_{eff} * \alpha + (excited\ state\ changes)$, where α is fixed to the value obtained in the ground-state fit described in SI Section 4.4.

For each set of parameters $\Delta E_0^{(XS)}$ and ΔR, we calculate the reduced chi-square, χ^2, for various excitation fractions, f, through the formula

$$\chi^2 = \frac{1}{N_{dataPts} - DOF} \sum_{i}^{N_{dataPts}} \left(\frac{\frac{\Delta A_{data}(E_i)}{f} - \Delta A_{fit}(E_i)}{\frac{\sigma_{data}}{f}} \right)^2$$

Equation S5

where $N_{dataPts}$ is the number of data points that are included in the fitting range, $\Delta A_{data}(E_i)$ is the XTA signal at energy E_i, $\Delta A_{fit}(E_i)$ is the model $\chi_{XS}(E) - \chi_{GS}(E)$ spectrum at energy E_i, σ_{data} is the error in the data, taken here to be the 99.9% confidence interval defined in Section 4.1, and DOF

is the degrees of freedom, taken here to be 3 for the fitting parameters of $\Delta E_0^{(XS)}$, ΔR, and *f*. The fitting range used was 7.1536 keV to 7.4055 keV, which amounted to 50 data points.

By calculating χ^2 for a range of of parameter values, we construct surfaces that allow us to find a minimum representing the best fit. These surfaces are shown as slices along Δ*R* in Figure S59. We found that the surfaces for each $\Delta E_0^{(XS)}$ give minima at *f* = 3% and Δ*R* = 0.25 Å. As in Figure S60, the χ^2 surfaces show only a weak $\Delta E_0^{(XS)}$ dependence, with the minimum being at $\Delta E_0^{(XS)}$ = -0.8 eV. For comparison, Gawelda *et al.*[20] found ΔE_0 = -1.25 eV for the high-spin excited state of $[Fe(bpy)_3]^{2+}$. The error (as 1σ) in the fitted value of Δ*R* can be estimated as the displacement along the Δ*R* that increases χ^2 by 1.[21] With this procedure, we find a fitted value of Δ*R* = 0.25± 0.1 Å corresponding to an Fe-C bond length of 2.29 ± 0.1 Å in the excited state. Figure S61 shows a comparison of the fitted change in bond length with those obtained by DFT for the T_1 and Q_1 fit. The bond-length increase for the Q_1 state of Δ*R* = 0.29 falls within the error of the fitted Δ*R* value, whereas the smaller bond-length increase of Δ*R* = 0.10 in the T_1 state is below this range.

Table S7: Scattering pathways included in EXAFS model and expressions for the change in the path length as a function of Fe-C bond-length increase (ΔR) used for excited-state model.

Index	Path	R_{eff} (Å)	n_{leg}	N	Half Path Length	Change in Half Path Length
1	0-1-0	2.05	2	10	0.5*(2*R)	0.5*(2*ΔR)
2	0-1-2-0	2.746	3	20	0.5*(2*R + d_{12})	0.5*(2*ΔR)
3	-	-	-	-	-	-
4	-	-	-	-	-	-
5	0-1'-0	3.116	2	1	0.5*(2*$d_{01'}$)	0.5*(2*Δ$d_{01'}$)
6	-	-	-	-	-	-
7	0-1-3-0	3.204	3	20	0.5*(2*R + d_{13})	0.5*(2*ΔR)
8	0-1-1'-0	3.307	3	2	0.5*(R + $d_{11'}$ + $d_{01'}$)	0.5*(ΔR + Δ$d_{01'}$)
9	0-1-2-1-0	3.477	4	20	0.5*(2*R + 2*d_{12})	0.5*(2*ΔR)
10	0-1-1'-1-0	3.499	4	1	0.5*(2R + 2*$d_{11'}$)	0.5*(2ΔR)
11	-	-	-	-	-	-
12	0-1-6-0	3.702	3	10	0.5*(2*R + d_{16})	0.5*(2*ΔR + Δd_{16})
13	0-1-7-0	3.85	3	20	0.5*(2*R + d_{17})	0.5*(2*ΔR + Δd_{17})
14	0-2-1'-0	3.896	3	4	0.5*(2*R + $d_{21'}$)	0.5*(2*ΔR)
15	-	-	-	-	-	-
16	0-2'-0	4.041	2	2	0.5*(2*$d_{02'}$)	0.5*(2*Δ$d_{02'}$)
17	0-1-8-0	4.066	3	20	0.5*(2*R + d_{18})	0.5*(2*ΔR + Δd_{18})
18	0-1-0-1-0	4.101	4	10	0.5*(4*R)	0.5*(4*ΔR)
19	0-1-0-3-0	4.101	4	20	0.5*(4*R)	0.5*(4*ΔR)
20	0-1-0-2-0	4.101	4	20	0.5*(4*R)	0.5*(4*ΔR)
21	0-1-0-8-0	4.101	4	20	0.5*(4*R)	0.5*(4*ΔR)
22	0-1-0-7-0	4.101	4	20	0.5*(4*R)	0.5*(4*ΔR)
23	0-1-0-6-0	4.101	4	10	0.5*(4*R)	0.5*(4*ΔR)

Figure S54: Change in distance between C atoms 1 and 6 as a function of Fe-C bond length (R) and Fe-C bond length increase (ΔR) for excited-state EXAFS model.

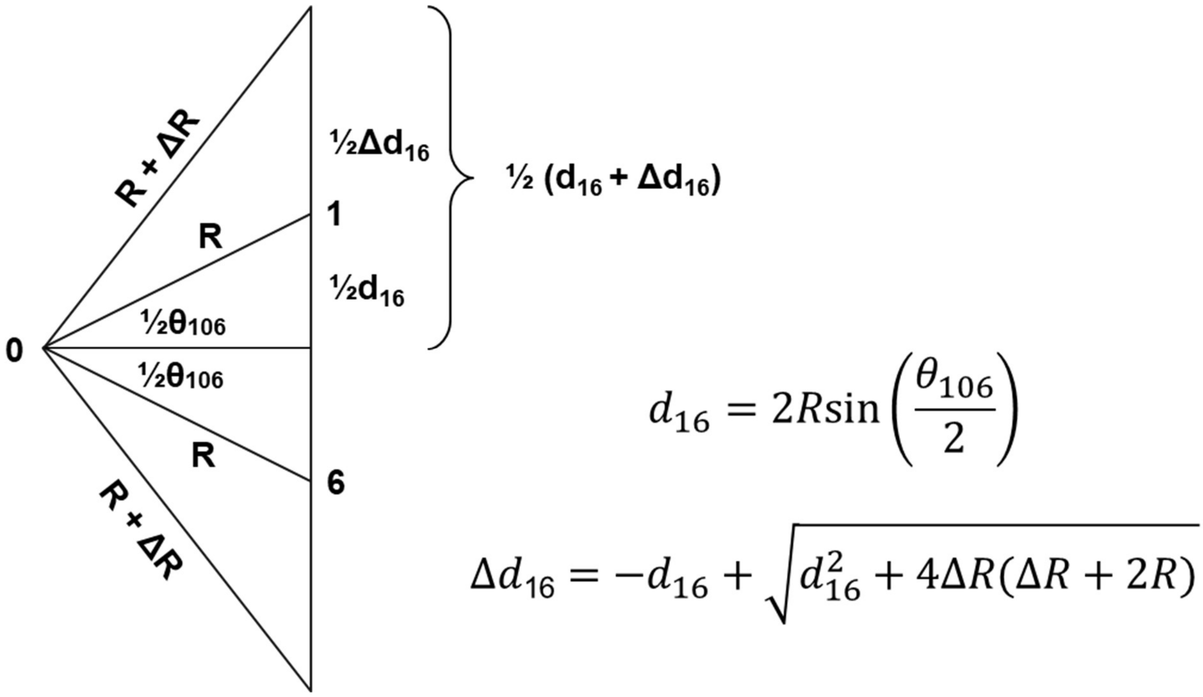

$$d_{16} = 2R\sin\left(\frac{\theta_{106}}{2}\right)$$

$$\Delta d_{16} = -d_{16} + \sqrt{d_{16}^{2} + 4\Delta R(\Delta R + 2R)}$$

Figure S55: Change in distance between C atoms 1 and 8 as a function of Fe-C bond length (R) and Fe-C bond length increase (ΔR) for excited-state EXAFS model. See Figure S54 for expression of d_{16}.

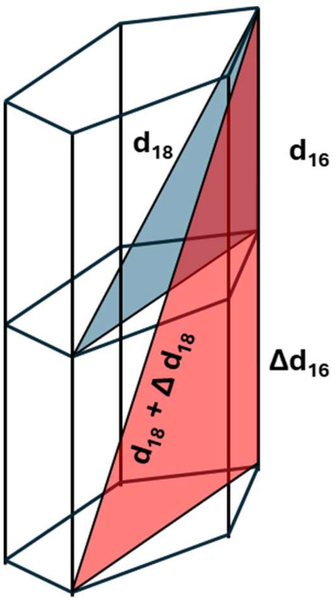

$$d_{18} = \sqrt{2d_{12}^2\left(1-\cos\left(\frac{3\pi}{5}\right)\right)+d_{16}^2}$$

$$d_{18} + \Delta d_{18} = \sqrt{2d_{12}^2\left(1-\cos\left(\frac{3\pi}{5}\right)\right)+(d_{16}+\Delta d_{16})^2}$$

$$\Delta d_{18} = \sqrt{2d_{12}^2\left(1-\cos\left(\frac{3\pi}{5}\right)\right)+(d_{16}+\Delta d_{16})^2} - d_{18}$$

Figure S56: Change in distance between C atoms 1 and 7 as a function of Fe-C bond length (R) and Fe-C bond length increase (ΔR) for excited-state EXAFS model. See Figure S54 for expression of d_{16}.

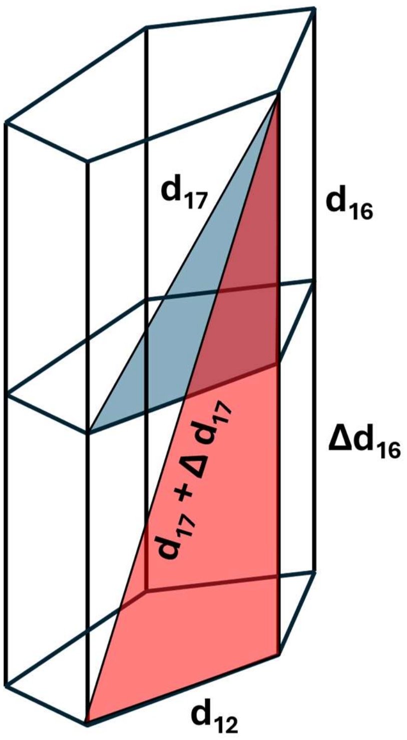

$$d_{17} = \sqrt{d_{12}^2 + d_{16}^2}$$

$$d_{17} + \Delta d_{17} = \sqrt{d_{12}^2 + (d_{16} + \Delta d_{16})^2}$$

$$\Delta d_{17} = \sqrt{d_{12}^2 + (d_{16} + \Delta d_{16})^2} - d_{17}$$

Figure S57: Change in distance between Fe atom and C atom 1' of the cobaltocenium ring, expressed as a function of Fe-C bond length (R) and Fe-C bond length increase (ΔR) for excited-state EXAFS model. See Figure S54 for expression of d_{16}.

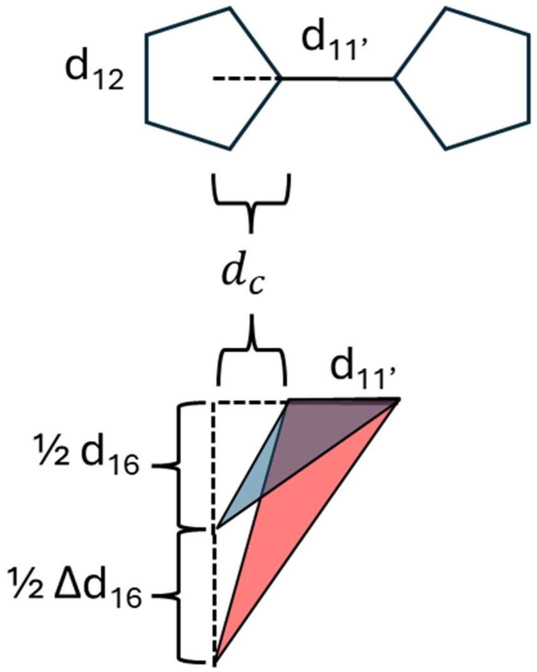

$$d_c = \frac{d_{12}\sqrt{50 + 10\sqrt{5}}}{10}$$

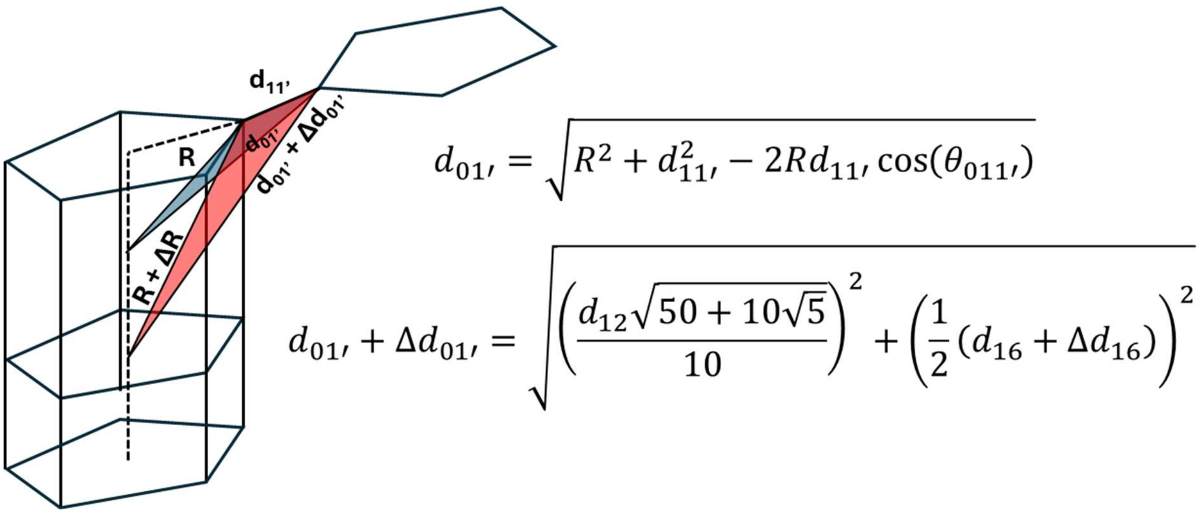

$$d_{01'} = \sqrt{R^2 + d_{11'}^2 - 2Rd_{11'}\cos(\theta_{011'})}$$

$$d_{01'} + \Delta d_{01'} = \sqrt{\left(\frac{d_{12}\sqrt{50 + 10\sqrt{5}}}{10}\right)^2 + \left(\frac{1}{2}(d_{16} + \Delta d_{16})\right)^2}$$

Figure S58: Change in distance between Fe atom and C atom 2' of the cobaltocenium ring, expressed as a function of Fe-C bond length (R) and Fe-C bond length increase (ΔR) for excited-state EXAFS model. See Figure S54 for expression of d_{16}.

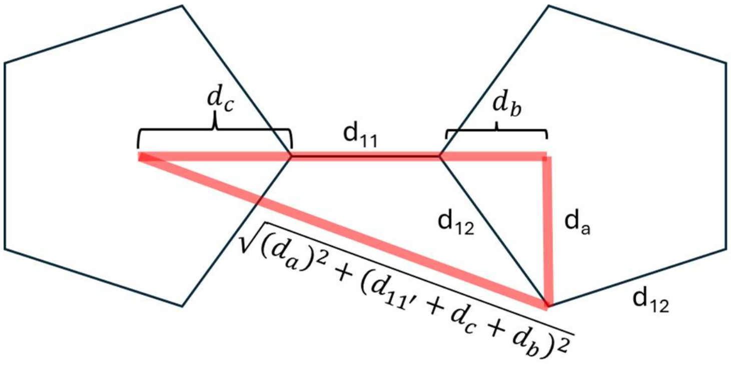

$$d_a = \frac{1}{4} d_{12}\left(1 + \mathrm{sqrt}(5)\right)$$

$$d_b = \mathrm{d}_{12}\sqrt{1 - \frac{1}{16}\left(1 + \mathrm{sqrt}(5)\right)^2}$$

$$d_c = \frac{d_{12}\sqrt{50 + 10\sqrt{5}}}{10}$$

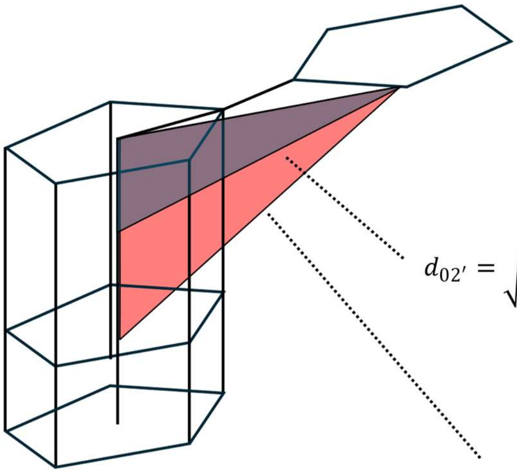

$$d_{02'} = \sqrt{\left((d_a)^2 + (d_{11'} + d_c + d_b)^2\right) + \left(\frac{1}{2} d_{16}\right)^2}$$

$$d_{02'} + \Delta d_{02'} =$$

$$\sqrt{\left(\frac{1}{4} d_{12}\left(1 + \mathrm{sqrt}(5)\right)\right)^2 + \left(d_{11'} + \frac{d_{12}\sqrt{50 + 10\sqrt{5}}}{10} + \mathrm{d}_{12}\sqrt{1 - \frac{1}{16}\left(1 + \mathrm{sqrt}(5)\right)^2}\right)^2 + \left(\frac{1}{2}(d_{16} + \Delta d_{16})\right)^2}$$

4.5.2 Parameters used in excited-state EXAFS fitting.

```
set parameters:
  enot              =  -0.89545000    # [-0.89545 ]
  amp               =   0.76936000
  ss                =   0.00200000
  delR              =   0.45000000
  alpha             =  -0.00549000
  R                 =   2.05000000
  d12               =   1.42642000
  d13               =   2.30798000
  theta106          =   1.87489265    # [107.423436*pi/180]
  d11p              =   1.45392000
  theta011p         =   2.18283373    # [125.06716*pi/180]

def parameters:
  d16               =   3.30480230    # [2*R*sin(theta106/2)]
  d17               =   3.59949889    # [sqrt(d12^2 + d16^2)]
  d18               =   4.03095076    # [sqrt(2*d12^2*(1 - cos(3*pi/5)) + d16^2)]
  d01p              =   3.13766590    # [sqrt( ( dc + d11p )^2  + (0.5*(d16))^2 )]
  da                =   1.15399802    # [0.25*d12*(1+sqrt(5))]
  db                =   0.83842864    # [sqrt(d12^2 - da^2)]
  dc                =   1.21338533    # [d12*sqrt(50 +10*sqrt(5))/10]
  d02p              =   4.04379916    # [sqrt( (da^2 + (d11p + dc + db)^2) +
(0.5*d16)^2)]
  del01p            =   0.31087688    # [sqrt( ( dc + d11p )^2  + (0.5*(d16+del16))^2
) - d01p]
  del02p            =   0.24570100    # [sqrt( (da^2 + (d11p + dc + db)^2) +
(0.5*(d16 + del16))^2) - d02p]
  del16             =   1.06689282    # [-d16 + sqrt(d16^2 + 4*delR*(delR + 2*R))]
  del17             =   0.99902177    # [sqrt( d12^2 + (d16 + del16)^2 ) - d17 ]
  del18             =   0.91258684    # [sqrt(2*d12^2*(1 - cos(3*pi/5)) + (d16 +
del16)^2) - d18]
```

Figure S59: Reduced chi-squared surfaces along change in bond length for various shifts in absorption edge.

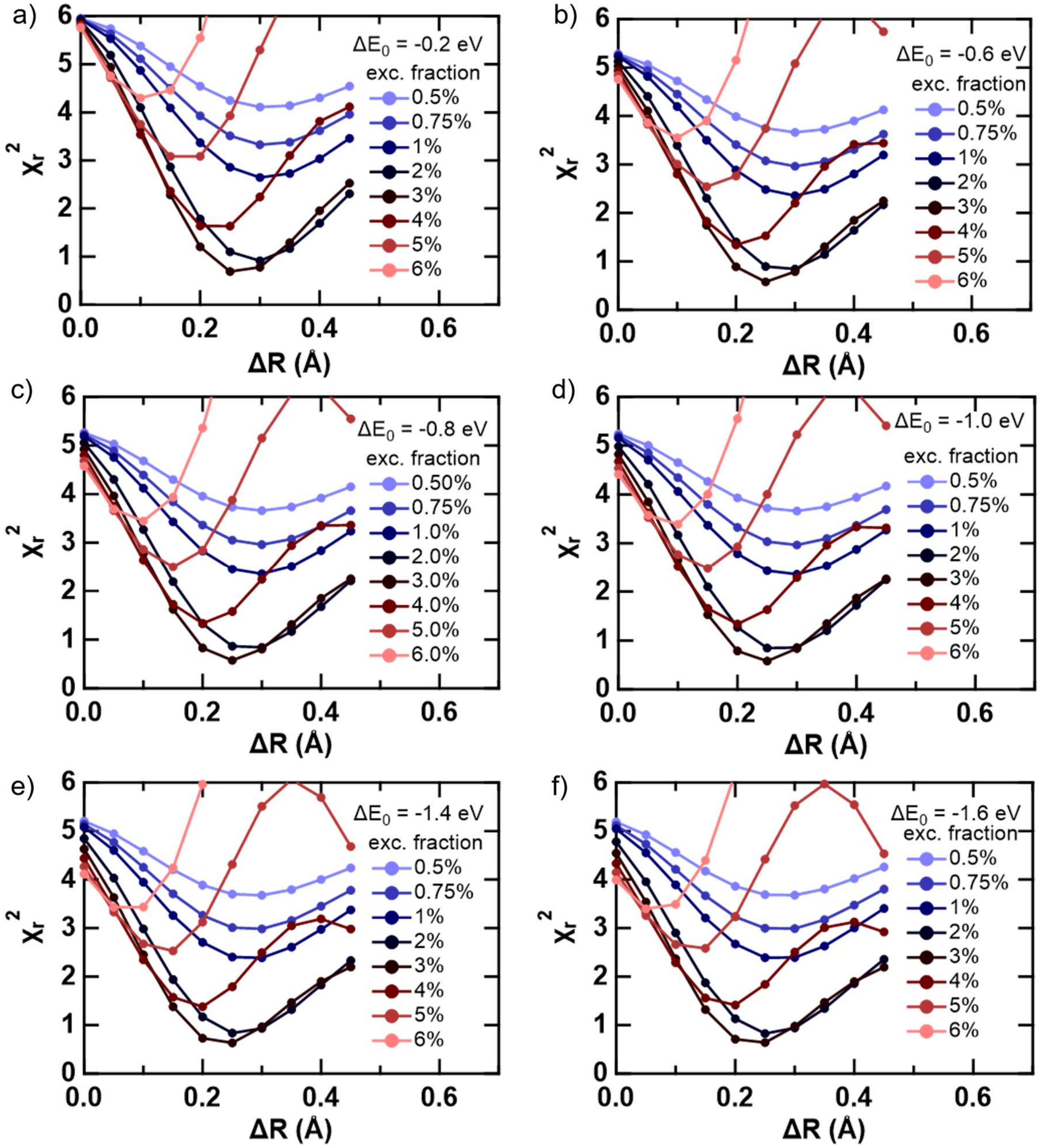

Figure S60: Reduced chi-squared surface along change in absorption edge for an excitation fraction of 3% and change in bond length of 0.25 Å.

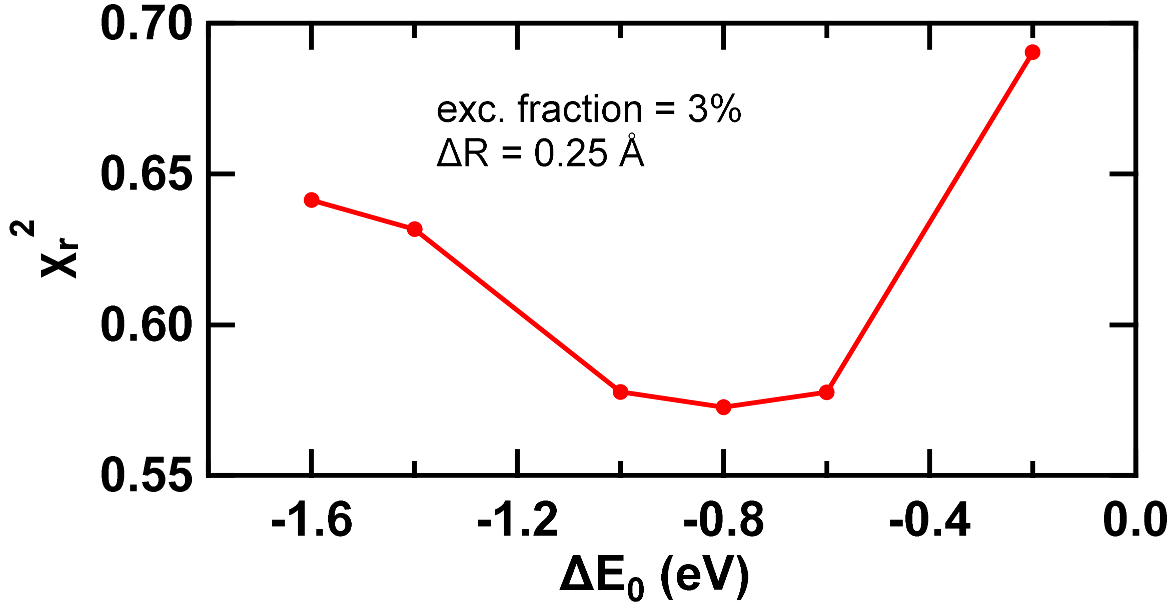

Figure S61: Reduced chi-squared surface along change in bond length compared to changes in bond length calculated by DFT. The DFT values, given by the vertical dashed lines, represent the change in average Fe-C bond length between the S_0 state and the T_1 (vertical blue dashed line) or Q_1 (vertical blue dashed line) states. The horizontal dashed line represents $\Delta\chi^2 = +1$ from the minimum and bounds the 68% confidence region (±1σ) of the fitted ΔR parameter.

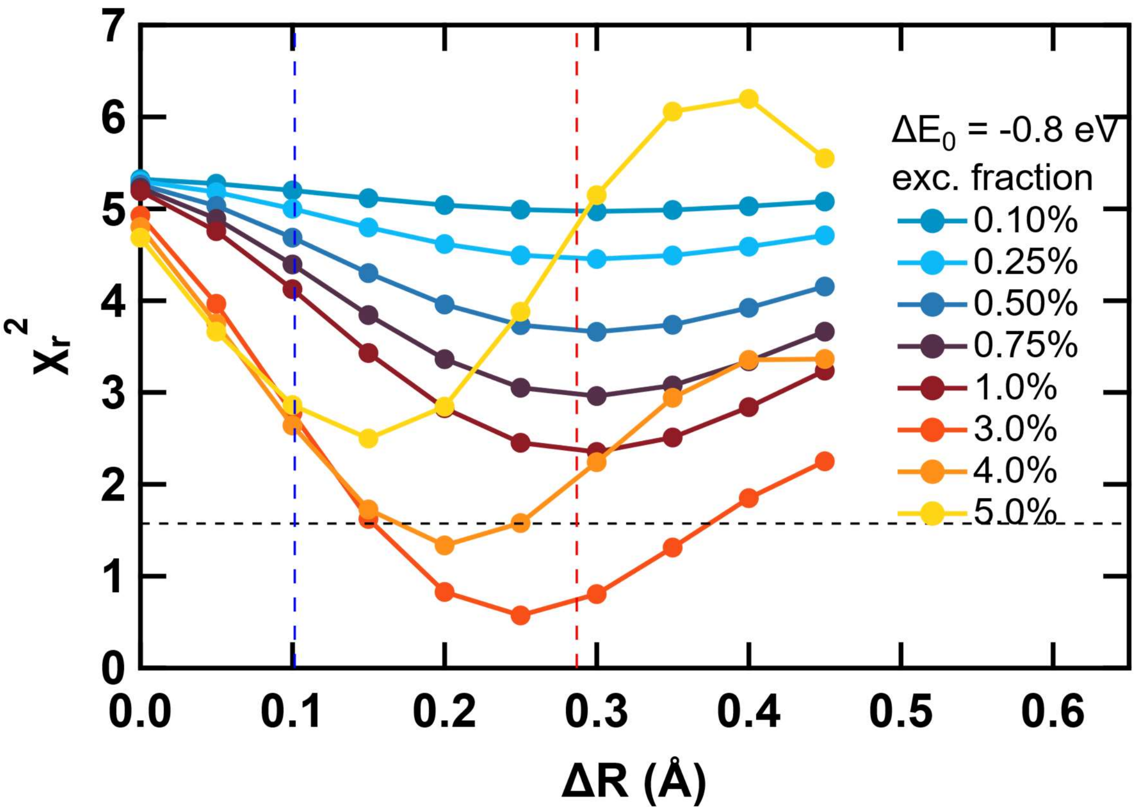

5 Real-Space Green's Function Theory

Real-space Green's function theory was used to calculate the K-edge absorption spectra. The calculations were carried out in the FEFF10 software package.[22,23]. These calculations employ a muffin-tin potential based on the atomic coordinates fed into the program. As input geometries, we used the DFT geometry-optimized coordinates of the S_0, T_1, or Q_1 state of $[FcCc]^+$ at the B3PW91/6-311+G(d) PCM (acetonitrile) level. Spin degrees of freedom are not included in the calculations. The overall positive charge of the molecule was accounted for with the ION card, initially distributing the +1 charge evenly across all constituent atoms. The core-hole was treated by the final state rule. A cluster radius of 9 Å, centered around the absorbing atom, was selected for all self-consistent field (SCF) and scattering path calculations to encompass every atom in the molecule. For calculation of XANES spectra, we employed the full multiple scattering method. The EXAFS spectra, on the other hand, were calculated by the path expansion method. Both XANES and EXAFS calculations included a maximum of 30 SCF cycles to achieve self-consistent potentials. Difference spectra were generated by subtracting the spectrum of the S0 geometry from the spectrum of the T_1 or Q_1 geometry, thus simulating the experimental pump-on minus pump-off (excited state minus ground state) transient spectra. For the EXAFS spectra, the ABSOLUTE card was used to avoid artifacts in the difference spectrum caused by normalization of the S0 and Q1 spectra prior to subtraction. To achieve absolute energy agreement with experiment, the spectra were shifted by -10 eV. The intensities of the calculated spectra were also scaled (by the same amount when on the same vertical scale) to match the experimental intensities.

5.1 Example FEFF10 input file for calculating XANES spectrum:

```
TITLE FcCc


ION 0 0.025

ION 1 0.025

ION 2 0.025

ION 3 0.025


SCF 9 1 30 0.2 1

S02 1.0


CONTROL 1 1 1 1 1 1


FMS 9 0

EDGE K

COREHOLE FSR

EGRID

e_grid  -10  100.0  0.5


LDOS -20 20 0.2

XANES 20.0 0.07 0.0


POTENTIALS

0 26 Fe

1 27 Co

2 6 C

3 1 H


ATOMS
```

0.23225	-2.55859	0.00194	0 Fe
-0.24010	2.54564	-0.00161	1 Co
-1.18568	-3.46540	1.17668	2 C
-0.22115	-4.41841	0.73620	2 C
-0.23338	-4.43190	-0.68898	2 C
-1.20539	-3.48715	-1.13054	2 C
-1.79395	-2.89161	0.02278	2 C
1.16656	-1.13823	1.13568	2 C
2.11373	-2.09893	0.69152	2 C
2.10057	-2.11246	-0.73332	2 C
1.14412	-1.16130	-1.17811	2 C
0.56185	-0.54081	-0.02166	2 C
-0.47788	0.47547	-0.02365	2 C
-1.06369	1.09811	-1.17443	2 C
1.14760	3.47272	1.17103	2 C
0.15922	4.40690	0.74386	2 C
0.16396	4.43128	-0.67944	2 C
1.15509	3.51189	-1.13171	2 C
1.76608	2.92308	0.01197	2 C
-1.09303	1.07590	1.12349	2 C
-2.07257	2.00844	0.68278	2 C
-2.05467	2.02217	-0.74093	2 C
0.40761	-5.02869	-1.32389	3 H
-1.43443	-3.24286	-2.15903	3 H
-2.54483	-2.11273	0.02136	3 H
-1.39677	-3.20363	2.20475	3 H
0.43077	-5.00294	1.37130	3 H
2.68772	-2.76192	-1.36842	3 H

2.71296	-2.73567	1.32764	3 H
0.88902	-0.95683	-2.20879	3 H
2.52217	2.15079	0.00172	3 H
1.35618	3.19411	2.19427	3 H
-0.51300	4.95807	1.38633	3 H
-0.50338	5.00387	-1.30765	3 H
1.36947	3.26834	-2.16268	3 H
-0.78157	0.91916	-2.20219	3 H
-2.68154	2.63509	1.31889	3 H
-2.64794	2.66045	-1.38020	3 H
-0.83892	0.87620	2.15461	3 H
0.93263	-0.91223	2.16693	3 H

END

5.2 Example FEFF10 input file for calculating EXAFS spectrum:

```
TITLE FcCc

ION 0 0.025
ION 1 0.025
ION 2 0.025
ION 3 0.025

CONTROL 1 1 1 1 1 1

COREHOLE FSR
RPATH 9.0
SCF 9 1 30 0.2 1
EXAFS 20.0

ABSOLUTE

POTENTIALS
0 26 Fe
1 27 Co
2 6 C
3 1 H

ATOMS
  0.23225   -2.55859    0.00194    0 Fe
 -0.24010    2.54564   -0.00161    1 Co
 -1.18568   -3.46540    1.17668    2 C
 -0.22115   -4.41841    0.73620    2 C
 -0.23338   -4.43190   -0.68898    2 C
```

-1.20539	-3.48715	-1.13054	2 C
-1.79395	-2.89161	0.02278	2 C
1.16656	-1.13823	1.13568	2 C
2.11373	-2.09893	0.69152	2 C
2.10057	-2.11246	-0.73332	2 C
1.14412	-1.16130	-1.17811	2 C
0.56185	-0.54081	-0.02166	2 C
-0.47788	0.47547	-0.02365	2 C
-1.06369	1.09811	-1.17443	2 C
1.14760	3.47272	1.17103	2 C
0.15922	4.40690	0.74386	2 C
0.16396	4.43128	-0.67944	2 C
1.15509	3.51189	-1.13171	2 C
1.76608	2.92308	0.01197	2 C
-1.09303	1.07590	1.12349	2 C
-2.07257	2.00844	0.68278	2 C
-2.05467	2.02217	-0.74093	2 C
0.40761	-5.02869	-1.32389	3 H
-1.43443	-3.24286	-2.15903	3 H
-2.54483	-2.11273	0.02136	3 H
-1.39677	-3.20363	2.20475	3 H
0.43077	-5.00294	1.37130	3 H
2.68772	-2.76192	-1.36842	3 H
2.71296	-2.73567	1.32764	3 H
0.88902	-0.95683	-2.20879	3 H
2.52217	2.15079	0.00172	3 H
1.35618	3.19411	2.19427	3 H
-0.51300	4.95807	1.38633	3 H

-0.50338	5.00387	-1.30765	3 H
1.36947	3.26834	-2.16268	3 H
-0.78157	0.91916	-2.20219	3 H
-2.68154	2.63509	1.31889	3 H
-2.64794	2.66045	-1.38020	3 H
-0.83892	0.87620	2.15461	3 H
0.93263	-0.91223	2.16693	3 H

END

Figure S62: Comparison of XANES spectra calculated with FEFF10 with experimental static spectrum. Calculated spectra have been shifted by -10 eV to better match the experimental spectrum.

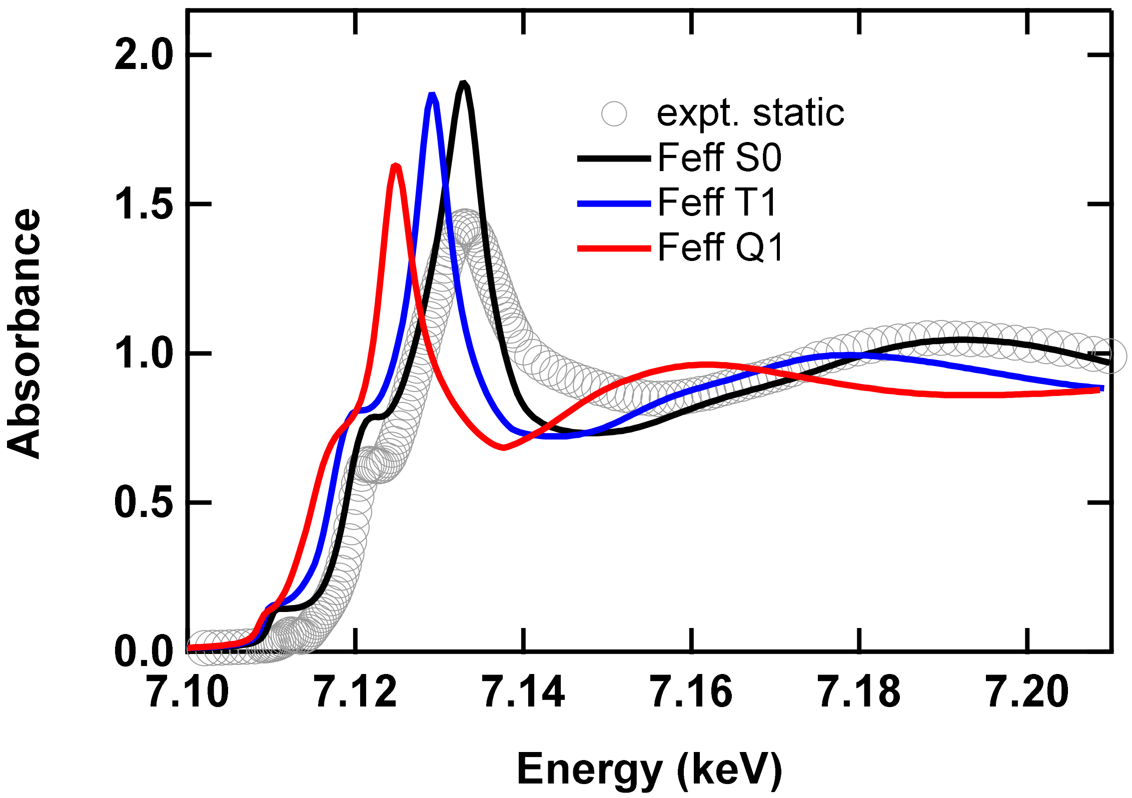

Figure S63: Comparison of EXAFS spectra calculated with FEFF10 with experimental static spectrum. Calculated spectra have been shifted by -10 eV to better match the experimental spectrum.

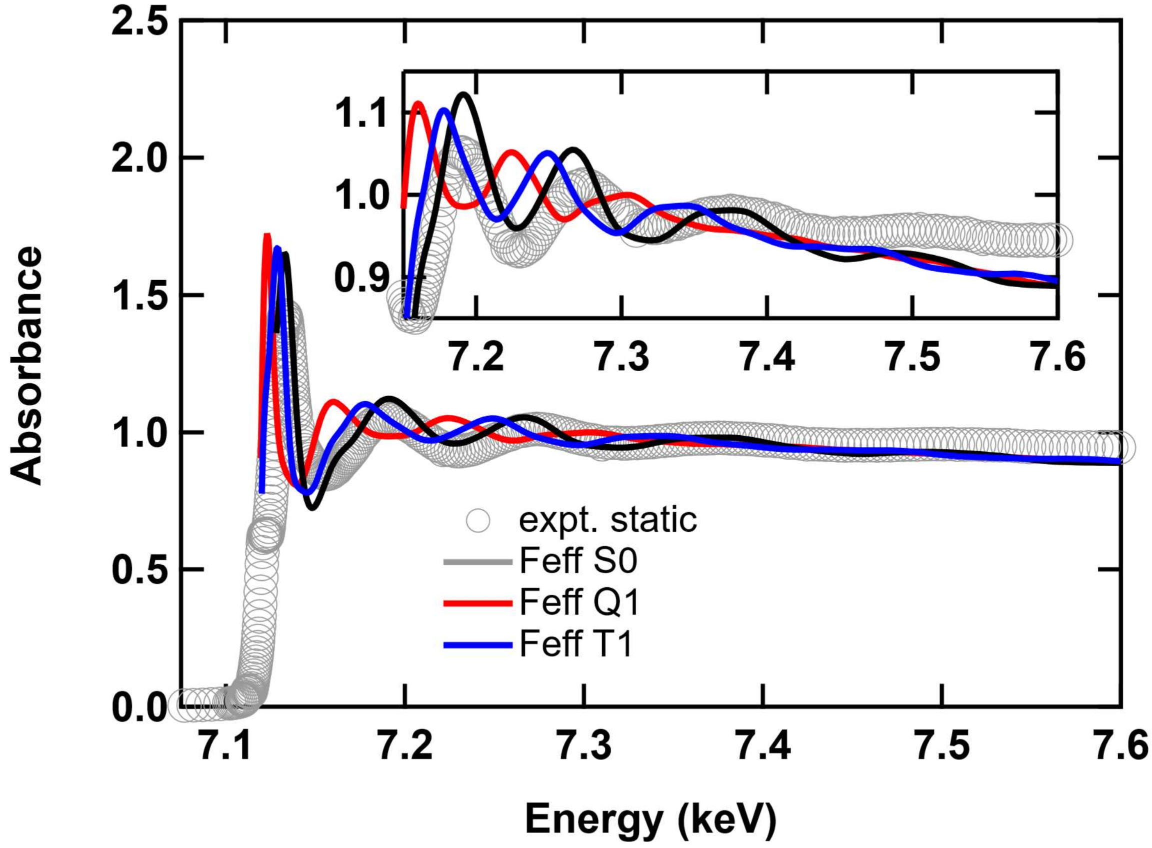

6 Estimation of Activation Energy of Triplet-Quintet Intersystem Crossing

The intersystem crossing (ISC) dynamics of $[FcCc]PF_6$ are remarkable due to the timescales involved. In the MLCT→^{3}(d-d) → ^{5}(d-d) cascade of Fe(II) spin crossover complexes, the lifetime of the intermediate ^{3}(d-d) state is as short as 39 fs.[24] In the MMCT→^{3}(d-d) → ^{5}(d-d) cascade of $[FcCc]PF_6$, the intermediate ^{3}(d-d) lifetime is 3 orders of magnitude longer, at ~30 ps. The difference in lifetime suggests that the Fe(II) ion in the D_{5h} symmetric ligand field of the ferrocene unit has a barrier to triplet-quintet ISC, which is considered to be barrierless for Fe(II) ions in O_h symmetry. We therefore sought to estimate the barrier height, or activation energy, of the intersystem crossing.

With DFT, we estimated a barrier height of 2.2 kcal/mol between the T_1 and Q_1 states (Figure S5). Here, we aim to provide an experimental estimate of the barrier height based on transition state theory and the time constant measured with OTA.

From transition state theory, the rate constant is given by

$$k = \kappa \frac{K_B T}{h} e^{\frac{\Delta S^\ddagger}{R}} e^{-\frac{\Delta H^\ddagger}{RT}}$$

Equation 6

where $\Delta S^\ddagger$ and $\Delta H^\ddagger$ are the differences in entropy and enthalpy, respectively, between the reactant and transition state, T is the temperature, K_B is the Boltzmann constant, h is the Planck constant, R is the gas constant, and κ is the transmission probability, which gives the probability that the transition state will relax to the product state instead of returning to the reactant state. Collecting the temperature-independent terms containing κ and $\Delta S^\ddagger$ into a single prefactor, A, and recognizing $\Delta H^\ddagger$ as the activation energy, E_a, we obtain the equation

$$k = A \frac{K_B T}{h} e^{-\frac{E_a}{RT}}$$

Equation 7

Converting the rate constant to a time constant through $k = 1/\tau$ and solving for the activation energy gives

$$E_a = -RT \ln\left(\frac{h}{A K_B T \tau}\right)$$

Equation 8

The time constant, τ = 30 ps, is known from OTA. The exponential prefactor, A, on the other hand, is unknown. The activation entropy, $\Delta S^\ddagger$, is likely small due to the similar structures of the ^{3}(d-d) state and transition state. Therefore, the prefactor A is likely dominated by the transmission probability, κ. Calculation of κ would require calculating spin-orbit coupling matrix

elements[25] between the ^{3}(d-d) and ^{5}(d-d) states, which often require of spin-vibronic corrections.[26] Another unknown parameter is the temperature. The OTA experiments were performed at room temperature, but photothermal heating can cause large increases in vibrational temperature, especially following nonradiative recombination.[27] We therefore explored a range of temperature and exponential prefactor values to determine the range of activation energies that might be expected based on the rate of ISC that we measured with OTA.

Figure S64: Calculation of the activation energy of ^{3}(d-d) → ^{5}(d-d) ISC with Equation 8 for a range of temperature and exponential prefactor values, with a time constant of τ = 30 ps, as measured by OTA

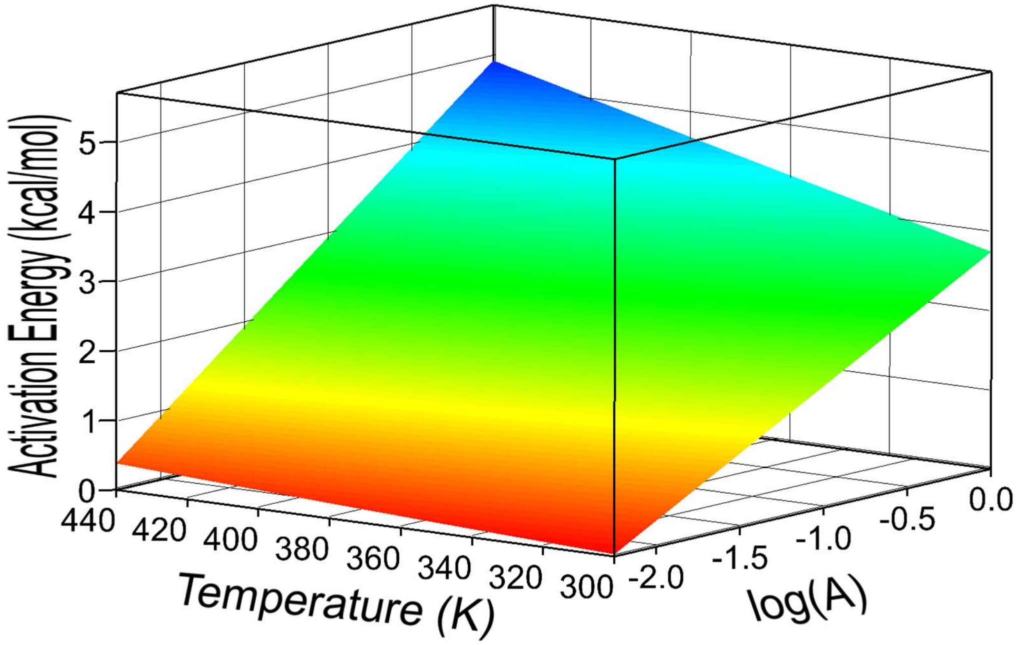

At room temperature (T = 300 K), the activation energy varies from 0.37 kcal/mol at A = 10^{-2} to 3.12 kcal/mol at A = 1. At a higher temperature of T = 400 K (100 K increase in temperature due to photothermal heating), the activation energy varies from 0.73 kcal/mol at A = 10^{-2} to 4.39 kcal/mol at A = 1. At prefactor values of A < 10^{-2}, we obtain unphysical negative activation energies. These estimates show that, despite the difficulty of calculating relative spin-state energies with DFT[28], our calculated activation energy of 2.2 kcal/mol falls within reasonable values of the parameter space. Indeed, an activation energy of 2.2 kcal/mol is obtained from Equation 8 at room temperature (300 K) with A = 2.1×10^{-1} or at 450 K with A = 4.7×10^{-2}. Overall, our OTA results, supported by this calculation, suggest that Fe(II) ions in D_{5h} ligand fields exhibit a barrier to ^{3}(d-d) → ^{5}(d-d) ISC that prevents the ultrafast ISC observed in O_h Fe(II) complexes.

7 References

(1) Schwarzhans, K.-E.; Stolz, W. Ferrocenylcobaltocenium-Hexafluorophosphat Und 1,1′-Ferrocenylendicobaltocenium-Bis-Hexafluorophosphat, Gemischtkernige Komplexe Vom Bi- Und Termetallocen-Typus. *Monatshefte Für Chem. Chem. Mon.* **1987**, *118* (8), 875–878.

(2) Warratz, R.; Peters, G.; Studt, F.; Römer, R.-H.; Tuczek, F. Orbital Interactions in Fe(II)/Co(III) Heterobimetallocenes: Single versus Double Bridge. *Inorg. Chem.* **2006**, *45* (6), 2531–2542.

(3) Becke, A. D. Density-functional Thermochemistry. III. The Role of Exact Exchange. *J. Chem. Phys.* **1993**, *98* (7), 5648–5652.

(4) Perdew, J. P.; Burke, K.; Wang, Y. Generalized Gradient Approximation for the Exchange-Correlation Hole of a Many-Electron System. *Phys. Rev. B* **1996**, *54* (23), 16533–16539.

(5) Lee, C.; Yang, W.; Parr, R. G. Development of the Colle-Salvetti Correlation-Energy Formula into a Functional of the Electron Density. *Phys Rev B* **1988**, *37* (2), 785–789.

(6) McLean, A. D.; Chandler, G. S. Contracted Gaussian Basis Sets for Molecular Calculations. I. Second Row Atoms, Z=11–18. *J. Chem. Phys.* **1980**, *72* (10), 5639–5648.

(7) Krishnan, R.; Binkley, J. S.; Seeger, R.; Pople, J. A. Self-consistent Molecular Orbital Methods. XX. A Basis Set for Correlated Wave Functions. *J. Chem. Phys.* **1980**, *72* (1), 650–654.

(8) Wachters, A. J. H. Gaussian Basis Set for Molecular Wavefunctions Containing Third-Row Atoms. *J. Chem. Phys.* **1970**, *52* (3), 1033–1036.

(9) Hay, P. J. Gaussian Basis Sets for Molecular Calculations. The Representation of 3d Orbitals in Transition-metal Atoms. *J. Chem. Phys.* **1977**, *66* (10), 4377–4384.

(10) Dunning, Thom. H.; Hay, P. J. Gaussian Basis Sets for Molecular Calculations. In *Methods of Electronic Structure Theory*; Schaefer, H. F., Ed.; Springer US: Boston, MA, 1977; pp 1–27.

(11) Hay, P. J.; Wadt, W. R. Ab Initio Effective Core Potentials for Molecular Calculations. Potentials for the Transition Metal Atoms Sc to Hg. *J. Chem. Phys.* **1985**, *82* (1), 270–283.

(12) Caricato, M. Absorption and Emission Spectra of Solvated Molecules with the EOM–CCSD–PCM Method. *J. Chem. Theory Comput.* **2012**, *8* (11), 4494–4502.

(13) Ochterski, J. W. *Thermochemistry in Gaussian*. https://gaussian.com/thermo/ (accessed 2024-04-16).

(14) *Creating UV/Visible Plots from the Results of Excited States Calculations*. https://gaussian.com/uvvisplot/ (accessed 2024-04-16).

(15) Livshits, M. Y.; Turlington, M. D.; Trindle, C. O.; Wang, L.; Altun, Z.; Wagenknecht, P. S.; Rack, J. J. Picosecond to Nanosecond Manipulation of Excited-State Lifetimes in Complexes with an FeII to TiIV Metal-to-Metal Charge Transfer: The Role of Ferrocene Centered Excited States. *Inorg. Chem.* **2019**, *58* (22), 15320–15329.

(16) Carlton, E. S.; Sutton, J. J.; Gale, A. G.; Shields, G. C.; Gordon, K.; Wagenknecht, P. S. Insights into the Charge-Transfer Character of Electronic Transitions in RCp2Ti(C2Fc)2 Complexes Using Solvatochromism, Resonance Raman Spectroscopy, and TDDFT. *Dalton Trans* **2021**.

(17) Bearden, J. A.; Burr, A. F. Reevaluation of X-Ray Atomic Energy Levels. *Rev. Mod. Phys.* **1967**, *39* (1), 125–142.

(18) Ruiz-Lopez, M. F.; Loos, M.; Goulon, J.; Benfatto, M.; Natoli, C. R. Reinvestigation of the EXAFS and Xanes Spectra of Ferrocene and Nickelocene in the Framework of the Multiple Scattering Theory. *Chem. Phys.* **1988**, *121* (3), 419–437.

(19) Islam, M. T.; Best, S. P.; Bourke, J. D.; Tantau, L. J.; Tran, C. Q.; Wang, F.; Chantler, C. T. Accurate X-Ray Absorption Spectra of Dilute Systems: Absolute Measurements and Structural Analysis of Ferrocene and Decamethylferrocene. *J. Phys. Chem. C* **2016**, *120* (17), 9399–9418.
(20) Gawelda, W.; Pham, V.-T.; van der Veen, R. M.; Grolimund, D.; Abela, R.; Chergui, M.; Bressler, C. Structural Analysis of Ultrafast Extended X-Ray Absorption Fine Structure with Subpicometer Spatial Resolution: Application to Spin Crossover Complexes. *J. Chem. Phys.* **2009**, *130* (12), 124520.
(21) William H. Press; Saul A. Teukolsky; William T. Vetterling; Brian P. Flannery. *Numerical Recipes in C: The Art of Scientific Computing*, 2nd ed.; CAMBRIDGE UNIVERSITY PRESS: New York, 1992.
(22) Kas, J. J.; Vila, F. D.; Pemmaraju, C. D.; Tan, T. S.; Rehr, J. J. Advanced Calculations of X-Ray Spectroscopies with FEFF10 and Corvus. *J. Synchrotron Radiat.* **2021**, *28* (6), 1801–1810.
(23) Rehr, J. J.; Kas, J. J.; Vila, F. D.; Prange, M. P.; Jorissen, K. Parameter-Free Calculations of X-Ray Spectra with FEFF9. *Phys. Chem. Chem. Phys.* **2010**, *12* (21), 5503–5513.
(24) Zhang, K.; Ash, R.; Girolami, G. S.; Vura-Weis, J. Tracking the Metal-Centered Triplet in Photoinduced Spin Crossover of Fe(Phen)32+ with Tabletop Femtosecond M-Edge X-Ray Absorption Near-Edge Structure Spectroscopy. *J. Am. Chem. Soc.* **2019**, *141* (43), 17180–17188.
(25) Harvey, J. N. Understanding the Kinetics of Spin-Forbidden Chemical Reactions. *Phys. Chem. Chem. Phys.* **2007**, *9* (3), 331–343.
(26) Penfold, T. J.; Gindensperger, E.; Daniel, C.; Marian, C. M. Spin-Vibronic Mechanism for Intersystem Crossing. *Chem. Rev.* **2018**, *118* (15), 6975–7025.
(27) Lynch, M. S.; Van Kuiken, B. E.; Daifuku, S. L.; Khalil, M. On the Role of High-Frequency Intramolecular Vibrations in Ultrafast Back-Electron Transfer Reactions. *J. Phys. Chem. Lett.* **2011**, *2* (17), 2252–2257.
(28) Reiher, M.; Salomon, O.; Artur Hess, B. Reparameterization of Hybrid Functionals Based on Energy Differences of States of Different Multiplicity. *Theor. Chem. Acc.* **2001**, *107* (1), 48–55.
